# Design, synthesis and anti-breast cancer activity evaluation of 6,7-dihydro-5*H*-pyrrolo[3,4-*d*]pyrimidine-based PARP1/ATR dual inhibitors

**DOI:** 10.1080/14756366.2026.2627053

**Published:** 2026-02-16

**Authors:** Meng-Lan He, Zong-Hao Wang, Xia Yao, Lu-Lu Ye, Bo-Qun Du, Chen-Chen Wang, Yong-Hao Chen, Xiao-Xian Wang, Hui Luo, Yuan Gao, Xiang-Yang Ye

**Affiliations:** a School of Pharmacy, Hangzhou Normal University, Hangzhou, China; b Zhejiang Provincial Key Laboratory of Anti-Cancer Chinese Medicines and Natural Medicines, Hangzhou Normal University, Hangzhou, China; c College of Life and Environmental Sciences, Hangzhou Normal University, Hangzhou, China; d School of Clinical Medicine, Hangzhou Normal University, Hangzhou, China; e Clinical Research Institute, Zhejiang Provincial People’s Hospital (Affiliated People’s Hospital), Hangzhou Medical College, Hangzhou, P. R. China

**Keywords:** PARP1, ATR, dual inhibitors, breast cancer, anticancer

## Abstract

PARP1 inhibitors are FDA-approved for BRCA1/2-mutated breast cancer but show limited efficacy in wild-type cancers and face resistance issues. To overcome these, we designed novel 6,7-dihydro-5*H*-pyrrolo[3,4-*d*]pyrimidine-based compounds integrating PARP1 inhibitor pharmacophores with the ATR inhibitor AZD6738 scaffold. Substituent modifications influenced PARP1 and ATR selectivity, yielding dual inhibitors or selective PARP1 inhibitors. Compound **38a**, the lead candidate, exhibited potent dual inhibition (IC_50_ < 20 nM) and strong antitumor effects in MDA-MB-231 (IC_50_ < 0.048 μM) and MDA-MB-468 (IC_50_: 0.01 μM) cell lines *in vitro*. Mechanistically, **38a** arrested cell cycle progression, induced apoptosis, inhibited colony formation and migration, and suppressed DNA damage repair pathways, outperforming combined Niraparib and AZD6738. These findings underscore the therapeutic potential of PARP1/ATR dual inhibitors for breast cancer and support further investigation.

## Introduction

**Figure 1.**
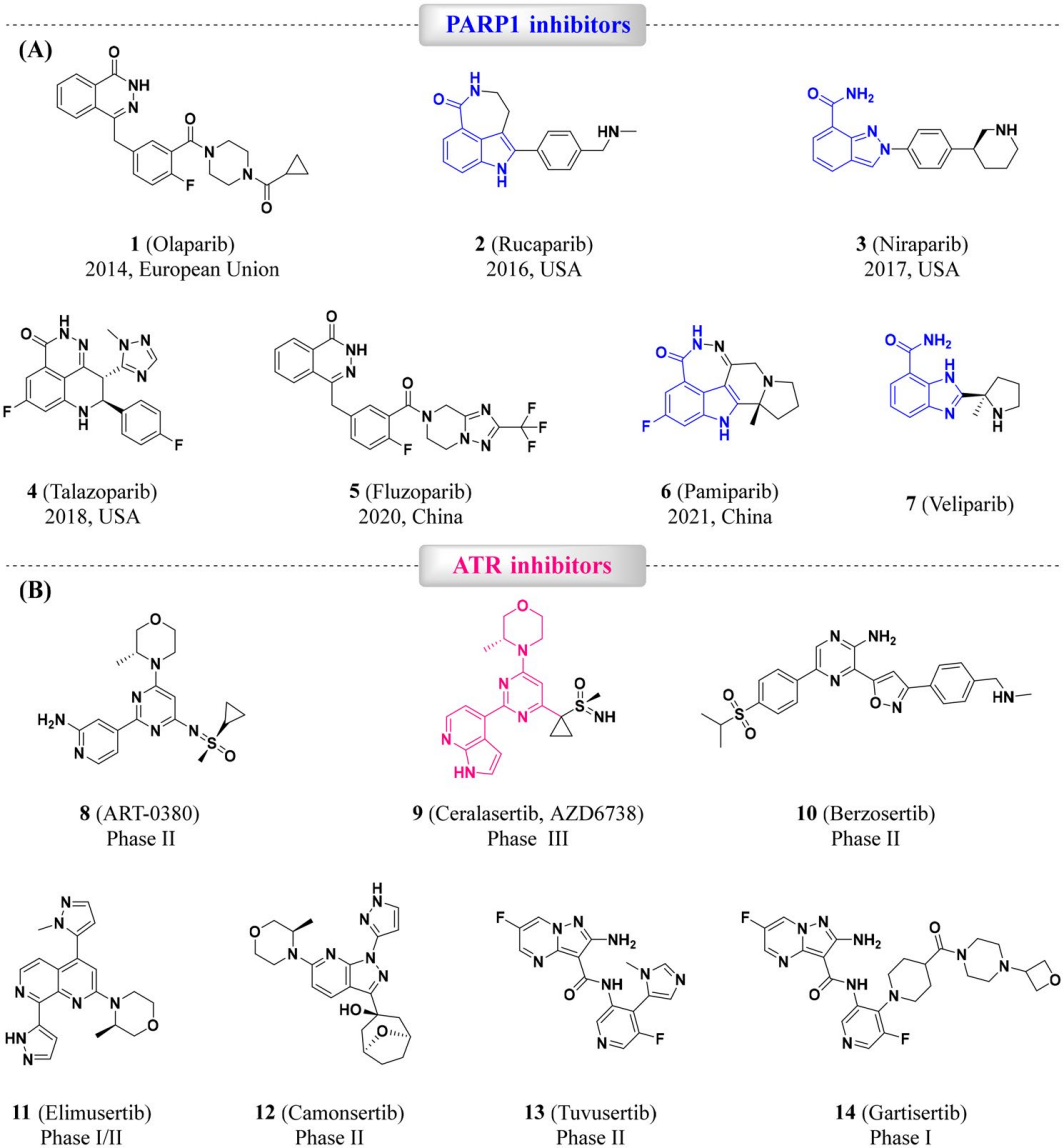
Chemical structures of PARPi and ATRi. (A) PARPi approval drugs **1**–**6** and a representative PARPi clinical candidate **7**; (B) representative ATRi clinical candidates **8**–**14**.

Breast cancer remains a leading cause of mortality among women worldwide, despite significant advancements in therapeutic strategies.^1^^,^^2^ Suboptimal treatment outcomes and acquired drug resistance contribute to limiting therapeutic strategy applications and clinical success, underscoring the urgent need for novel therapeutic targets and approaches. PARP1 plays an important role in DNA damage repair (DDR). mechanistically, PARP1 facilitates single-strand break (SSB) repair via the base excision repair (BER) pathway by catalysing protein ADP-ribosylation.^3^ PARP1 inhibitors (PARPi) exploit this role to induce synthetic lethality in BRCA1/2-mutated tumours, where homologous recombination repair (HRR) is compromised.^3–6^ Clinically approved PARPi, such as Olaparib (**1**), Rucaparib (**2**), Niraparib (**3**), Talazoparib (**4**), Fluzoparib (**5**), Pamiparib (**6**), and Veliparib (**7**) (Figure 1(A)), have transformed treatment for BRCA1/2-mutated breast and ovarian cancers.^7^^,^^8^ However, resistance to PARPi monotherapy, driven by mechanisms such as HRR restoration, replication fork stabilisation, or alternative DDR activation, limits their efficacy, particularly in triple-negative breast cancer (TNBC) with BRCA wild-type.^9–11^

To address these limitations, dual-target inhibition strategies have emerged to enhance synthetic lethality and overcome resistance. A promising approach involves co-targeting PARP1 and ATR kinase, a master regulator of replication stress response and G2/M checkpoint control.^12^ PARP1 inhibition induces replication stress and DNA damage, which activates ATR to stabilise replication forks and promote cell survival.^13^^,^^14^ In tumours with ATM deficiency or other DDR defects, ATR becomes critical for genomic stability, making it a compelling complementary target to PARP1.^15^ Notably, PARP1 inhibitors (e.g. Olaparib) upregulate ATR phosphorylation and G2/M arrest, highlighting ATR’s role in mediating resistance to PARPi.^16^

Although no ATR inhibitors have received regulatory approval, several candidates, including ART-380 (**8**), AZD6738 (Ceralasertib, **9**), Berzosertib (M6620, **10**, also known as M6620/VX-970), Elimusertib (**11**), Camonsertib (RP-3500, **12**), Tuvusertib (M1774, **13**), and Gartisertib (M4344/VX-803, **14**) (Figure 1(B)), are progressing into clinical trials.^17^ These agents disrupt ATR-mediated cell cycle checkpoints, forcing p53-deficient tumour cells with unrepaired DNA damage into premature mitosis, resulting in mitotic catastrophe.^18^ The synergistic potential of dual PARP1/ATR inhibition lies in its ability to block compensatory DDR pathways, promoting tumour cell death and mitigating resistance.

Building on Wang et al.’s work on BRD4/PARP dual inhibitors^19^ and our prior work with PARP- and/or ATR-related inhibitors and bifunctional conjugates,^20–29^ we report a novel series of 6,7-dihydro-5*H*-pyrrolo[3,4-*d*]pyrimidine-based dual inhibitors designed to synergistically modulate PARP1 and ATR activities. These compounds aim to address the unmet needs in TNBC, particularly in BRCA wild-type contexts. In which, compound **38a** exhibits significant inhibition of MDA-MB-231 and MDA-MB-468 cell lines’ proliferation compared to combined Niraparib and AZD6738. This study presents their design, synthesis, and evaluation, demonstrating enhanced anti-breast cancer efficacy.

## Results and discussion

### Combination of Niraparib and AZD6738 synergistically Inhibits the proliferation of MDA-MB-231 cells

**Figure 2.**
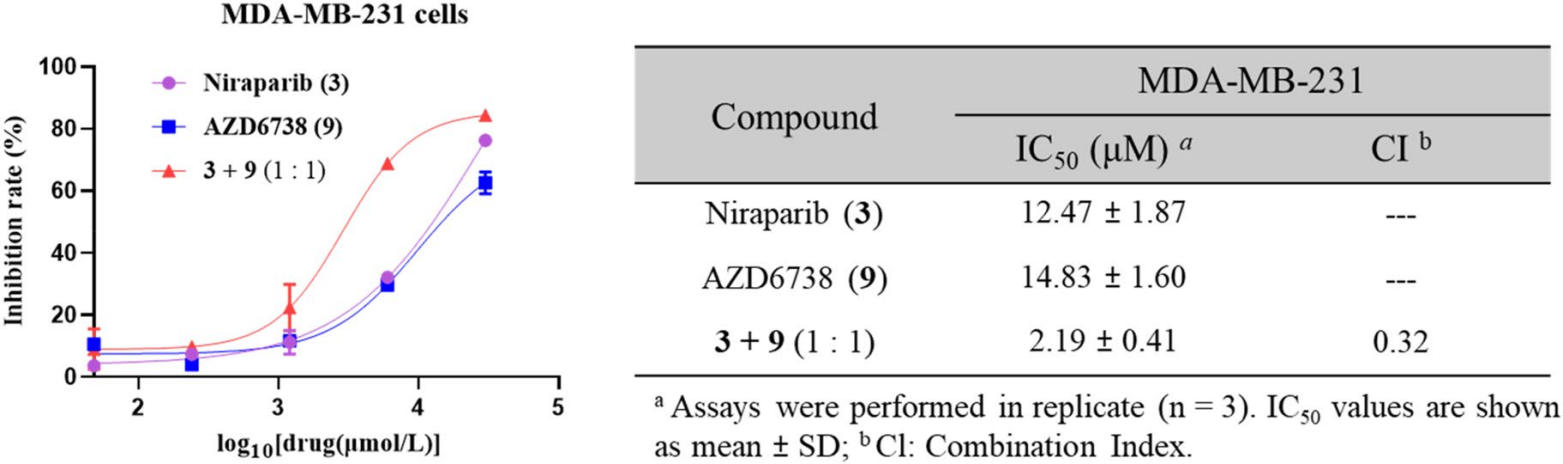
The IC_50_ and combination index (CI) of Niraparib and AZD6738 on MDA-MB-231 cells *in vitro.*

To investigate the synergistic effects of combining PARPi and ATRi, we evaluated the clinically approved PARPi Niraparib (**3**) and ATRi AZD6738 (**9**) for their combined impact on the proliferation of TNBC cell line MDA-MB-231. Our results showed that co-administration of Niraparib and AZD6738 at a 1: 1 molar ratio produced significant synergistic anti-proliferative activity, with a markedly enhanced effect compared to either agent alone (IC_50_: 2.19 *vs* 12.47 μM for Niraparib and 14.83 μM for AZD6738). Quantitative assessment using the combination index (CI) method confirmed this synergy, with a CI value of 0.32 (Figure 2). These findings underscore the potential of dual PARP1/ATR inhibitors to achieve synergistic antitumor efficacy, supporting their further development.

### Rational design of PARP/ATR dual inhibitors

**Figure 3.**
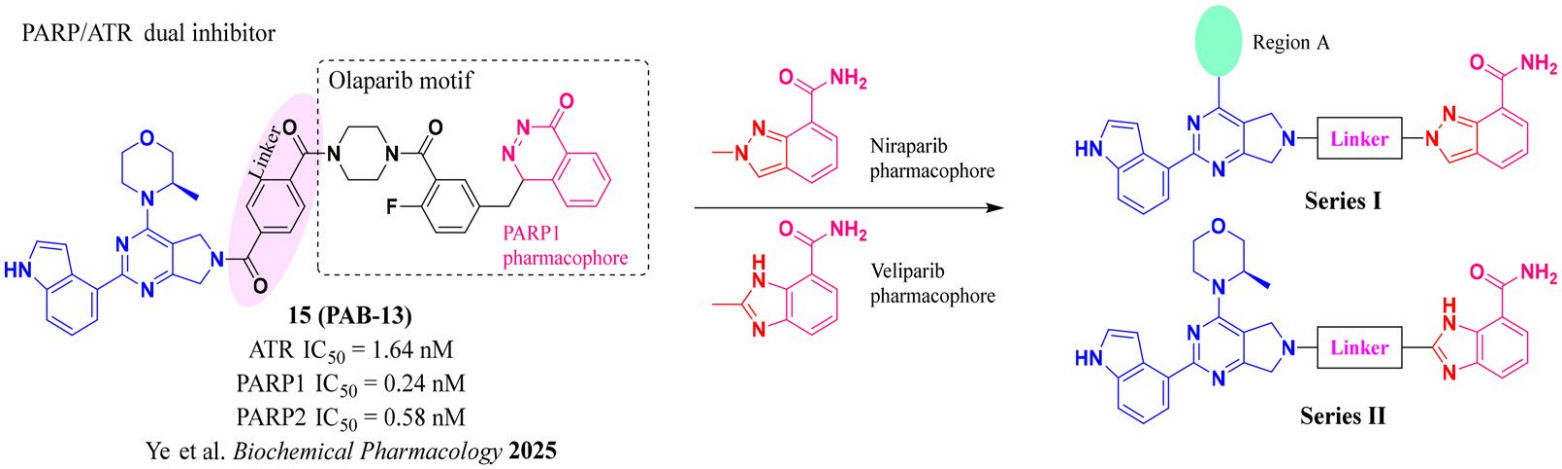
Strategy for the development of novel PARP1/ATR dual inhibitors from **15** (PAB-13).

We previously reported PARP1/ATR dual inhibitor **15** (PAB-13, Figure 3) derived from AZD6738 (**9**) and Olaparib (**1**).^19^^,^^22^ This compound is a potent dual inhibitor against both ATR and PARP1/2, and also shows significant tumour growth suppression in animal models, though it has poor pharmacokinetic property. To search for other structurally diverse PARP1/ATR dual inhibitors with possible improved physical chemical properties and PK profile, we decided to replace PARP1 pharmacophore phthalazin-1(4*H*)-one with others such as the ones in Niraparib or Veliparib. Thus, the replacement ended up with two general structures: Series I and Series II (Figure 3). In Series I, region A was intended for exploration of replacement of (*R*)-3-methylmorpholine motif.

### Chemical Synthesis of PARP1/ATR dual inhibitors

**Scheme 1.**
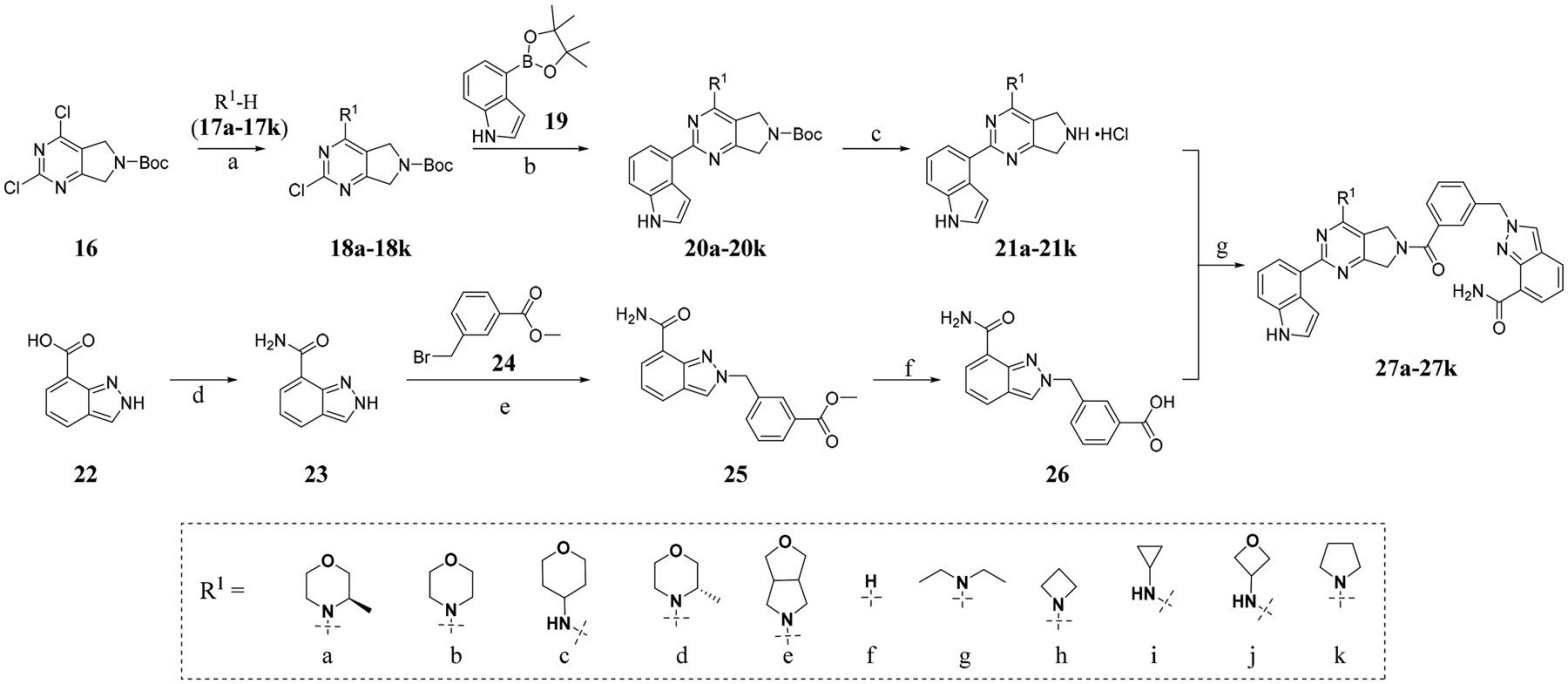
Synthesis of target compounds **27a-27k**. ^a^Reagents and conditions: (a) DIPEA, DMF, 50 °C,5 h, 60%−85%; (b) K_3_PO_4_, Pd(PPh_3_)_4_, 1,4-dioxane, H_2_O, 100 °C, 10 h, 64%−80%; (c) HCl in 1,4-dioxane (4 M), MeOH, rt, 12 h, 95%−99%; (d) SOCl_2_, DMF, NH_3_·H_2_O, THF, 50%; (e) K_2_CO_3_, DMF, 90 °C, 2 h, 67%; (f) LiOH, THF, 92%; (g) HOBt, EDCI, DIPEA, DMF, rt, 4 h, 10%−35%.

We focused on 3-carbonyl benzyl group as linker to design Series I compounds based on our experience in PARP1/ATR dual inhibitors.^19^ Several diverse groups were chosen for “R” (Scheme 1) in order to explore the structure-activity relationship of Region A in Series I. Target compounds **27a**-**27k** were synthesised using standard amide coupling reaction, nucleophilic displacement reaction and protecting group deprotection reaction (Scheme 1). Commercially available materials **16** and **17a-17k** first underwent nucleophile displacement reaction to yield intermediates **18a-18k**, which then reacted with **19** *via* a Suzuki coupling reaction to form intermediates **20a-20k**. Subsequently, the Boc-protecting groups in **20a-20k** were deprotected to afford **21a-21k**. On the other hand, commercially available 1*H*-indazole-7-carboxylic acid (**22**) was transformed to amide using standard amide formation protocol to afford **23**, whose pyrazole nitrogen was alkylated with benzyl bromide **24** to afford **25**. The ester group of **25** was deprotected to form acid **26**. Finally, intermediates **21a-21k** reacts with intermediate **26** in the presence of HOBt and EDCI to form target compounds **27a-27k** respectively.

**Scheme 2.**
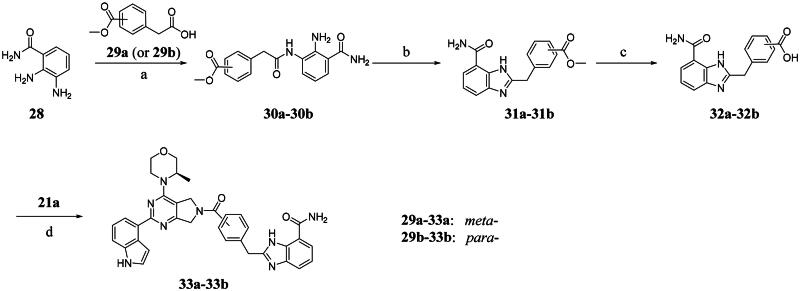
Synthesis of compounds **33a** and **33b**. ^a^Reagents and conditions: (a) HOBt, EDCI, DIPEA, DMF, rt, 4.5 h, 45%−70%; (b)CH_3_CO_2_H, 120 °C, 2 h, 80%−92%; (c) NaOH, MeOH: H_2_O = 4: 1, rt, 4 h, 80%−90%; (d) HOBt, EDCI, DIPEA, DMF, rt, 4.5 h, 20%−46%.

The biological assessment of Series I compounds (Table 1) suggested that **27a** was a better hit than other analogs due to its good inhibitory activity against ATR, PARP7, and PARP1 (IC_50_: 9, 4.4, and 907 nM, respectively). Therefore, we decided to design Series II compounds based on the results. Initially, two Series II compounds (i.e. **33a** and **33b**, Scheme 2) were designed from **27a** by changing its PARP pharmacophore, i.e. 1*H*-indazole-7-carboxamide motif (**27a**) being replaced with 1*H*-benzo[*d*]imidazole-7-carboxamide (i.e. veliparib pharmacophore). Thus, commercially available intermediate **28** reacted with **29a** or **29b** *via* amide formation reaction to afford key intermediates **30a** or **30b**, respectively. The intermediates underwent intramolecular cyclisation reaction, followed by ester hydrolysis to yield carboxylic acid intermediates **32a-32b**. The final step was the amide formation reaction between **32a** (or **32b**) with **21a** to afford **33a** (or **33b**).

**Table 1.** Inhibitory activities of series I compounds against PARP1, PARP7 and ATR kinase.

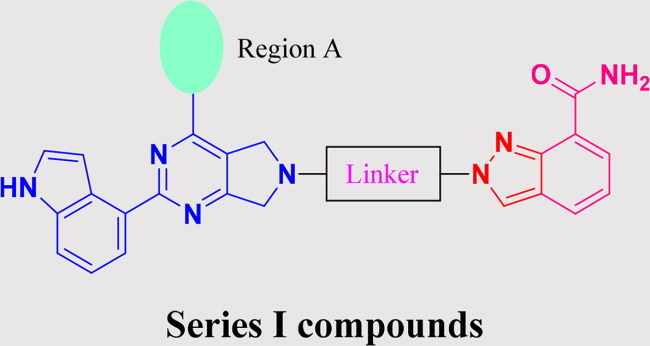					
Compd.	Region A	Linker	IC_50_ (nM)^*a*^		
			PARP1	PARP7	ATR
**27a**	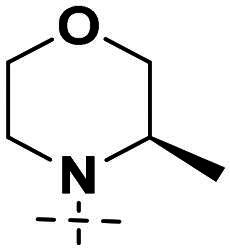	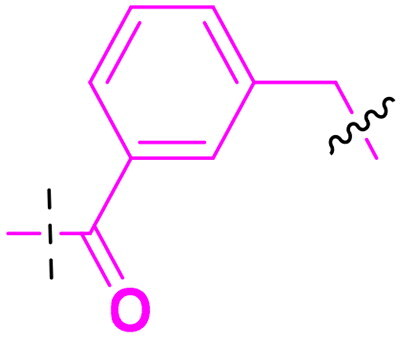	907.00 ± 37.47	4.40 ± 0.35	9.00 ± 1.32
**27b**	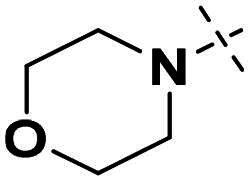		261.00 ± 32.02	5.60 ± 0.01	79.70% @ 1000
**27c**	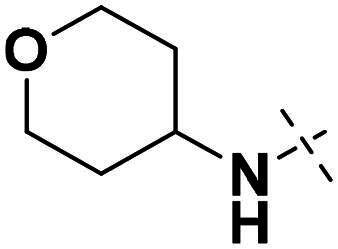		45.00 ± 10.34	4.10 ± 0.13	22.10% @ 1000
**27d**	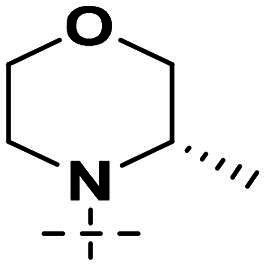		133.00 ± 23.46	130.00 ± 18.44	36.30% @ 1000
**27e**	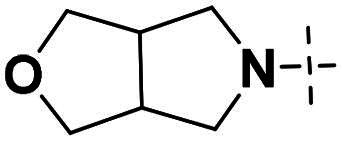		> 10	4.10 ± 1.49	3.00 ± 0.01
**27f**	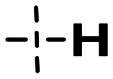		> 1250	19.00 ± 6.50	1.61% @ 1000
**27 g**	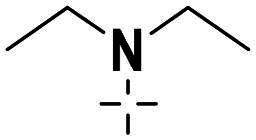		< 10	10.00 ± 1.67	> 500
**27h**	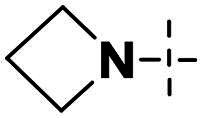		8.4% @ 10	7.30 ± 2.66	−4.75% @ 1000
**27i**	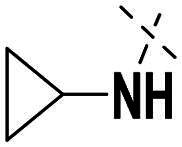		2.9% @ 10	9.30 ± 1.45	15.70% @ 1000
**27j**	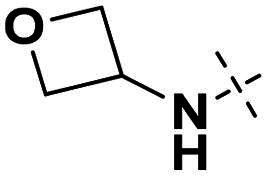		3.8% @ 10	33.00 ± 8.32	1.61% @ 1000
**27k**	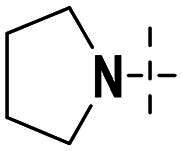		> 25	16.00 ± 9.23	−3.57% @ 1000
**Niraparib**	/	/	8.20 ± 1.37	/	/
**AZD6738**	/	/	/	/	5.60 ± 0.23

^a^ Assays were performed in replicate (*n* = 3); IC_50_ values are shown as mean ± SD.

**Scheme 3.**
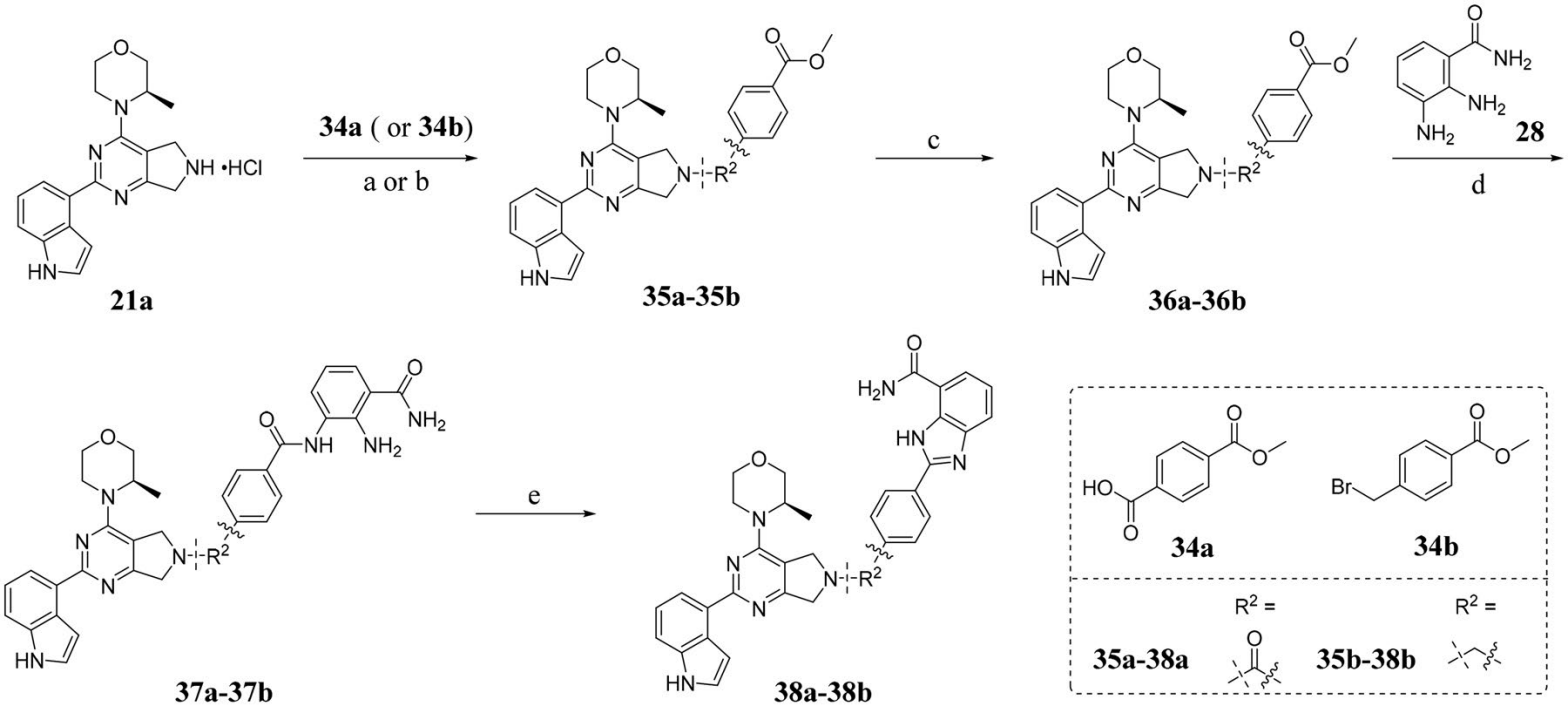
Synthesis of target compounds 38a-38b. ^a^Reagents and conditions: (a) HOBt, EDCI, DIPEA, DMF, rt, 4.5 h, 82%; (b) DIPEA, DMF, 50 °C, 5 h, 67%; (c) NaOH, MeOH: H_2_O = 4: 1, rt, 4 h, 80%−95%; (d) HOBt, EDCI, DIPEA, DMF, rt, 4.5 h, 60%−70%;(e) CH_3_CO_2_H, 120 °C., 2 h, 25%−35%.

Compounds **38a** and **38b**, a subset of Series II with linkers different from **33a** and **33b**, were synthesised using the route illustrated in Scheme 3. The first step is the acylation (or alkylation) of intermediate **21a** with **34a** (or **34b**) under standard conditions to afford **35a-35b**. The ester group was hydrolysed under basic condition to give acids **36a** and **36b**. The amide formation reaction between the acid (**36a** or **36b**) and **28** followed by ring cyclisation under acidic condition afford **38a** and **38b** respectively.

**Scheme 4.**
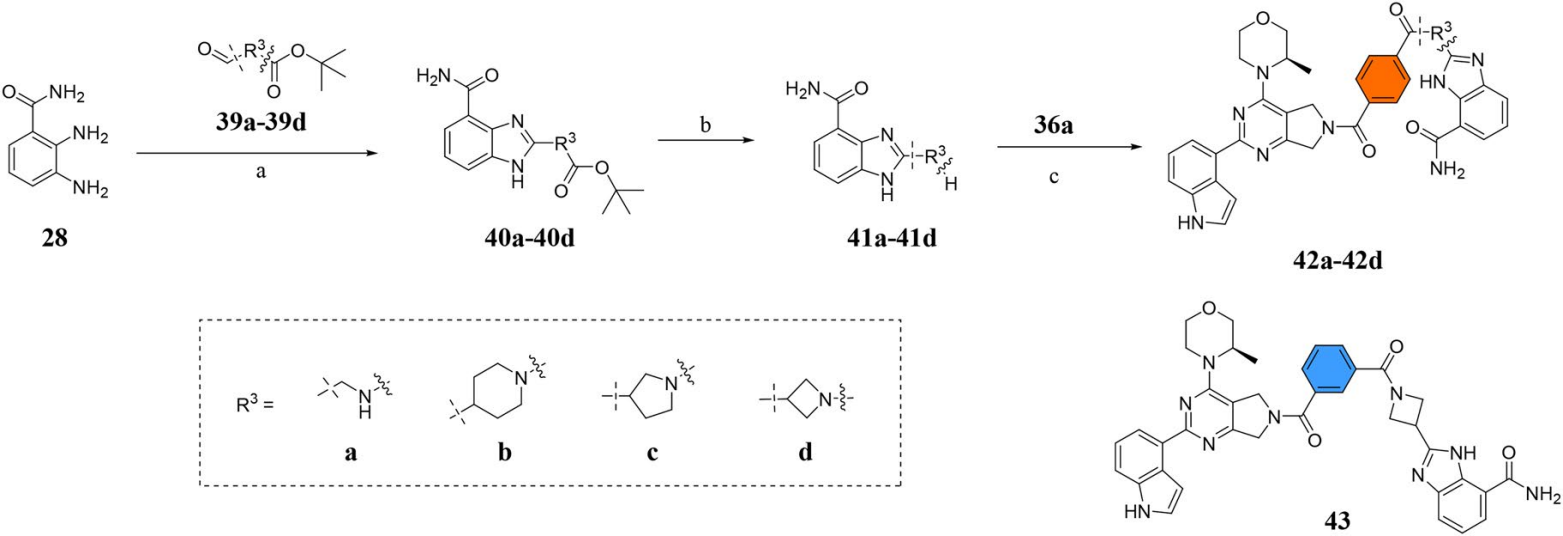
Synthesis of target compounds **42a-42d**, **43**. ^a^Reagents and conditions: (a) Pd/C, MeOH, 80 °C, 8 h, 40%−55%; (b) HCl in Dioxane (4 M), MeOH, rt, 12 h, 85%−95%; (c) HOBt, EDCI, DIPEA, DMF, rt, 4.5 h, 20%−40%.

Next, we designed a subset of Series II, in which the linker consisting of both an aryl group (*meta*-substituted or *para*-substituted) and an alkylamino group or cycloamino group (i.e. compounds **42a-42d**, **43** Scheme 4). The synthetic route for these compounds was adopted from Schemes 1–3 with slight modifications (Scheme 4). Starting with Boc-protected aminoaldehyde **39a** (or its analogs **39b**-**39d**), reductive amination with **21a** under Pd/C afforded **40a** (or its analogs **40b**-**40d** respectively). The Boc protecting group was then removed under acidic condition to give amine **41a** (or **41b**-**41d**), which underwent amide formation reaction with acid **36a** (Scheme 3) to afford desired product **42a** (or **42b**-**42d, 43** respectively).

### Inhibitory activities of compounds against PARP1, PARP7 and ATR

In this study, a series of 6,7-dihydro-5*H*-pyrrolo[3,4-*d*]pyrimidine derivatives designated as Series I (compounds **27a**–**27k**) were evaluated for their inhibitory activities against PARP1, PARP7, and ATR kinases using *in vitro* enzyme assays. The results are summarised in Table 1, with IC_50_ values reported in nM (mean ± SD from triplicate experiments) or as percent inhibition at specified concentrations where full dose-response curves were not determined due to low activity. Structural variations in Region A were explored to probe structure-activity relationships (SAR), aiming to balance dual inhibition of PARP1 and ATR while assessing selectivity over PARP7. Niraparib and AZD6738 were included as reference inhibitors for PARP1 and ATR, respectively.

Compound **27a**, featuring an (*R*)-3,4-dimethylmorpholine moiety in Region A, exhibited moderate inhibition of PARP1 (IC_50_: 907 nM) and potent activity against PARP7 (IC_50_: 4.40 nM), alongside notable ATR inhibition (IC_50_: 9.00 nM), comparable to the reference AZD6738 (IC_50_: 5.60 nM). Removal of 4-methyl group in morpholine ring in **27a** (i.e. **27b**) improved PARP1 potency (IC_50_: 261 nM) and maintained strong PARP7 inhibition (IC_50_: 5.60 nM), but attenuated ATR activity, showing only 79.70% inhibition at 1000 nM. Compound **27c**, incorporating a tetrahydro-2*H*-pyran-4-amine ring, further enhanced PARP1 inhibition (IC_50_: 45 nM) while preserving potent PARP7 activity (IC_50_: 4.10 nM); however, ATR inhibition was weak (22.1% at 1000 nM). In contrast, the (*S*)-3,4-dimethylmorpholine analog **27d** displayed intermediate PARP1 potency (IC_50_: 133 nM) but significantly reduced PARP7 inhibition (IC_50_: 130 nM) and minimal ATR activity (36.3% at 1000 nM).

Shifting to more diverse substituents, compound **27e** with a 5-methyl-5*H*-furan-3,4-*c*-pyrrolo[3,4-*c*]pyrrole ring showed weak PARP1 inhibition (IC_50_ > 10 nM) but retained strong PARP7 potency (IC_50_: 4.10 nM) and excellent ATR activity (IC_50_: 3.00 nM), surpassing AZD6738. The unsubstituted analog **27f** exhibited poor PARP1 inhibition (IC_50_ > 1250 nM), moderate PARP7 activity (IC_50_: 19 nM), and negligible ATR effects (1.61% at 1000 nM). Compound **27 g**, bearing an *N*-ethyl-*N*-methylalkylamine group, achieved potent PARP1 inhibition (IC_50_ < 10 nM) with intermediate PARP7 activity (IC_50_: 10 nM), but ATR potency was abolished (IC_50_ > 500 nM), highlighting a trade-off in dual targeting.

Further modifications emphasised selectivity trends. Compound **27h**, with a 1-methylazacyclohexane ring, displayed negligible PARP1 inhibition (8.4% at 10 nM) and ATR activity (−4.75% at 1000 nM, indicating no effect), but maintained good PARP7 potency (IC_50_: 7.30 nM). Similarly, compounds **27i** (2.9% PARP1 inhibition at 10 nM; PARP7 IC_50_: 9.30 nM; 15.70% ATR inhibition at 1000 nM), **27j** (3.8% PARP1 inhibition at 10 nM; PARP7 IC_50_: 33 nM; 1.61% ATR inhibition at 1000 nM), and **27k** (PARP1 IC_50_ > 25 nM; PARP7 IC_50_: 16 nM; no inhibition against ATR at 1000 nM) showed generally low activity against PARP1 and ATR, with varying degrees of PARP7 inhibition.

Overall, the SAR analysis of Series I reveals that morpholine-based substituents (e.g. in **27a**–**27d**) tend to support balanced PARP1 and PARP7 inhibition, with stereochemistry influencing ATR potency. More rigid or unsubstituted rings (e.g. **27e**–**27f**) can enhance ATR selectivity but often at the expense of PARP1 activity, while alkylamine or azacyclic modifications (e.g. **27 g**–**27k**) favour PARP1 or PARP7 over ATR. These findings underscore the potential for fine-tuning Region A to achieve dual PARP1/ATR inhibition, as exemplified by leads like **27a**, and informed subsequent optimisation efforts leading to compound **38a**.

Building on the structure-activity relationship (SAR) insights from Series I compounds, Series II compounds (**33a**–**33b**, **38a**–**38b**, **42a**–**42d, 43**) were designed to further optimise dual PARP1/ATR inhibition while minimising PARP7 activity. These compounds incorporated a linker connecting the ATR-targeting and PARP-targeting pharmacophores, allowing exploration of how linker modifications and substituent variations influence selectivity and potency. The inhibitory activities of Series II against PARP1, PARP7, and ATR were evaluated *in vitro*, with IC_50_ values reported as mean ± SD from triplicate assays (Table 2). Niraparib and AZD6738 served as reference inhibitors for PARP1 and ATR, respectively.

**Table 2.** Inhibitory activities of series II compounds against PARP1, PARP7 and ATR kinase.

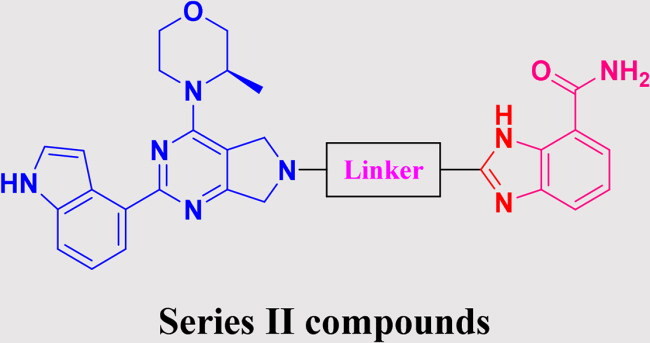				
Compd.	Linker	IC_50_ (nM)^a^		
		PARP1	PARP7	ATR
**33a**	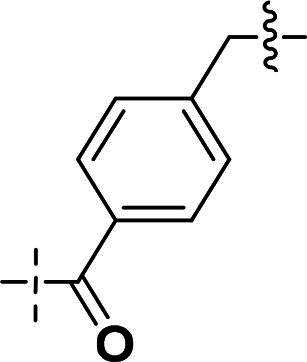	101.00 ± 15.33	7.20 ± 2.43	14.60 ± 2.47
**33b**	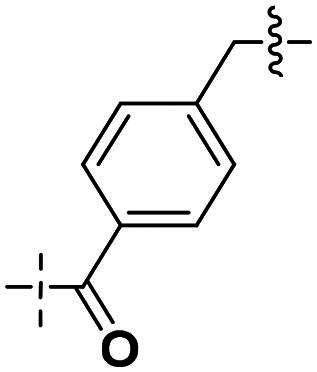	18.00 ± 6.28	44.00 ± 5.22	13.40 ± 3.28
**38a**	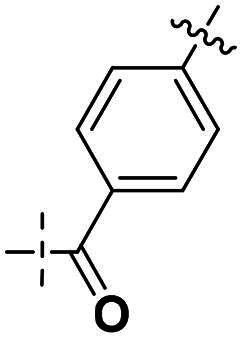	5.00 ± 2.93	> 1000	14.70 ± 2.01
**38b**	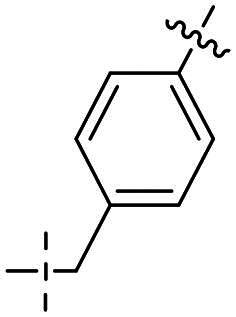	8.50 ± 0.34	> 1000	27.70 ± 5.29
**42a**	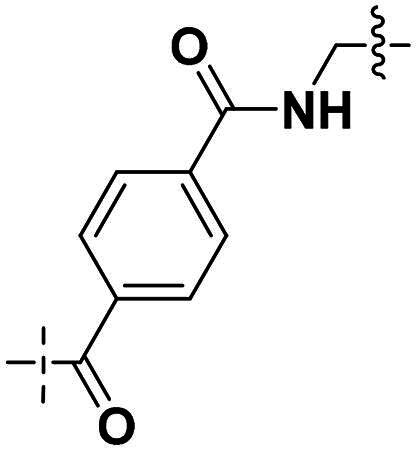	50.00 ± 3.54	19.00 ± 2.44	3.95 ± 0.39
**42b**	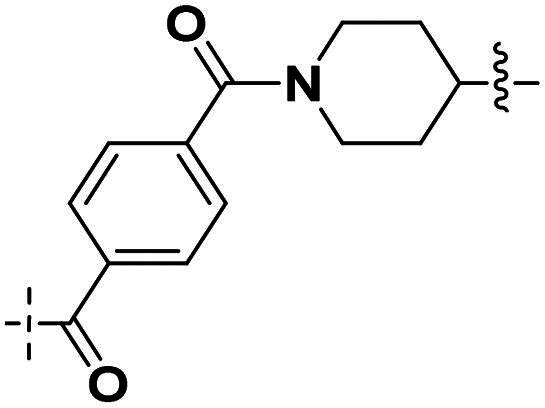	5.40 ± 0.59	48.00 ± 9.30	2.94 ± 0.02
**42c**	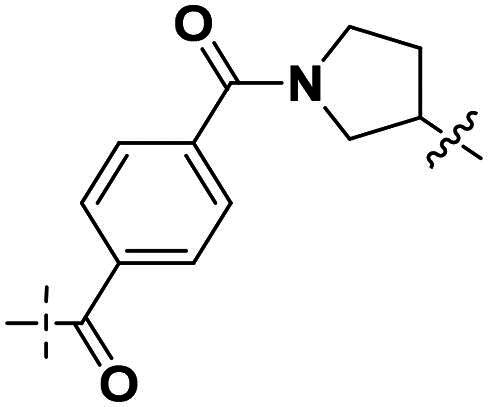	0.97 ± 0.30	150.00 ± 18.05	10.00 ± 1.95
**42d**	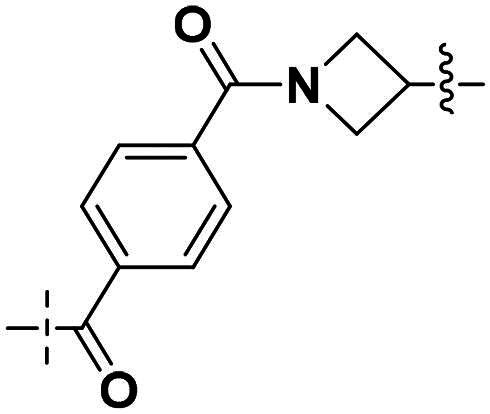	13.00 ± 0.50	35.00 ± 8.40	4.99 ± 0.52
**43**	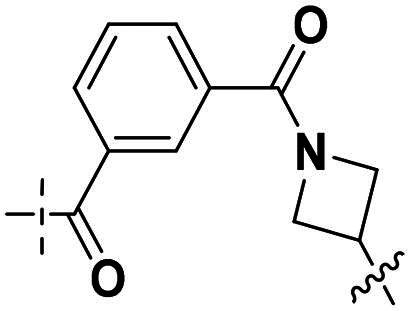	5.80 ± 1.43	103.00 ± 18.01	21.00 ± 5.35
**Niraparib**	—	8.20 ± 1.37	/	/
**AZD6738**	—	/	/	5.60 ± 0.23

^a^ Assays were performed in replicate (*n* = 3); IC_50_ values are shown as mean ± SD.

Compound **33a**, featuring a methylene linker, exhibited moderate inhibition of PARP1 (IC_50_: 101 nM) and ATR (IC_50_: 14.60 nM), with potent PARP7 activity (IC_50_: 7.20 nM). In contrast, compound **33b**, with a *para*-substituted linker relative to **33a**’s *meta*-position, significantly enhanced PARP1 inhibition (IC_50_: 18 nM) while maintaining comparable ATR potency (IC_50_: 13.40 nM), but PARP7 inhibition decreased (IC_50_: 44 nM). This suggests that *para*-substitution favours PARP1 selectivity.

Compounds **38a** and **38b**, incorporating carbonyl-containing and methylene-containing linkers, demonstrated exceptional selectivity for PARP1 over PARP7. Compound **38a** achieved potent PARP1 inhibition (IC_50_: 5 nM) and ATR activity (IC_50_: 14.70 nM), with negligible PARP7 inhibition (IC_50_ > 1000 nM). Similarly, **38b** showed strong PARP1 potency (IC_50_: 8.50 nM) and moderate ATR activity (IC_50_: 27.70 nM), also with no significant PARP7 inhibition (IC_50_ > 1000 nM). These results highlight the carbonyl linker’s role in abolishing PARP7 activity while preserving dual PARP1/ATR inhibition, aligning with the therapeutic goal of targeting breast cancer-relevant pathways without off-target effects.

Compounds **42a**-**42d** and **43** explored ring size and substituent effects on the linker. Compound **42a**, with a carbonyl group, showed moderate PARP1 inhibition (IC_50_: 50 nM), potent ATR activity (IC_50_: 3.95 nM, surpassing AZD6738’s IC_50_: 5.60 nM), and reduced PARP7 potency (IC_50_: 19 nM). Introducing a six-membered nitrogen-containing ring in **42b** markedly improved PARP1 inhibition (IC_50_: 5.40 nM) and ATR potency (IC_50_: 2.94 nM), with decreased PARP7 activity (IC_50_: 48 nM), indicating that the six-membered ring enhances dual PARP1/ATR targeting. Compound **42c**, featuring a five-membered ring, achieved the highest PARP1 potency in the series (IC_50_: 0.97 nM), with good ATR inhibition (IC_50_: 10 nM) but reduced PARP7 activity (IC_50_: 150 nM), suggesting that the five-membered ring optimises PARP1 selectivity. Compound **42d**, with a four-membered ring, showed slightly reduced PARP1 potency (IC_50_: 13 nM) compared to **42c**, improved PARP7 inhibition (IC_50_: 35 nM *vs.* 150 nM of **42c**’s), and strong ATR activity (IC_50_: 4.99 nM). Finally, **43**, with a positional substituent change relative to **42c**, maintained good PARP1 inhibition (IC_50_: 5.80 nM) but exhibited decreased potency against PARP7 (IC_50_: 103 nM) and ATR (IC_50_: 21 nM), underscoring the sensitivity of ATR and PARP7 inhibition to substituent positioning.

In summary, Series II SAR analysis reveals that linker modifications significantly influence selectivity and potency. Carbonyl-containing linkers (**38a**, **38b**) eliminate PARP7 inhibition while maintaining dual PARP1/ATR inhibition activity, ideal for therapeutic applications. Ring size variations in this series demonstrate that five- and six-membered rings enhance PARP1 potency, with the former (**42c**) achieving subnanomolar activity. These findings, combined with Series I data, provide a robust framework for designing dual PARP1/ATR inhibitors, with **38a** and **42c** emerging as lead candidates for further development in breast cancer treatment.

### Antiproliferative activity against TNBC cells

To translate the promising enzyme inhibitory profiles observed in Series I and II into cellular efficacy, all target compounds were evaluated for their antiproliferative effects against two TNBC cell lines: MDA-MB-231 (BRCA1/2 wild-type) and MDA-MB-468 (harboring a BRCA1). Cytotoxicity was assessed *via* CTG assays after 72 h of treatment, with results expressed as IC_50_ values (μM, mean ± SD from triplicate experiments) or percent inhibition at specified concentrations where full dose-response curves could not be determined due to limited solubility, low potency, or ultrapotent activity (Table 3). Lower IC_50_ values indicate stronger inhibition of cell proliferation. Reference compounds included Niraparib, AZD6738, and their combination (1: 1) to benchmark potential synergistic effects.

**Table 3.** Inhibitory activities of all target compounds against MDA-MB-231 and MDA-MB-468 cells.

Compound	IC_50_ (μM)^*a*^	
	MDA-MB-231	MDA-MB-468
**27a**	6.22 ± 1.13	0.85 ± 0.09
**27b**	6.82 ± 0.67	0.78 ± 0.11
**27c**	36.56% @ 30	46.77% @ 30
**27d**	44.96% @ 30	2.91 ± 0.26
**27e**	38.52% @ 30	2.89 ± 0.23
**27f**	48.88% @ 30	2.31 ± 0.54
**27 g**	1.02 ± 0.07	1.20 ± 0.26
**27h**	35.19% @ 30	30.11% @ 30
**27i**	3.29 ± 0.75	4.56 ± 0.08
**27j**	36.90% @ 30	29.44% @ 30
**27k**	43.35% @ 30	45.23% @ 30
**33a**	2.37 ± 0.36	0.67 ± 0.06
**33b**	0.92 ± 0.09	0.66 ± 0.04
**38a**	66.19% @ 0.048	0.01 ± 0.01
**38b**	0.23 ± 0.45	0.18 ± 0.01
**42a**	30.91% @ 30	2.41 ± 0.15
**42b**	14.87 ± 5.63	0.86 ± 0.01
**42c**	28.03% @ 30	2.06 ± 0.40
**42d**	44.1% @ 30	1.08 ± 0.01
**43**	15.52 ± 10.60	1.23 ± 0.30
Niraparib (**3**)	12.47 ± 1.87	43.16% @ 30
AZD6738 (**9**)	14.83 ± 1.60	4.2% @ 30
**3 **+** 9** (1 : 1)	2.19 ± 0.41	3.30 ± 0.06

^a^ Assays were performed in replicate (*n* = 3); IC_50_ values are shown as mean ± SD.

Niraparib alone exhibited moderate activity against MDA-MB-231 (IC_50_: 12.47 ± 1.87 μM) but was largely ineffective against MDA-MB-468 (43.16% inhibition at 30 μM, suggesting IC_50_ > 30 μM). Similarly, AZD6738 showed limited potency in MDA-MB-231 (IC_50_: 14.83 ± 1.60 μM) and negligible effects in MDA-MB-468 (4.2% at 30 μM). However, their equimolar combination demonstrated enhanced antiproliferative activity, with IC_50_ values of 2.19 μM in MDA-MB-231 and 3.30 μM in MDA-MB-468, underscoring the synergistic potential of PARP1/ATR dual inhibition in TNBC cells.

In Series I (**27a**–**27k**), which focused on Region A modifications, cellular activity varied widely, often correlating with enzyme inhibition patterns. Compounds with balanced PARP1/ATR potency, such as **27a** (PARP1 IC_50_: 907 nM; ATR IC_50_: 9 nM), displayed moderate cytotoxicity in MDA-MB-231 (IC_50_: 6.22 ± 1.13 μM) and improved efficacy in MDA-MB-468 (IC_50_: 0.85 ± 0.09 μM). Similarly, **27b** (PARP1 IC_50_: 261 nM; ATR 79.70% at 1000 nM) showed comparable activity (MDA-MB-231 IC_50_: 6.82 ± 0.67 μM; MDA-MB-468 IC_50_: 0.78 ± 0.11 μM). In contrast, compounds with weaker dual inhibition, like **27c** (strong PARP1 but poor ATR) and **27d**–**27f**, exhibited low potency, often achieving 2 μM in MDA-MB-468. Notably, **27 g**, a potent PARP1-selective inhibitor (PARP1 IC_50_ < 10 nM), achieved good cellular activity (MDA-MB-231 IC_50_ = 1.02 ± 0.07 μM; MDA-MB-468 IC_50_: 1.20 ± 0.26 μM), suggesting PARP1 dominance in some contexts. However, compounds with minimal enzyme activity (**27h**–**27k**) showed correspondingly poor cytotoxicity, with < 50% inhibition at 30 μM in both lines. Overall, Series I SAR indicates that morpholine-based substituents (e.g. **27a**, **27b**) support better cellular translation, particularly in MDA-MB-468, while ATR-selective or inactive analogs underperform.

Series II compounds (**33a**–**33b**, **38a**–**38b**, **42a**–**42d, 43**), optimised with linker variations, generally outperformed Series I, reflecting improved dual PARP1/ATR profiles and reduced PARP7 off-target effects. Compound **33a** (PARP1 IC_50_: 101 nM; ATR IC_50_: 14.60 nM) showed enhanced activity over references (MDA-MB-231 IC_50_: 2.37 ± 0.36 μM; MDA-MB-468 IC_50_: 0.67 ± 0.06 μM), with *para*-substitution in **33b** further boosting potency (MDA-MB-231 IC_50_: 0.92 ± 0.09 μM; MDA-MB-468 IC_50_: 0.66 ± 0.04 μM). Carbonyl-linked analogs **38a** and **38b**, with excellent PARP1/ATR selectivity (PARP7 > 1000 nM), emerged as standouts. Compound **38a** displayed ultrapotent activity in MDA-MB-468 (IC_50_: 0.01 ± 0.01 μM) and remarkable inhibition in MDA-MB-231 (66.19% at 0.048 μM, implying IC_50_ < 0.048 μM), surpassing the Niraparib/AZD6738 combination by over 300-fold in MDA-MB-468. Similarly, **38b** achieved submicromolar potency (MDA-MB-231 IC_50_:0.23 ± 0.45 μM; MDA-MB-468 IC_50_: 0.18 ± 0.01 μM). In the **42** and **43** series, ring size influenced outcomes: **42a** (five-membered ring precursor) showed weak MDA-MB-231 activity (30.91% at 30 μM) but moderate in MDA-MB-468 (IC_50_: 2.41 ± 0.15 μM), while six-membered (**42b**) and five-membered (**42c**) variants improved selectivity but not overall potency compared to **38a** (e.g. **42b**: MDA-MB-231 IC_50_: 14.87 ± 5.63 μM; MDA-MB-468 IC_50_: 0.86 ± 0.01 μM). Smaller rings (**42d**) or positional changes (**43**) yielded intermediate results, with IC_50_ values around 1.23–> 30 μM.

**Figure 4.**
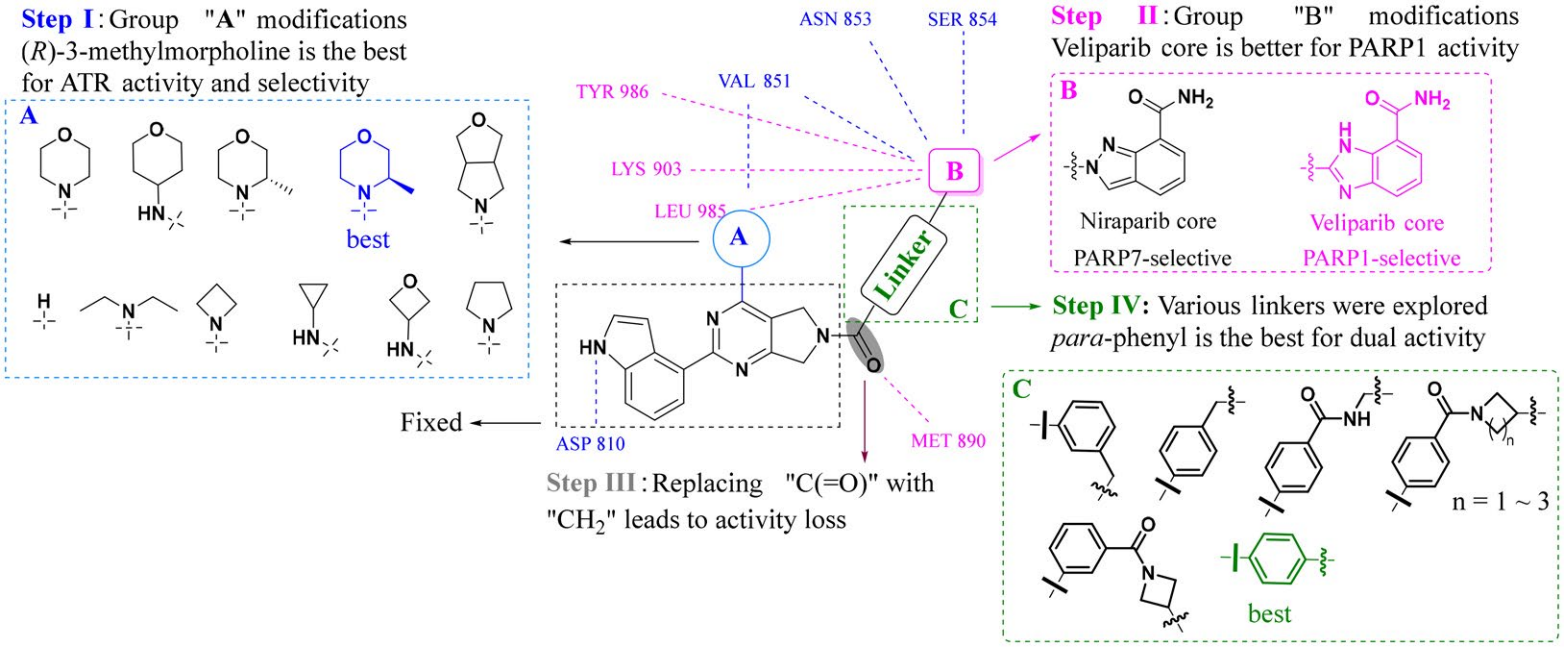
The SAR summary integrated with docking results. Amino acid residues are presented in two different colours: pink from PARP, blue from ATR.

The SAR trend of this work integrated with docking results was highlighted in Figure 4. Guided by the docking experiment, 1*H*-indole motif was fixed due to its nitrogen having hydrogen bond interaction with Asp-810. For the (*R*)-3-methylmorpholine is important for ATR activity and selectivity against other PI3K kinase family. When this group was replaced with other groups, either the ATR activity will lose or ATR selectivity will be worsened. For the PARP pharmacophore portion, we examined two motifs, one from Niraparib, the other from Veliparib. Generally speaking, the derivatives with Niraparib pharmacophore give better PARP1 activity, and selectivity over PARP7 is higher. In contract, the derivatives with Veliparib are more potent against PARP7 than against PARP1. The results could be easily seen in two representative compounds **27a** and **38a**. For the linker portion, we examined various length consisting of majority of para-substituted phenyl group, the effects of the linker on ATR activity and selectivity are minimal. In summary, these data highlight a clear SAR trend: dual PARP1/ATR inhibitors with optimised linkers (e.g. carbonyl in **38a**/**38b**) exhibit superior antiproliferative effects in TNBC cells compared to single-target references or their combination, particularly in BRCA1-mutant MDA-MB-468 where DNA repair vulnerabilities may amplify synergy. The lead compound **38a**, with IC_50_ value less than 50 nM, demonstrates robust *in vitro* efficacy, supporting its advancement for mechanistic studies on cell cycle arrest, apoptosis, and DNA damage repair inhibition, as noted in the abstract. These findings validate the design strategy of merging PARP1 and ATR pharmacophores and provide a foundation for *in vivo* evaluations in breast cancer models.

### Compound 38a induces cell cycle arrest in MDA-MB-231 cells

**Figure 5.**
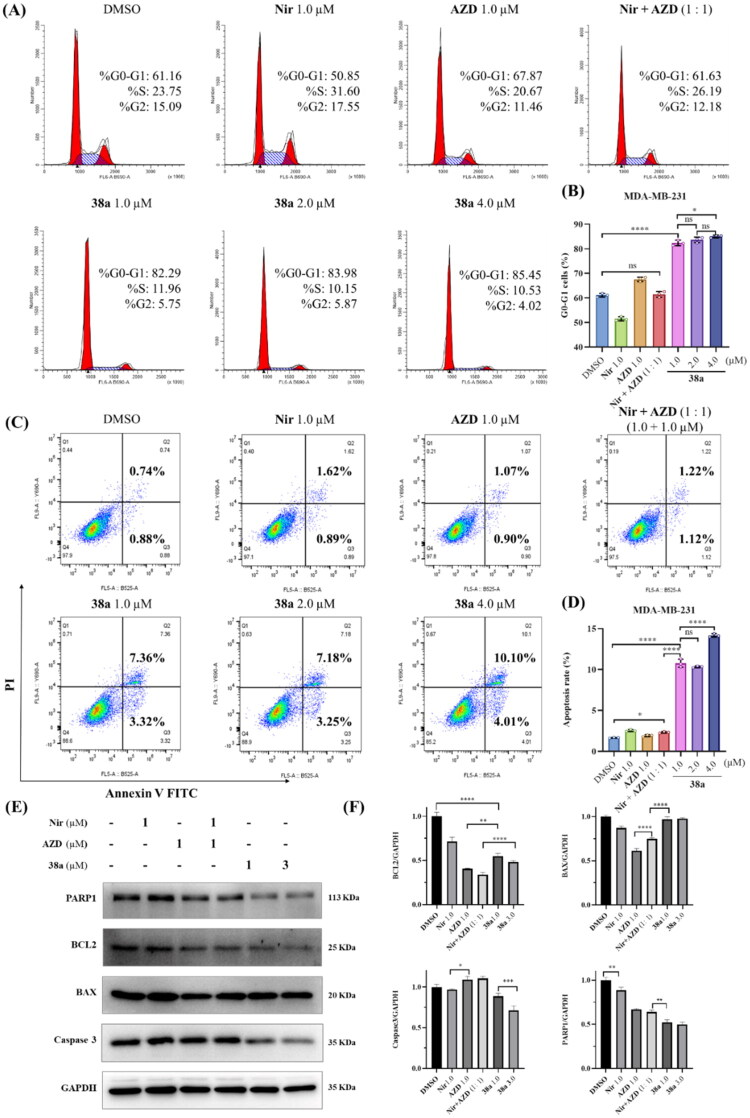
Compound **38a** significantly enhances cell cycle arrest and induces apoptosis. (A) Cell cycle profile and distribution of MDA-MB-231 cells under 48 h treatment with different concentrations of compounds as indicated; (B) Quantitative data of cell cycle distribution were calculated as the mean ± SD of three sets of experiments (*n* = 3); (C) Annexin V-FITC/PI dual staining assay to determine the apoptosis of MDA-MB-231 cells after 48 h of various treatments as indicated; (D) Quantitative data of flow cytometry were calculated as the mean ± SD of three sets of experiments (*n* = 3). (E) Western blotting of PARP1, BCL2, BAX and caspase-3 in MDA-MB-231 cells exposed to different compounds for 48 h; (F) Relative densitometric values of PARP1, BCL2, BAX and caspase-3. Quantitative data were calculated as the mean ± SD of three sets of experiments (*n* = 3). NS: no significance. *p < 0.05, **p < 0.01, ***p < 0.001, ****p < 0.0001.

Given its superior dual PARP1/ATR inhibitory profile and potent antiproliferative activity (Table 3), compound **38a** was selected for mechanistic studies in MDA-MB-231 cells (BRCA1/2 wild-type TNBC). The effects of **38a** on cell cycle progression were evaluated using flow cytometry after 48 h treatment, compared against DMSO (0.01%), Niraparib (1.0 μM), AZD6738 (1.0 μM), their 1:1 combination (1.0 μM each), and **38a** at three concentrations (1.0, 2.0, and 4.0 μM) (Figure 5(A,B)). Quantitative data, reported as mean ± SD from three independent experiments, revealed distinct cell cycle profiles. Niraparib significantly reduced G0/G1-phase cells (50.85% *vs.* 61.16% in DMSO) while increasing S-phase cells (31.60% *vs.* 23.75%), consistent with PARP1 inhibition disrupting DNA repair and stalling replication forks. In contrast, AZD6738 alone or in combination with Niraparib showed minimal impact on cell cycle distribution, suggesting limited cell cycle perturbation under these conditions. However, compound **38a** markedly increased G0/G1-phase cells across all tested concentrations (1.0, 2.0, 4.0 μM), with statistically significant differences compared to control and reference treatments (*p < 0.05 to ****p < 0.0001). Unlike Niraparib’s S-phase arrest, **38a**’s induction of G0/G1 arrest suggests a distinct mechanism, likely driven by simultaneous inhibition against both PARP1 and ATR that halts cell cycle progression earlier, potentially by enhancing DNA damage checkpoints. Notably, no clear dose-dependent effect was observed, indicating that **38a**’s impact on G0/G1 arrest may plateau at lower concentrations, warranting further dose optimisation studies.

### Compound 38a induces cell apoptosis in MDA-MB-231 cells

To investigate the pro-apoptotic effects of **38a**, Annexin V-FITC/PI staining was performed on MDA-MB-231 cells after 48 h treatment with DMSO (0.01%), Niraparib (1.0 μM), AZD6738 (1.0 μM), their combination (1.0 μM each), and **38a** (1.0, 2.0, and 4.0 μM) (Figure 5(C,D)). The results showed that Niraparib, AZD6738, and their combination induced modest apoptosis (1.62–2.51% apoptotic cells) compared to DMSO, with no statistical significance. In contrast, **38a** significantly increased apoptosis, with 10.43% apoptotic cells at 1.0 μM, rising to 10.68% at 2.0 μM and 14.11% at 4.0 μM (*p < 0.05 to ****p < 0.0001 *vs.* other groups), demonstrating a dose-dependent enhancement. To elucidate the underlying mechanism, Western blot analysis was conducted to assess apoptosis-related proteins (Figure 5(E,F)). Compound **38a** significantly reduced expression of the anti-apoptotic protein B-cell lymphoma-2 (BCL2) and caspase-3 levels, while markedly increasing pro-apoptotic BCL2 associated X protein (BAX) levels compared to Niraparib, AZD6738, or their combination (*p < 0.05 to ****p < 0.0001). These findings indicate that **38a**’s dual PARP1/ATR inhibition synergistically amplifies apoptotic signalling, likely by exacerbating DNA damage and impairing repair pathways, surpassing the efficacy of single-target inhibitors or their combination.

### Compound 38a inhibits colony formation and migration in MDA-MB-231 cells

**Figure 6.**
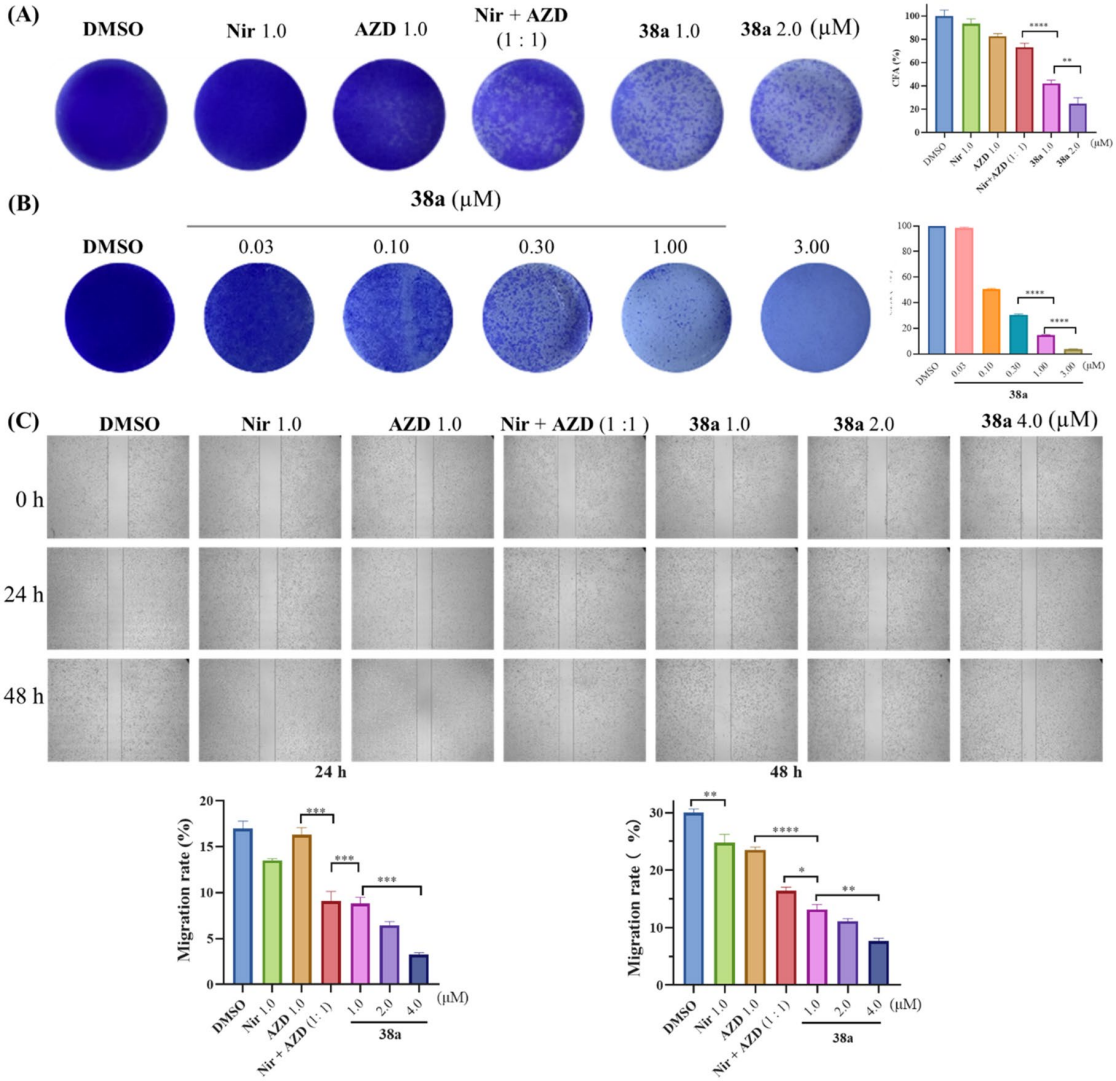
Compound **38a** significantly inhibits colony formation and migration ability of MDA-MB-231 cells. (A) Colony formation capacity of MDA-MB-231 cells after treatment with different compounds for 10 days. (B) Colony formation assay (CFA) of MDA-MB-231 cells after treatment with different concentrations of **38a** for 10 days. (C) Representative images of wound-healing assay and the percentage of wound-healed distance after treatment with different compounds for 0 h, 24 h and 48 h in MDA-MB-231 cells. Quantitative data were calculated as the mean ± SD of three sets of experiments (*n* = 3). *p < 0.05, **p < 0.01, ***p < 0.001, ****p < 0.0001, ns: no significance.

Following the observation that compound **38a** potently induces G0/G1 cell cycle arrest and apoptosis in MDA-MB-231 cells, its effects on clonogenicity and migratory capacity were investigated to further elucidate its anti-tumour potential in TNBC. Colony formation assays were conducted to assess the ability of **38a** to inhibit long-term proliferative potential compared to DMSO (0.01%), Niraparib (1.0 μM), AZD6738 (1.0 μM), and their 1: 1 combination (1.0 μM each). At 1.0 μM, **38a** significantly reduced colony formation in MDA-MB-231 cells compared to Niraparib, AZD6738, or their combination (*p < 0.05 to ****p < 0.0001), as visualised in Figure 6(A). To explore dose dependency, **38a** was tested at multiple concentrations (0.03, 0.1, 0.3, 1.0, 3.0 μM), revealing a clear concentration-dependent inhibition of colony formation, with higher doses yielding greater suppression (Figure 6(B),**p < 0.01 to ****p < 0.0001). This suggests that **38a**’s dual PARP1/ATR inhibition effectively disrupts the clonogenic survival of TNBC cells, surpassing the efficacy of single-target inhibitors or their combination.

To evaluate the impact on metastatic potential, wound-healing assays were performed to measure MDA-MB-231 cell migration after 24 h and 48 h treatment with the same conditions (Figure 6(C,D)). These results demonstrated that **38a** (1.0 μM) significantly inhibited cell migration compared to DMSO, Niraparib, AZD6738, and their combination (*p < 0.05 to ****p < 0.0001). The superior anti-migratory effect of **38a** suggests that its dual inhibition of PARP1 and ATR disrupts critical pathways for cytoskeletal dynamics and cell motility, likely through enhanced DNA damage and impaired repair signalling. These results highlight **38a**’s multifaceted anti-tumour activity, effectively targeting both proliferative and metastatic properties of MDA-MB-231 cells.

### Compound 38a Inhibits DNA damage repair in MDA-MB-231 cells

**Figure 7.**
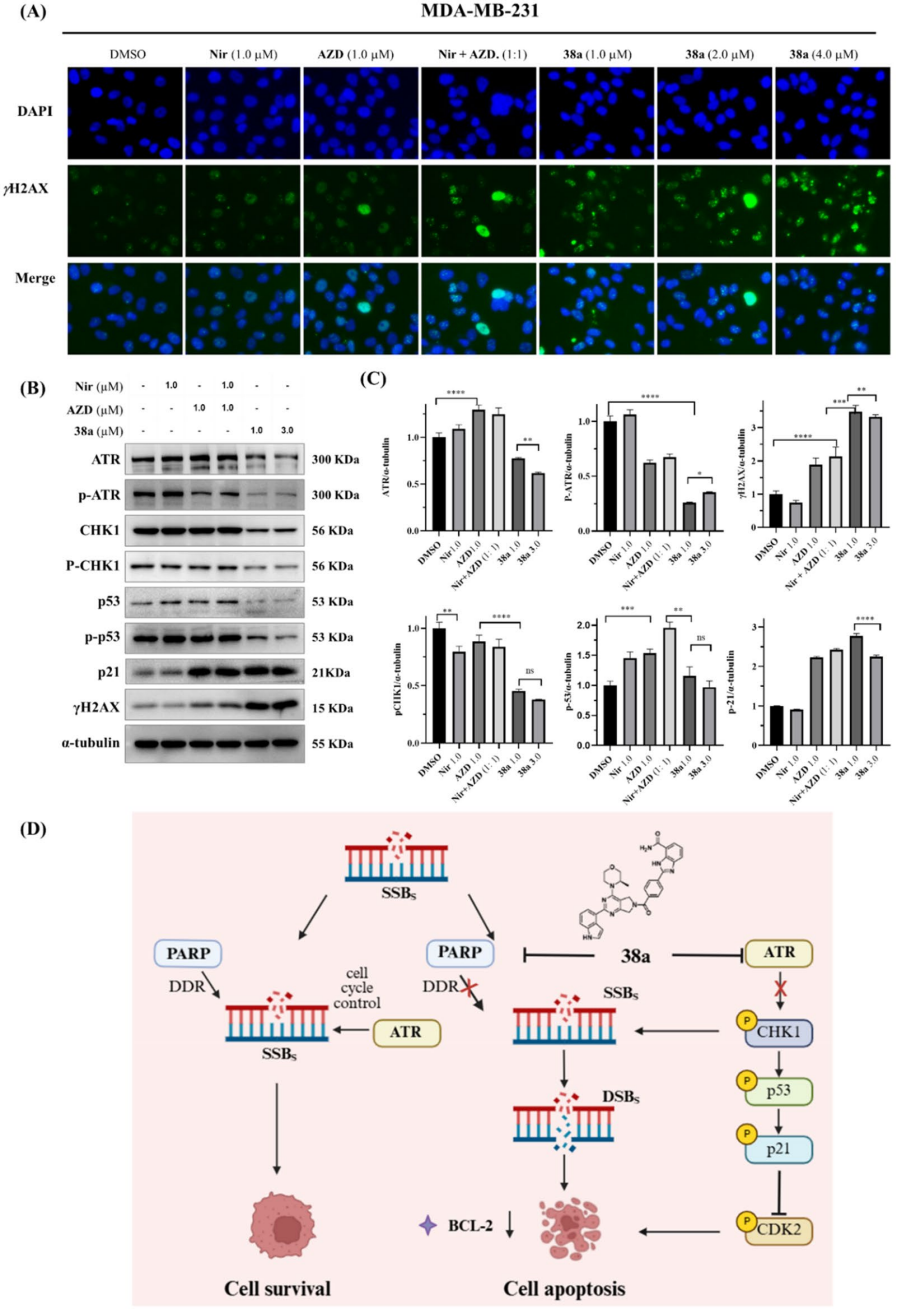
Compound **38a** significantly inhibits DSB repair and ATR-CHK1 signalling axis. (A) Representative images of immune-fluorescence staining of γH2AX foci in MDA-MB-231 cells treated with compounds as indicated for 24 h. Scale bar = 100 μm; (B) Western blotting of ATR/p-ATR, CHK1/p-CHK1, p53/p-p53, p21 and γH2AX in MDA-MB-231 cells exposed to different compounds as indicated for 48 h; (C) Statistical analysis of ATR/p-ATR, γH2AX, p-CHK1, p-p53 and p21 protein levels; (D) Mechanism of action of **38a** in the treatment of TNBC. The data are shown as the mean ± SD of three independent experiments. NS: no significance. *p < 0.05, **p < 0.01, ***p < 0.001, ****p < 0.0001.

To elucidate the mechanistic basis of compound **38a**’s potent anti-tumour effects in TNBC, we investigated its impact on DNA damage and the ATR-CHK1 signalling axis, which are critical pathways targeted by PARP1 and ATR inhibitors. Immunofluorescence staining was performed to assess nuclear γH2AX foci, a hallmark of DNA damage, in MDA-MB-231 cells treated for 24 h with DMSO (0.01%), Niraparib (1.0 μM), AZD6738 (1.0 μM), their 1:1 combination (1.0 μM each), or **38a** (1.0 , 2.0, 4.0 μM). Compared to DMSO group and combination group, **38a** significantly increased γH2AX foci formation, indicating pronounced impairment of DNA damage repair efficiency in TNBC cells (Figure 7(A)).

To further validate these findings, Western blot analysis was conducted after 48 h treatment to quantify γH2AX and key proteins in the ATR-CHK1 pathway (Figure 7(B,C)). These results confirmed that **38a** significantly upregulated γH2AX levels compared to Niraparib, AZD6738, or their combination (*p < 0.05 to ****p < 0.0001), consistent with enhanced DNA damage accumulation. Additionally, **38a** markedly reduced phosphorylation of ATR (p-ATR) and its downstream effector CHK1 (p-CHK1), reflecting potent ATR inhibition. This was accompanied by significant downregulation of phosphorylated p53 (p-p53) and p21, critical regulators of cell cycle progression and DNA damage response (*p < 0.05 to ****p < 0.0001), aligning with **38a**’s induction of G0/G1 arrest (Section 2.6) (Figure 7(D)). In contrast, Niraparib and AZD6738, alone or combined, showed modest effects on these markers, underscoring **38a**’s superior efficacy.

Collectively, these results demonstrate that **38a**’s dual PARP1/ATR inhibition synergistically disrupts DNA damage repair and suppresses the ATR-CHK1-p53-p21 axis, leading to accumulated DNA damage, cell cycle arrest, and apoptosis in MDA-MB-231 cells.

### Kinase selectivity profile of 38a

To assess the specificity of compound **38a** as a dual PARP1/ATR inhibitor, its selectivity was evaluated against a panel of related enzymes, including PARP1, PARP2, PARP7, ATR, mTOR, ATM, PI3Kα, and DNA-PK, with results compared to reference inhibitors Niraparib (PARP1 inhibitor) and AZD6738 (ATR inhibitor) (Tables 4 and 5). Assays were conducted in triplicate, with IC_50_ values reported as mean ± SD. For PARP isoforms, **38a** exhibited potent inhibition of PARP1 (IC_50_: 5.00 nM), comparable to Niraparib (IC_50_: 8.2 nM), and strong activity against PARP2 (IC_50_: 2.5 nM), but showed no significant inhibition of PARP7 (IC_50_ > 1000 nM), indicating high selectivity for PARP1/2 over PARP7. For ATR isoforms, **38a** demonstrated robust ATR inhibition (IC_50_: 14.70 nM), approaching AZD6738’s potency (IC_50_: 5.60 nM), with moderate activity against mTOR (IC_50_: 60.16 nM) but negligible effects on ATM, PI3Kα, and DNA-PK (IC_50_ > 10,000 nM, > 10,000 nM, and 5896 nM, respectively), compared to AZD6738’s broader off-target profile (e.g. mTOR IC_50_: 1075.80 nM, ATM IC_50_: 2493 nM). These data confirm **38a**’s favourable selectivity for PARP1/2 and ATR, with minimal off-target activity, supporting its potential as a targeted therapeutic for TNBC with reduced risk of non-specific kinase inhibition.

**Table 4.** PARP1 selectivity profiles of **38a** and Niraparib*a.*

Compd.	IC_50_ (nM)		
	PARP1	PARP2	PARP7
**38a**	5.00 ± 2.93	2.5	> 1000
**3** (Niraparib)	8.20 ± 1.37	2.9	No determined

^a^ Assays were performed in replicate (*n* = 3); IC_50_ values are shown as mean ± SD.

**Table 5.** ATR selectivity profiles of **38a** and AZD6738*a.*

Compd.	IC_50_ (nM)				
	ATR	mTOR	ATM	PI3Kα	DNA-PK
**38a**	14.70 ± 2.01	60.16	> 10000	> 10000	5896
**9** (AZD6738)	5.60 ± 0.23	1075.80	2493	17541	4708

^a^ Assays were performed in replicate (*n* = 3); IC_50_ values are shown as mean ± SD.

### Molecular docking of 38a with PARP1 and ATR

**Figure 8.**
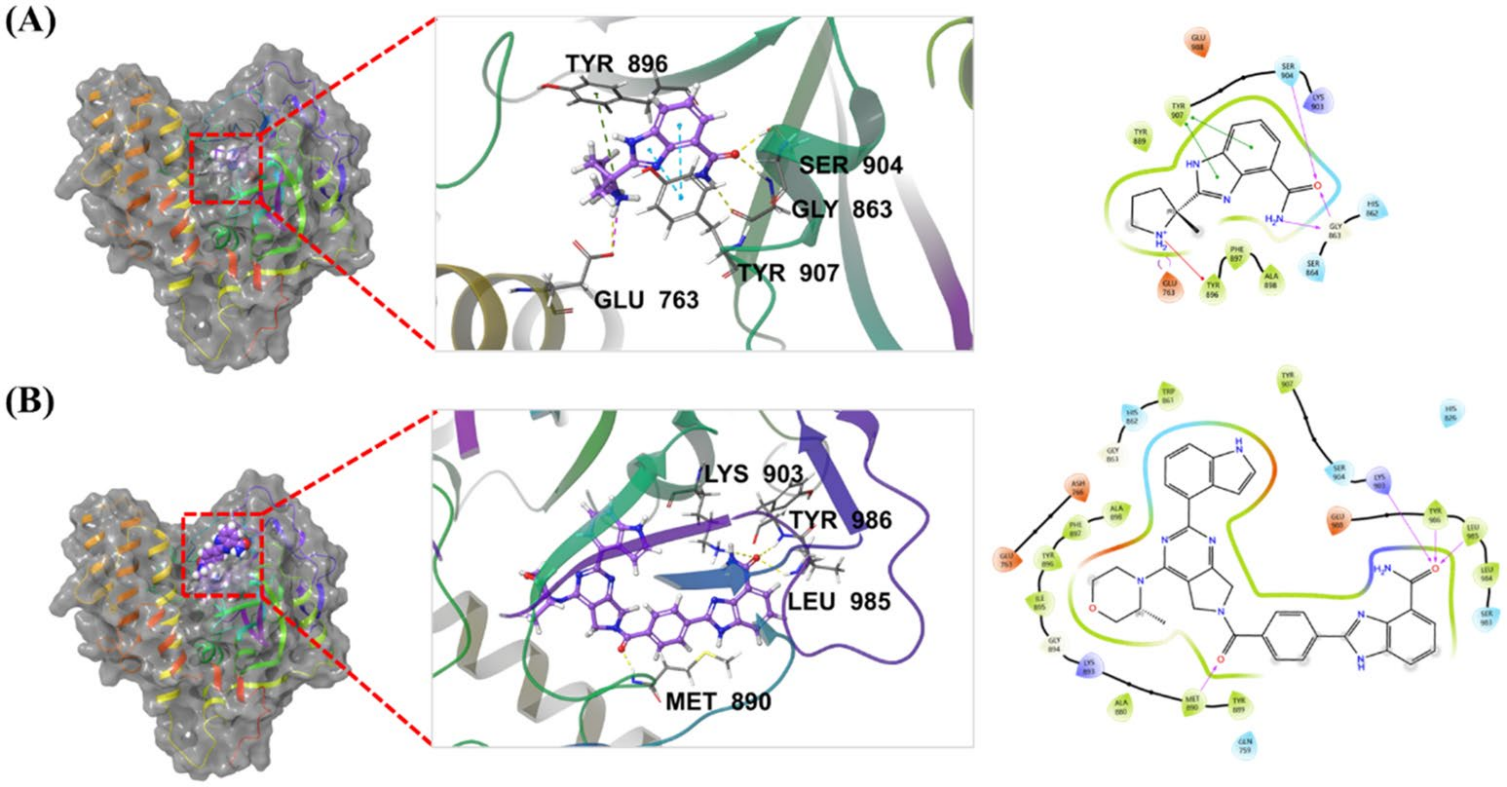
Molecular docking and binding modes of compounds with PARP1. (A) Compound Veliparib docking with PARP1 co-crystal structures (PDB: 7KK6) and the 3D and 2D views of the key residues from the protein with Veliparib; (B) Compound **38a** docking with PARP1 co-crystal structures (PDB: 7KK6) and the 3D and 2D view of the key residues from the protein with **38a**. Hydrogen bonds are indicated by yellow dotted lines, and π-π interactions are marked by blue dotted lines (3D).

**Figure 9.**
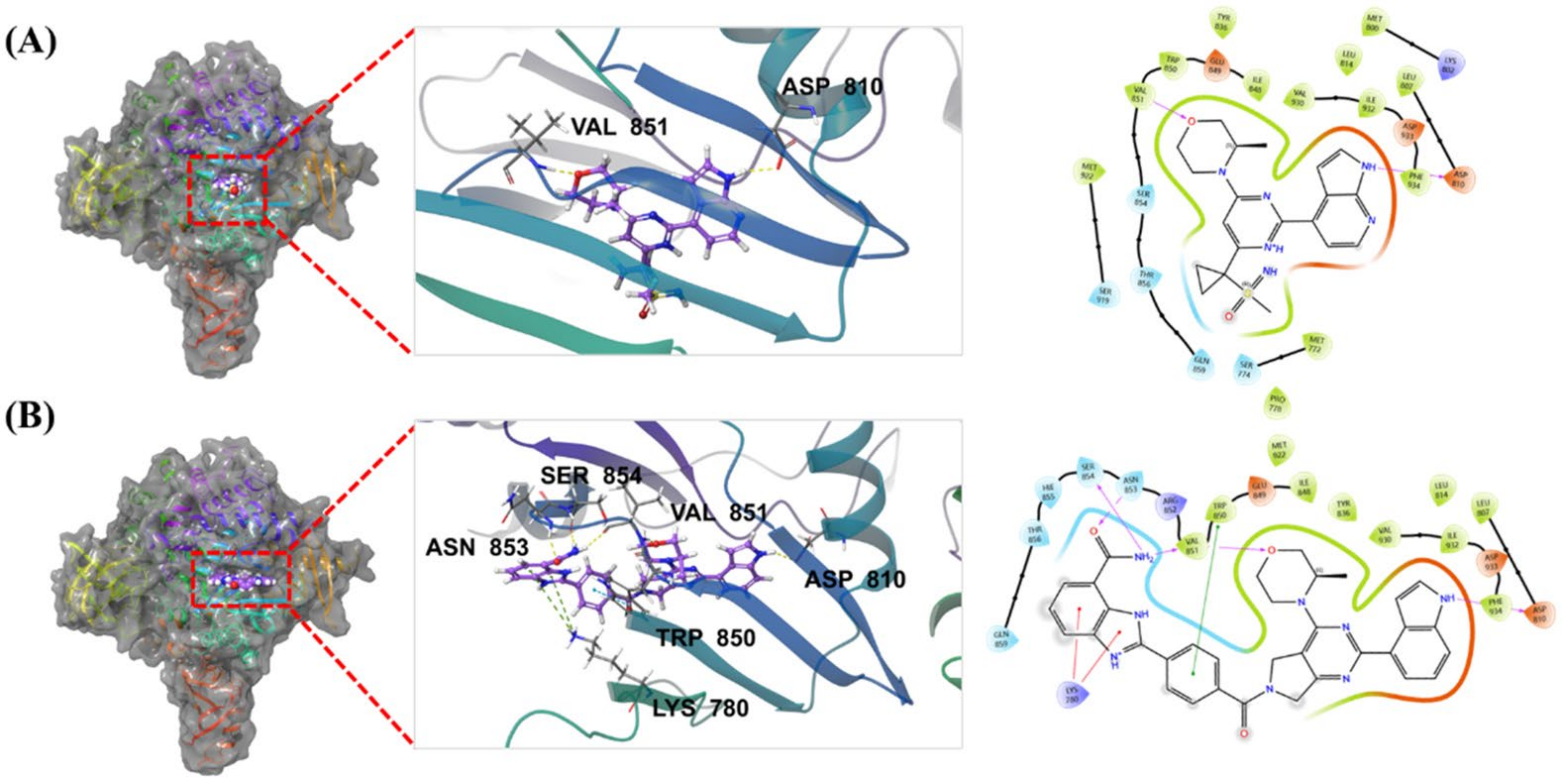
Molecular docking and binding modes of compounds with ATR. (A) Compound AZD6738 docking using PI3Kα mutant (a mimetic ATR protein, PDB ID: 5UK8) and the 3D and 2D views of the key residues from the protein with AZD6738; (B) Compound **38a** docking using PI3Kα mutant (a mimetic ATR protein, PDB ID: 5UK8) and the 3D and 2D view of the key residues from the protein with **38a**. Hydrogen bonds are indicated by yellow dotted lines, and π-π interactions are marked by blue dotted lines (3D).

To elucidate the molecular basis of compound **38a**’s dual PARP1/ATR inhibitory activity, molecular docking studies were conducted using the co-crystal structure of PARP1 complexed with Veliparib (PDB: 7KK6) and a PI3Kα mutant as a surrogate for ATR (PDB: 5UK8) (Figures 8 and 9).^30–33^ Docking simulations revealed the key interactions driving **38a**’s binding affinity and specificity for both targets, with hydrogen bonds depicted as yellow dotted lines and π-π interactions as blue dotted lines.

For PARP1, 1*H*-benzo[*d*]imidazole-7-carboxamide motif from **38a** forms multiple critical hydrogen bonds with key residues, including Gly863, Ser904, and Arg878, mirroring the binding mode of Veliparib (Figure 8(A,B)). Additionally, the pyrrolo[3,4-*d*]pyrimidine core of **38a** engages in π-π stacking with Tyr907, enhancing binding stability. These interactions anchor **38a** within the PARP1 active site, contributing to its potent inhibition (IC_50_: 5.00 nM, Table 4).

When we docked **38a** with PI3Kα mutant structure (mimetic for ATR protein), we found that the compound forms hydrogen bonds with Val851 and Asp810, consistent with AZD6738’s binding mode (Figure 9(A,B)). In addition, **38a** would possibly forms additional three hydrogen bonds with Val851, Ser854, and Asn853. Meanwhile its linker region engages in π-π stacking with Trp850. These additional interactions, absent in AZD6738, likely enhance **38a**’s binding affinity and contribute to its ATR potency (IC_50_: 14.70 nM *vs.* AZD6738’s 5.60 nM, Table 5) and superior cellular activity in TNBC cells (Table 3). Collectively, these docking results highlight the critical interactions between **38a** functional groups and both ATR and PARP1 protein residues, confirming the dual inhibition characteristic of this compounds against both PARP1 and ATR in molecular levels. They could also serve as useful resource for design new PARP1/ATR dual inhibitors.

## Conclusion

This study successfully developed a novel series of 6,7-dihydro-5*H*-pyrrolo[3,4-*d*]pyrimidine-based PARP1/ATR dual inhibitors to address the therapeutic challenges of TNBC, particularly in BRCA1/2 wild-type contexts. Lead compound **38a** exhibited exceptional potency against PARP1 (IC_50_: 5.00 nM, better than analog bearing Niraparib core) and good potency against ATR (IC_50_: 14.70 nM, comparable to that of AZD6738). It showed remarkable antiproliferative activity in TNBC cell lines (IC_50_ < 0.048 μM for MDA-MB-231 and 0.01 μM for MDA-MB-468), surpassing the efficacy of Niraparib, AZD6738, and their combination. Mechanistic investigations demonstrated that **38a** induces G0/G1 cell cycle arrest. Unlike literature results reported previously about S phase suppression upon treatment,^19^^,^^22^^,^^34^ this might be due to the capability of **38a** inhibiting other PARP subtypes. Notably, **38a** significantly promotes apoptosis, inhibits colony formation and cell migration in MDA-MB-231 cells. Furthermore, **38a** markedly increased γH2AX levels, indicating robust disruption of DNA damage repair, while downregulating the ATR-CHK1-p53-p21 axis, as confirmed by immunofluorescence and Western blot analyses. These results demonstrate that our compound could inhibit the ATR-CHK1 signalling pathway and the p53-p21 pathway simultaneously, thereby causing changes in the cell apoptosis and DNA repair response. Molecular docking revealed key hydrogen bond and π-π interactions stabilising **38a**’s binding to PARP1 and ATR, supporting it’s dual targeting characteristic in molecular levels. These findings highlight **38a**’s potential as a single molecular entity capable of targeting two pathways for treating TNBC, which might overcome the limitations of single-target PARPi or PARPi and ATRi combination.

## Experimental section

### Chemistry

Unless otherwise stated, all reagents and solvents were purchased from commercial sources. The reaction was monitored by thin layer chromatography (TLC) on 0.25 mm silica gel plate (GF254) and observed under UV light. The melting points of the compounds were determined using Buchi B-540 capillary melting point instrument. ^1^H NMR (500 MHz) and ^13^C NMR (126 MHz) were recorded on a 500 MHz or 400 MHz Bruker NMR spectroscopy using CDCl_3_ and DMSO-*d_6_* as the deuterated solvent. Low resolution mass spectra were recorded with Agilent 1260 Infinity II/6125. High resolution mass spectra (HRMS) were measured on a Bruker MicrOTOF-Q II instrument or Shimadzu LCMS-IT-TOF mass spectrometer using the ESI technique. Column chromatography was performed on silica gel (200–300 mesh).

### General procedure for the synthesis of compound 27a-27k

#### Tert-butyl(R)-2-chloro-4–(3-methylmorpholino)-5,7-dihydro-6H-pyrrolo[3,4-d]pyrimidine-6-carboxyl-ate (18a)

To a solution of compound **16** (250 mg, 0.86 mmol) and **17a** (95.87 mg, 0.95 mmol) in CH_2_Cl_2_ (5 ml), TEA (435.62 mg, 4.31 mmol) was added. The reaction mixture was stirred at 50 °C for 2 h, and the progress of the reaction was monitored by TLC. Upon completion, the mixture was quenched with water (50 ml) at room temperature and extracted with EtOAc (2 × 50 ml). The combined organic layers were washed with brine, dried (Na_2_SO_4_), and concentrated under reduced pressure. The crude product was purified *via* column chromatography (petroleum ether/EtOAc = 4: 1, v/v) to afford intermediate **18a** (231 mg, yield 75%) as a white solid.^1^H NMR (500 MHz, DMSO-*d*_6_) δ 4.79 − 4.63 (m, 2H), 4.36 (dt, *J* = 13.2, 2.5 Hz, 2H), 3.91 (dd, *J* = 11.9, 3.4 Hz, 2H), 3.65 (ddd, *J* = 49.6, 11.7, 3.8 Hz, 2H), 3.44 (td, *J* = 11.8, 2.8 Hz, 1H), 3.30 (s, 2H),1.45 (d, *J* = 3.0 Hz, 9H), 1.24 (dd, *J* = 6.8, 2.0 Hz, 3H).

#### Tert-butyl (R)-2-(1H-indol-4-yl)-4–(3-methylmorpholino)-5,7-dihydro-6H-pyrrolo [3,4-d]pyrimidine-6-carboxylate (20a)

To a solution of compound **18a** (231 mg, 0.65 mmol) and compound **19** (237.96 mg, 0.98 mmol) in 1,4-dioxane (6 ml) and H_2_O (2 ml), K_3_PO_4_ (276.87 mg, 1.31 mmol) was added. The reaction system was flushed with nitrogen for a 10-min duration to displace air, Pd(PPh_3_)_4_ (75.46 mg, 0.07 mmol) was added and the reaction system was purged one more time with nitrogen gas. The reaction mixture was stirred at 100 °C for 6 h. The reaction mixture was cooled to room temperature the solvent was removed under reduced pressure. The mixture was quenched with water (50 ml) and extracted with EtOAc (2 × 50 ml). Combined organic layers were washed with brine, dried (Na_2_SO_4_), and concentrated under reduced pressure. The solvent was removed under reduced pressure. The residue was purified *via* silica column chromatography to give compound **20a** (235 mg, yield 82.7%). ^1^H NMR (500 MHz, DMSO-*d*_6_) δ 11.23 (s, 1H), 8.04 (d, *J* = 7.5 Hz, 1H), 7.57 − 7.50 (m, 1H), 7.43 (q, *J* = 2.7 Hz, 1H), 7.30 (q, *J* = 2.7 Hz, 1H), 7.17 (t, *J* = 7.8 Hz, 1H), 5.76 (s, 1H), 4.90 – 4.70 (m, 2H), 4.59 – 4.36 (m, 3H), 4.18 – 3.96 (m, 2H), 3.73 (ddd, *J* = 40.3, 11.5, 4.3 Hz, 2H), 3.54 (td, *J* = 11.7, 2.8 Hz, 1H), 3.42 (td, *J* = 12.1, 8.8 Hz, 1H), 1.49 (s, 9H), 1.30 (d, *J* = 6.8 Hz, 3H).

#### (R)-4–(2-(1H-indol-4-yl)-6,7-dihydro-5H-pyrrolo[3,4-d]pyrimidin-4-yl)-3-methy-lmorpholine (21a)

To a solution of intermediate **20a** (235 mg, 0.54 mmol) in methanol (6 ml) at room temperature was added HCl dioxane solution (2 M, 3 ml). After stirring at room temperature environment for 6 h, the mixture was concentrated under reduced pressure to yield crude product **21a** (230 mg, yield 97.8%) as a yellow solid. The crude was used directly in the next step of reaction.

#### 1H-indazole-7-carboxamide (23)

To a solution of compound **22** (4000 mg, 24.69 mmol) in THF (20 ml) the temperature was lowered to 0 °C, and added SOCl_2_ (29.38 g, 246.9 mmol). The reaction mixture was stirred for 2 h. Then the temperature was further lowered to −10 °C, and NH_3_·H_2_O was slowly dropped in. After adding THF (5 ml) the reaction was conducted for another 2 h under an ice bath. After completion, THF was evaporated off and then recrystallized to give compound **23** (2000 mg, yield 50.3%) as a white solid.^1^H NMR (500 MHz, DMSO-*d*_6_) δ 12.98 (s, 1H), 8.28 – 8.01 (m, 2H), 7.93 (ddd, *J* = 23.8, 7.6, 0.9 Hz, 2H), 7.48 (s, 1H), 7.33 – 7.08 (m, 1H).

#### Methyl 3-((7-carbamoyl-2H-indazol-2-yl)methyl)benzoate (25)

To a solution of compound **23** (2000 mg, 12.41 mmol) and **24** (3127 mg, 13.65 mmol) in CH_2_Cl_2_ (30 ml) was added K_2_CO_3_ (5145.56 mg, 37.23 mmol). After the reagent had been added, the mixture was stirred continuously at 90 °C for 2 h, while the reaction’s progression was monitored by TLC. The mixture was quenched with water (100 ml) at room temperature and extracted with EtOAc (2 × 100 ml). Combined organic layers were washed with brine, dried (Na_2_SO_4_), and concentrated under reduced pressure. The crude was purified *via* column chromatography (petroleum ether/EtOAc = 4: 1, v/v) to give intermediate **25** (2632 mg, yield 68.6%) as a white solid.^1^H NMR (500 MHz, DMSO-*d*_6_) δ 8.77 (s, 1H), 8.50 (d, *J* = 3.1 Hz, 1H), 8.03 − 7.96 (m, 3H), 7.91 (dt, *J* = 7.8, 1.5 Hz, 1H), 7.79 (d, *J* = 3.1 Hz, 1H), 7.66 (dt, *J* = 7.7, 1.5 Hz, 1H), 7.54 (t, *J* = 7.7 Hz, 1H), 7.20 (dd, *J* = 8.3, 7.0 Hz, 1H), 5.84 (s, 2H), 3.83 (s, 3H).

#### 3-((7-Carbamoyl-2H-indazol-2-yl)methyl)benzoic acid (26)

To a solution of compound **25** (2632 mg, 8.51 mmol) in THF (40 ml) was added LiOH (1 N, 20 ml). The reaction mixture was stirred for 4 h. The pH was adjusted to 4 using aqueous HCl solution (1 N) and a white solid was precipitated, the filter cake was filtered, washed with water (5 ml) and the filtrate was collected. The precipitate was collected by filtration and then recrystallized to give compound **26** (2430 mg, yield 92.3%) as a white solid.

#### (R)-2–(3-(2-(1H-indol-4-yl)-4–(3-methylmorpholino)-6,7-dihydro-5H-pyrrolo[3,4 -d]pyrimidine-6-carbonyl)benzyl)-2H-indazole-7-carboxamide (27a)

HOBT (108.91 mg, 0.81 mmol), EDCI (309.02 mg, 1.61 mmol), DIPEA (320.91 mg, 2.48 mmol), and **26** (182.88 mg, 0.62 mmol) were sequentially added to a solution of compound **21a** (230 mg, 0.62 mmol) in DMF (3 ml). The reaction mixture was stirred at room temperature for 4 h. The solvent was removed under reduced pressure. The mixture was quenched with water (50 ml) and extracted with EtOAc (2 × 50 ml). Combined organic layers were washed with brine, dried (Na_2_SO_4_). After filtering, the solvent was removed under reduced pressure. The crude was purified *via* column chromatography (CH_2_Cl_2_/MeOH = 10: 1, v/v) to give compound **27a** (45 mg, yield 10.6%) as a white solid. M.p. 257.7 − 259.8 °C. ^1^H NMR (500 MHz, DMSO-*d*_6_) δ 11.23 (s, 1H), 8.79 − 8.74 (m, 1H), 8.56 (t, *J* = 3.8 Hz, 1H), 8.07 − 7.95 (m, 3H), 7.84 − 7.61 (m, 3H), 7.60 − 7.48 (m, 3H), 7.43 (dt, *J* = 13.9, 2.9 Hz, 1H), 7.31 − 7.13 (m, 3H), 5.90 − 5.74 (m, 2H), 5.11 − 4.92 (m, 2H), 4.73 (d, *J* = 11.6 Hz, 2H), 4.24 − 3.95 (m, 1H), 3.83 – 3.67 (m, 2H), 3.65 – 3.51 (m, 2H), 3.49 – 3.38 (m, 1H), 1.40 − 1.29 (m, 3H), 1.18 (d, *J* = 6.7 Hz, 1H).

#### Tert-butyl 2-chloro-4-morpholino-5,7-dihydro-6H-pyrrolo[3,4-d]pyrimidine-6-carboxylate (18b)

To a solution of compound **16** (200.0 mg, 0.69 mmol) and **17b** (85.0 mg, 0.69 mmol) in CH_2_Cl_2_ (5 ml) was added TEA (348.6 mg, 3.45 mmol). After addition, the mixture was stirred at 50 °C for 2 h. The reaction was monitored by TLC. The mixture was quenched with water (100 ml) at room temperature and extracted with EtOAc (2 × 100 ml). Combined organic layers were washed with brine, dried (Na_2_SO_4_), and concentrated under reduced pressure. The residue was purified *via* column chromatography (petroleum ether/EtOAc = 4: 1, v/v) to give intermediate **18b** (145.0 mg, yield 61.9%) as a white solid. ^1^H NMR (500 MHz, DMSO-*d*_6_) δ 4.71 (q, *J* = 2.8 Hz, 2H), 4.36 (dt, *J* = 13.2, 2.5 Hz, 2H), 3.66 (d, *J* = 5.4 Hz, 4H), 3.61 (q, *J* = 5.4, 5.0 Hz, 4H), 1.45 (d, *J* = 3.4 Hz, 9H).

#### Tert-butyl 2-(1H-indol-4-yl)-4-morpholino-5,7-dihydro-6H-pyrrolo[3,4-d]pyrimi-dine-6-carboxylate (20b)

To a solution of compound **18b** (125.0 mg, 0.37 mmol) and compound **19** (134.0 mg, 0.55 mmol) in 1,4-dioxane (6 ml) and H_2_O (2 ml) was added K_3_PO_4_ (156.0 mg, 0.74 mmol). The reaction system was flushed with nitrogen for a 10-min duration to displace air, Pd(PPh_3_)_4_ (42.53 mg, 0.04 mmol) was added and the reaction system was purged one more time with nitrogen gas. The reaction mixture was stirred at 100 °C for 6 h. The reaction mixture was cooled to room temperature the solvent was removed under reduced pressure. The mixture was quenched with water (50 ml) and extracted with EtOAc (2 × 50 ml). Combined organic layers were washed with brine, dried (Na_2_SO_4_), and concentrated in vacuum. The residue was purified under reduced pressure. The residue was purified *via* silica column chromatography to give compound **20b** (153.0 mg, yield 95%). ^1^H NMR (500 MHz, DMSO-*d*_6_) δ 11.23 (s, 1H), 8.04 (d, *J* = 7.3 Hz, 1H), 7.52 (dt, *J* = 7.9, 1.0 Hz, 1H), 7.43 (q, *J* = 2.6 Hz, 1H), 7.29 (q, *J* = 2.5 Hz, 1H), 7.17 (t, *J* = 7.8 Hz, 1H), 4.79 (dd, *J* = 4.8, 2.4 Hz, 2H), 4.55 − 4.44 (m, 2H), 3.74 (s, 8H), 1.48 (s, 9H).

#### 4–(2-(1H-indol-4-yl)-6,7-dihydro-5H-pyrrolo[3,4-d]pyrimidin-4-yl)morpholine (21b)

To a solution of intermediate **20b** (153.0 mg, 0.36 mmol) in methanol (6 ml) at room temperature was added HCl dioxane solution (2 M, 3 ml). After stirring at room temperature environment for 6 h, the mixture was concentrated under reduced pressure to yield crude product **21b** (150.0 mg, yield 98.0%) as a yellow solid. The crude was used directly in the next step of reaction.

#### 2–(3-(2-(1H-indol-4-yl)-4-morpholino-6,7-dihydro-5H-pyrrolo[3,4-d]pyrimidine-6-carbonyl)benzyl)-2H-indazole-7-carboxamide (27b)

HOBT (71 mg, 0.53 mmol), EDCI (201.4 mg, 1.05 mmol), DIPEA (209.1 mg, 1.61 mmol), and **26** (119 mg, 0.40 mmol) were sequentially added to a solution of compound **21b** (150 mg, 0.40 mmol) in DMF (3 ml). The reaction mixture was stirred at room temperature for 4 h. After completion, the solvent was removed under reduced pressure. The mixture was quenched with water (50 ml) and extracted with EtOAc (2 × 50 ml). Combined organic layers were washed with brine, dried (Na_2_SO_4_). After filtering, the solvent was removed under reduced pressure. The crude was purified *via* column chromatography (CH_2_Cl_2_/MeOH = 10: 1, v/v) to give compound **27b** (67.0 mg, yield 29%) as a white solid. M.p. 236.4 − 238.9 °C.^1^H NMR (500 MHz, DMSO-*d_6_*) d 11.41 − 11.19 (m, 1H), 8.78 (s, 1H), 8.56 (s, 1H), 8.09 − 7.96 (m, 3H), 7.84 − 7.77 (m, 1H), 7.72 (d, *J* = 11.4 Hz, 1H), 7.67 − 7.62 (m, 1H), 7.60 − 7.46 (m, 4H), 7.43 (dt, *J* = 14.2, 2.7 Hz, 1H), 7.37 − 7.08 (m, 3H), 5.83 (d, *J* = 12.0 Hz, 2H), 5.12 − 4.66 (m, 4H), 3.78 (d, *J* = 6.6 Hz, 4H), 3.66 − 3.51 (m, 2H).

#### Tert-butyl 2-chloro-4-((tetrahydro-2H-pyran-4-yl)amino)-5,7-dihydro-6H-pyrrol o[3,4-d]pyrimidine-6-carboxylate (18c)

To a solution of compound **16** (250.0 mg, 0.86 mmol) and **17c** (118.5 mg, 0.86 mmol) in CH_2_Cl_2_ (5 ml) was added TEA (435.62 mg, 4.31 mmol). The mixture was stirred at 50 °C for 2 h. The reaction was monitored by TLC. The mixture was quenched with water (50 ml) at room temperature and extracted with EtOAc (2 × 50 ml). Combined organic layers were washed with brine, dried (Na_2_SO_4_), and concentrated in vacuum. The residue was purified *via* column chromatography (petroleum ether/EtOAc = 4: 1, v/v) to give intermediate **18c** (230 mg, yield 75%) as a white solid. ^1^H NMR (500 MHz, DMSO-*d*_6_) δ 7.66 (d, *J* = 7.3 Hz, 1H), 4.42 − 4.27 (m, 4H), 4.16 − 3.97 (m, 1H), 3.92 − 3.82 (m, 2H), 3.39 (td, *J* = 11.8, 2.0 Hz, 2H), 1.83 − 1.75 (m, 2H), 1.52 (dt, *J* = 12.0, 6.0 Hz, 2H), 1.45 (d, *J* = 9.0 Hz, 9H).

#### Tert-butyl 2-(1H-indol-4-yl)-4-((tetrahydro-2H-pyran-4-yl)amino)-5,7-dihydro-6H-pyrrolo[3,4-d]pyrimidine-6-carboxylate (20c)

To a solution of compound **18c** (120.0 mg, 0.34 mmol) and compound **19**(123.6 mg, 0.51 mmol) in 1,4-dioxane (6 ml) and H_2_O (2 ml) was added K_3_PO_4_ (143.74 mg, 0.68 mmol). The system was purged with nitrogen gas for 10 min, Pd(PPh_3_)_4_ (39.17 mg, 0.03 mmol) was added and the reaction system was purged one more time with nitrogen gas. The reaction mixture was stirred at 100 °C for 6 h. The reaction mixture was then cooled to room temperature the solvent was removed under reduced pressure. The mixture was quenched with water (30 ml) and extracted with EtOAc (2 × 30 ml). Combined organic layers were washed with brine, dried (Na_2_SO_4_), and concentrated in vacuum. The solvent was removed under reduced pressure and the residue was purified *via* silica column chromatography to give compound **20c** (114 mg, yield 78%).^1^H NMR (500 MHz, DMSO-*d*_6_) δ 11.20 (s, 1H), 8.08 (ddd, *J* = 7.5, 4.7, 1.0 Hz, 1H), 7.50 (dt, *J* = 7.9, 1.0 Hz, 1H), 7.43 (q, *J* = 2.8 Hz, 1H), 7.40 − 7.36 (m, 1H), 7.17 (q, *J* = 7.8, 7.0 Hz, 2H), 4.47 (td, *J* = 12.8, 10.7, 2.6 Hz, 4H), 4.00 − 3.92 (m, 2H), 3.47 (td, *J* = 11.7, 2.1 Hz, 2H), 2.02 − 1.92 (m, 2H), 1.60 (tt, *J* = 11.8, 6.0 Hz, 2H), 1.48 (d, *J* = 7.0 Hz, 9H).

#### 2-(1H-indol-4-yl)-N-(tetrahydro-2H-pyran-4-yl)-6,7-dihydro-5H-pyrrolo[3,4-d]p yrimidin-4-amine (21c)

To a solution of intermediate **20c** (114 mg, 0.26 mmol) in methanol (6 ml) at room temperature was added HCl dioxane solution (2 M, 3 ml). After stirring at room temperature environment for 6 h, the mixture was concentrated under reduced pressure to yield crude product **21c** (110.0 mg, yield 96.5%) as a yellow solid. The crude was used directly in the next step of reaction.

#### 2–(3-(2-(1H-indol-4-yl)-4-((tetrahydro-2H-pyran-4-yl)amino)-6,7-dihydro-5H-py rrolo[3,4-d]pyrimidine-6-carbonyl)benzyl)-2H-indazole-7-carboxamide (27c)

HOBT (52.02 mg, 0.39 mmol), EDCI (147.53 mg, 0.77 mmol), DIPEA (153.21 mg, 1.18 mmol), and **26** (87.3 mg, 0.30 mmol) were sequentially added to a solution of compound **21c** (110 mg, 0.30 mmol) in DMF (3 ml) was added. The reaction mixture was stirred at room temperature for 4 h. The solvent was removed under reduced pressure. The mixture was quenched with water (30 ml) and extracted with EtOAc (2 × 30 ml). Combined organic layers were washed with brine, dried (Na_2_SO_4_). After filtering, the solvent was removed under reduced pressure. The crude was purified *via* column chromatography (CH_2_Cl_2_/MeOH = 10: 1, v/v) to give compound **27c** (40.0 mg, yield 22%) as a white solid. M.p. 260.7 − 262.4 °C.^1^H NMR (500 MHz, DMSO-*d*_6_) δ 11.20 (d, *J* = 6.0 Hz, 1H), 8.78 (d, *J* = 1.0 Hz, 1H), 8.56 (dd, *J* = 11.1, 3.1 Hz, 1H), 8.09 (ddd, *J* = 18.1, 7.6, 1.0 Hz, 1H), 7.99 (ddt, *J* = 8.3, 7.0, 1.2 Hz, 2H), 7.84 − 7.58 (m, 3H), 7.51 (ddd, *J* = 9.1, 6.5, 4.5 Hz, 3H), 7.43 (dt, *J* = 9.6, 2.8 Hz, 1H), 7.37 (dt, *J* = 13.5, 2.7 Hz, 1H), 7.32 − 7.03 (m, 3H), 5.83 (d, *J* = 2.0 Hz, 2H), 4.77 − 4.50 (m, 4H), 4.47 − 4.26 (m, 1H), 4.01 − 3.84 (m, 2H), 3.55 − 3.40 (m, 2H), 2.06 − 1.84 (m, 2H), 1.56 (dqd, *J* = 57.9, 12.1, 4.4 Hz, 2H).

#### Tert-butyl (S)-2-chloro-4–(3-methylmorpholino)-5,7-dihydro-6H-pyrrolo[3,4-d]pyrimidine-6-carboxylate (18d)

To a solution of compound **16** (250.0 mg, 0.86 mmol) and **17d** (95.79 mg, 0.95 mmol) in CH_2_Cl_2_ (5 ml) was added TEA (435.62 mg, 4.31 mmol). The mixture was stirred at 50 °C for 2 h. The reaction was monitored by TLC. The mixture was quenched with water (50 ml) at room temperature and extracted with EtOAc (2 × 50 ml). Combined organic layers were washed with brine, dried (Na_2_SO_4_), and concentrated in vacuum. The residue was purified *via* column chromatography (petroleum ether/EtOAc = 4: 1, v/v) to give intermediate **18d** (220 mg, yield 72%) as a white solid. ^1^H NMR (500 MHz, DMSO-*d*_6_) δ 4.76 (dt, *J* = 13.5, 2.6 Hz, 1H), 4.70 − 4.58 (m, 2H), 4.49 − 4.33 (m, 2H), 4.20 (d, *J* = 55.4 Hz, 1H), 3.94 − 3.88 (m, 2H), 3.74 − 3.65 (m, 1H), 3.65 − 3.54 (m, 1H), 3.44 (td, *J* = 11.8, 2.8 Hz, 1H), 1.45 (d, *J* = 3.1 Hz, 9H), 1.24 (dd, *J* = 7.0, 2.0 Hz, 3H).

#### Tert-butyl (S)-2-(1H-indol-4-yl)-4–(3-methylmorpholino)-5,7-dihydro-6H-pyrrolo [3,4-d]pyrimidine-6-carboxylate (20d)

To a solution of compound **18d** (220 mg, 0.62 mmol) and compound **19** (226.1 mg, 0.93 mmol) in 1,4-dioxane (6 ml) and H_2_O (2 ml) was added K_3_PO_4_ (262.46 mg, 1.24 mmol). The system was purged with nitrogen gas for 10 min, Pd(PPh_3_)_4_ (39.17 mg, 0.03 mmol) was added and the reaction system was purged one more time with nitrogen gas. The reaction mixture was stirred at 100 °C for 6 h. The reaction mixture was cooled to room temperature the solvent was removed under reduced pressure. The mixture was quenched with water (50 ml) and extracted with EtOAc (2 × 50 ml). Combined organic layers were washed with brine, dried (Na_2_SO_4_), and concentrated under reduced pressure. The crude was purified *via* column chromatography to give compound **20d** (231 mg, yield 71.7%).^1^H NMR (500 MHz, DMSO-*d*_6_) δ 11.23 (s, 1H), 8.04 (dd, *J* = 7.4, 1.1 Hz, 1H), 7.64 − 7.60 (m, 1H), 7.52 (dt, *J* = 7.9, 0.9 Hz, 1H), 7.45 − 7.41 (m, 1H), 7.29 (d, *J* = 2.8 Hz, 1H), 7.17 (t, *J* = 7.8 Hz, 1H), 4.87 − 4.72 (m, 3H), 4.49 (dd, *J* = 25.8, 9.5 Hz, 3H), 3.98 (dd, *J* = 11.4, 3.4 Hz, 1H), 3.77 (dd, *J* = 11.5, 5.6 Hz, 1H), 3.69 (dd, *J* = 11.5, 3.0 Hz, 1H), 3.54 (td, *J* = 11.7, 2.8 Hz, 1H), 1.48 (s, 9H), 1.30 (d, *J* = 6.8 Hz, 3H).

#### (S)-4–(2-(1H-indol-4-yl)-6,7-dihydro-5H-pyrrolo[3,4-d]pyrimidin-4-yl)-3-methy-lmorpholine (21d)

To a solution of intermediate **20d** (231 mg, 0.53 mmol) in methanol (6 ml) at room temperature was added HCl dioxane solution (2 M, 3 ml). After stirring at room temperature environment for 6 h, the mixture was concentrated under reduced pressure to yield crude product **21d** (230 mg, yield 99.6%) as a yellow solid. The crude was used directly in the next step of reaction.

#### (S)-2–(3-(2-(1H-indol-4-yl)-4–(3-methylmorpholino)-6,7-dihydro-5H-pyrrolo[3,4 -d]pyrimidine-6-carbonyl)benzyl)-2H-indazole-7-carboxamide (27d)

HOBT (71 mg, 0.53 mmol), EDCI (201.4 mg, 1.05 mmol), DIPEA (209.1 mg, 1.61 mmol), and **26** (119 mg, 0.40 mmol) were sequentially added to a solution of compound **21d** (150 mg, 0.40 mmol) in DMF (3 ml). The reaction mixture was stirred at room temperature for 4 h. After completion, the solvent was removed under reduced pressure. The mixture was quenched with water (30 ml) and extracted with EtOAc (2 × 30 ml). Combined organic layers were washed with brine, dried (Na_2_SO_4_). After filtering, the solvent was removed under reduced pressure. The crude was purified *via* column chromatography (CH_2_Cl_2_/MeOH = 10: 1, v/v) to give compound **27d** (32.0 mg, yield 13.0%) as a white solid. M.p. 272.1 − 274.8 °C. ^1^H NMR (500 MHz, DMSO-*d*_6_) δ 11.24 (d, *J* = 7.9 Hz, 1H), 8.78 (d, *J* = 2.8 Hz, 1H), 8.56 (d, *J* = 3.3 Hz, 1H), 8.08 − 7.93 (m, 3H), 7.85 − 7.61 (m, 3H), 7.60 − 7.48 (m, 3H), 7.42 (dt, *J* = 11.4, 2.6 Hz, 1H), 7.32 − 7.13 (m, 3H), 5.83 (d, *J* = 12.1 Hz, 2H), 5.14 − 4.92 (m, 2H), 4.74 (d, *J* = 9.6 Hz, 2H), 4.39 − 4.11 (m, 1H), 4.01 (d, *J* = 10.2 Hz, 1H), 3.91 − 3.36 (m, 5H), 1.35 – 1.20 (m, 3H).

#### Tert-butyl 2-chloro-4-(tetrahydro-1H-furo[3,4-c]pyrrol-5(3H)-yl)-5,7-dihydro-6H-pyrrolo[3,4-d]pyrimidine-6-carboxylate (18e)

To a solution of compound **16** (170 mg, 0.59 mmol) and **17a** (96 mg, 0.65 mmol) in CH_2_Cl_2_ (5 ml) was added TEA (435.62 mg, 4.31 mmol). The mixture was stirred at 50 °C for 2 h. The reaction was monitored by TLC. The mixture was quenched with water (50 ml) at room temperature and extracted with EtOAc (2 × 50 ml). Combined organic layers were washed with brine, dried (Na_2_SO_4_), and concentrated in vacuum. The crude was purified *via* column chromatography (petroleum ether/EtOAc = 4: 1, v/v) to give intermediate **18e** (183 mg, yield 85.5%) as a white solid. ^1^H NMR (500 MHz, DMSO-*d*_6_) δ 4.80 (dt, *J* = 4.9, 2.6 Hz, 2H), 4.34 (dt, *J* = 12.5, 2.5 Hz, 2H), 3.91 − 3.72 (m, 4H), 3.65 − 3.48 (m, 4H), 2.98 (s, 2H), 1.45 (d, *J* = 4.3 Hz, 9H).

#### Tert-butyl 2-(1H-indol-4-yl)-4-(tetrahydro-1H-furo[3,4-c]pyrrol-5(3H)-yl)-5,7-dihydro-6H-pyrrolo[3,4-d]pyrimidine-6-carboxylate (20e)

To a solution of compound **18e** (183 mg, 0.50 mmol) and compound **19** (182.32 mg, 0.75 mmol) in 1,4-dioxane (6 ml) and H_2_O (2 ml) was added K_3_PO_4_ (212 mg, 1.00 mmol). The system was purged with nitrogen gas for 10 min, Pd(PPh_3_)_4_ (57.78 mg, 0.05 mmol) was added and the reaction system was purged one more time with nitrogen gas. The reaction mixture was stirred at 100 °C for 6 h. The reaction mixture was cooled to room temperature the solvent was removed under reduced pressure. The mixture was quenched with water (30 ml) and extracted with EtOAc (2 × 30 ml). Combined organic layers were washed with brine, dried (Na_2_SO_4_), and concentrated in vacuum. The solvent was removed under reduced pressure and the residue was purified *via* silica column chromatography to give compound **20e** (140 mg, yield 62.8%). ^1^H NMR (500 MHz, DMSO-*d*_6_) δ 11.20 (s, 1H), 8.11 − 8.08 (m, 1H), 7.62 (tdd, *J* = 7.2, 3.1, 1.8 Hz, 1H), 7.51 (dt, *J* = 8.1, 0.9 Hz, 1H), 7.43 − 7.39 (m, 2H), 7.16 (t, *J* = 7.8 Hz, 1H), 4.87 (dt, *J* = 5.0, 2.4 Hz, 2H), 4.51 − 4.42 (m, 2H), 3.96 (td, *J* = 7.7, 3.7 Hz, 2H), 3.85 (ddd, *J* = 8.9, 5.5, 2.0 Hz, 2H), 3.69 − 3.63 (m, 4H), 1.48 (d, *J* = 2.2 Hz, 9H).

#### 5–(2-(1H-indol-4-yl)-6,7-dihydro-5H-pyrrolo[3,4-d]pyrimidin-4-yl)hexahydro-1H-furo[3,4-c]pyrrole (21e)

To a solution of intermediate **20e** (140 mg, 0.38 mmol) in methanol (6 ml) at room temperature was added HCl dioxane solution (2 M, 3 ml). After stirring at room temperature environment for 6 h, the mixture was concentrated under reduced pressure to yield crude product **21e** (136 mg, yield 97.1%) as a yellow solid. The crude was used directly in the next step of reaction.

#### 2–(3-(2-(1H-indol-4-yl)-4-(tetrahydro-1H-furo[3,4-c]pyrrol-5(3H)-yl)-6,7-dihydr o-5H-pyrrolo[3,4-d]pyrimidine-6-carbonyl)benzyl)-2H-indazole-7-carboxamide (27e)

HOBT (35.3 mg, 0.26 mmol), EDCI (100.8 mg, 0.53 mmol), DIPEA (104.04 mg, 0.80 mmol), and **26** (59.23 mg, 0.20 mmol) were sequentially added to a solution of compound **21e** (77.00 mg, 0.20 mmol) in DMF (3 ml). The reaction mixture was stirred at room temperature for 4 h. The solvent was removed under reduced pressure. The mixture was quenched with water (30 ml) and extracted with EtOAc (2 × 30 ml). Combined organic layers were washed with brine, dried (Na_2_SO_4_). After filtering, the solvent was removed under reduced pressure. The crude was purified *via* column chromatography (CH_2_Cl_2_/MeOH = 10: 1, v/v) to give compound **27e** (30.0 mg, yield 24.0%) as a white solid. M.p. 281.7 − 283.5 °C. ^1^H NMR (500 MHz, DMSO-*d*_6_) δ 11.21 (s, 1H), 8.78 (d, *J* = 1.0 Hz, 1H), 8.57 (s, 1H), 8.15 − 8.07 (m, 1H), 8.04 − 7.97 (m, 2H), 7.81 (s, 1H), 7.73 (d, *J* = 13.1 Hz, 1H), 7.68 − 7.63 (m, 1H), 7.60 − 7.49 (m, 3H), 7.45 − 7.35 (m, 2H), 7.26 − 7.12 (m, 2H), 5.87 − 5.73 (m, 2H), 5.19 − 5.05 (m, 2H), 4.72 (d, *J* = 8.2 Hz, 2H), 4.02 (dd, *J* = 10.6, 7.3 Hz, 1H), 3.86 (dd, *J* = 8.7, 6.4 Hz, 1H), 3.82 − 3.65 (m, 4H), 3.56 − 3.48 (m, 1H), 2.99 (d, *J* = 76.2 Hz, 2H).

#### Tert-butyl 2-(1H-indol-4-yl)-5,7-dihydro-6H-pyrrolo[3,4-d]pyrimidine-6-carboxy late (20f)

To a solution of compound **18f** (250 mg, 0.98 mmol) and compound **19** (357.45 mg, 1.47 mmol) in 1,4-dioxane (6 ml) and H_2_O (2 ml) was added K_3_PO_4_ (414 mg, 1.96 mmol). The system was purged with nitrogen gas for 10 min, Pd(PPh_3_)_4_ (113.25 mg, 0.10 mmol) was added and the reaction system was purged one more time with nitrogen gas. The reaction mixture was stirred at 100 °C for 6 h. The reaction mixture was cooled to room temperature the solvent was removed under reduced pressure. The mixture was quenched with water (50 ml) and extracted with EtOAc (2 × 50 ml). Combined organic layers were washed with brine, dried (Na_2_SO_4_), and concentrated in vacuum. The solvent was removed under reduced pressure and the residue was purified *via* silica column chromatography to give compound **20f** (241 mg, yield 73.3%). LCMS *m/z* [M + H]^+^ = 337.4.

#### 2-(1H-indol-4-yl)-6,7-dihydro-5H-pyrrolo[3,4-d]pyrimidine (21f)

To a solution of intermediate **20f** (241 mg, 0.73 mmol) in methanol (6 ml) at room temperature was added HCl dioxane solution (2 M, 3 ml). After stirring at room temperature environment for 6 h, the mixture was concentrated under reduced pressure to yield crude product **21f** (240.0 mg, yield 99.5%) as a yellow solid. The crude was used directly in the next step of reaction.

#### 2–(3-(2-(1H-indol-4-yl)-6,7-dihydro-5H-pyrrolo[3,4-d]pyrimidine-6-carbonyl)be nzyl)-2,7a-dihydro-1l2-indazole-7-carboxamide (27f)

HOBT (74.3 mg, 0.55 mmol), EDCI (210.0 mg, 1.10 mmol), DIPEA (218.9 mg, 1.69 mmol), and **26** (125.0 mg, 0.42 mmol) were sequentially added to a solution of compound **21f** (100.0 mg, 0.42 mmol) in DMF (3 ml) was added. The reaction mixture was stirred at room temperature for 4 h. After completion, the solvent was removed under reduced pressure. The mixture was quenched with water (30 ml) and extracted with EtOAc (2 × 30 ml). Combined organic layers were washed with brine, dried (Na_2_SO_4_), and concentrated under reduced pressure. The crude was purified *via* column chromatography (CH_2_Cl_2_/MeOH = 10: 1, v/v) to give compound **27f** (32 mg, yield 15%) as a white solid. M.p. 274.8 − 276.2 °C. ^1^H NMR (500 MHz, DMSO-*d*_6_) δ 11.30 (d, *J* = 7.9 Hz, 1H), 8.97 − 8.77 (m, 2H), 8.55 (s, 1H), 8.19 − 8.11 (m, 1H), 8.04 – 7.93 (m, 2H), 7.85 − 7.50 (m, 7H), 7.49 − 7.35 (m, 2H), 7.22 (td, *J* = 8.4, 7.2 Hz, 2H), 5.85 (s, 2H), 4.98 − 4.79 (m, 4H).

#### Tert-butyl 2-chloro-4-(diethylamino)-5,7-dihydro-6H-pyrrolo[3,4-d]pyrimidine-6-carboxylate (18 g)

To a solution of compound **16** (250.0 mg, 0.86 mmol) and **17 g** (69.20 mg, 0.95 mmol) in CH_2_Cl_2_ (5 ml) was added TEA (435.62 mg, 4.31 mmol). After addition, the mixture was stirred at 50 °C for 2 h. The reaction was monitored by TLC. The mixture was quenched with water (50 ml) at room temperature and extracted with EtOAc (2 × 50 ml). Combined organic layers were washed with brine, dried (Na_2_SO_4_), and concentrated under reduced pressure. The crude was purified *via* column chromatography (petroleum ether/EtOAc = 4: 1, v/v) to give intermediate **18 g** (222 mg, yield 79.0%) as a white solid. ^1^H NMR (500 MHz, DMSO-*d*_6_) δ 4.70 (dt, *J* = 8.6, 2.6 Hz, 2H), 4.33 (dt, *J* = 11.0, 2.4 Hz, 2H), 3.50 − 3.42 (m, 4H), 1.45 (s, 9H), 1.14 (t, *J* = 7.0 Hz, 6H).

#### Tert-butyl 4-(diethylamino)-2-(1H-indol-4-yl)-5,7-dihydro-6H-pyrrolo[3,4-d]pyr-imidine-6-carboxylate (20 g)

To a solution of compound **18 g** (222 mg, 0.68 mmol) and compound **19** (248.33 mg, 1.02 mmol) in 1,4-dioxane (6 ml) and H_2_O (2 ml) was added K_3_PO_4_ (288.0 mg, 1.36 mmol). The system was purged with nitrogen gas for 10 min, Pd(PPh_3_)_4_ (78.58 mg, 0.07 mmol) was added and the reaction system was purged one more time with nitrogen gas. The reaction mixture was stirred at 100 °C for 6 h. After completion, the reaction mixture was cooled to room temperature the solvent was removed under reduced pressure. The mixture was quenched with water (50 ml) and extracted with EtOAc (2 × 50 ml). Combined organic layers were washed with brine, dried (Na_2_SO_4_), and concentrated under reduced pressure. The residue was purified *via* silica column chromatography to give compound **20 g** (212 mg, yield 76.8%). ^1^H NMR (500 MHz, DMSO-*d*_6_) δ 7.64 − 7.60 (m, 3H), 7.57 − 7.53 (m, 2H), 4.87 − 4.63 (m, 2H), 4.53 − 4.26 (m, 2H), 4.10 (q, *J* = 5.2 Hz, 4H), 1.46 (d, *J* = 17.3 Hz, 9H), 1.23 (td, *J* = 6.9, 1.8 Hz, 3H), 1.20 − 1.08 (m, 3H).

#### N,N-diethyl-2-(1H-indol-4-yl)-6,7-dihydro-5H-pyrrolo[3,4-d]pyrimidin-4-amine (21 g)

To a solution of intermediate **20 g** (212 mg, 0.52 mmol) in methanol (6 ml) at room temperature was added HCl dioxane solution (2 M, 3 ml). After stirring at room temperature environment for 6 h, the mixture was concentrated under reduced pressure to yield crude product **21 g** (210.0 mg, yield 99%) as a yellow solid. The crude was used directly in the next step of reaction.

#### 2–(3-(4-(Diethylamino)-2-(1H-indol-4-yl)-6,7-dihydro-5H-pyrrolo[3,4-d]pyrami d-ine-6-carbonyl)benzyl)-2H-indazole-7-carboxamide (27 g)

HOBT (56.2 mg, 0.42 mmol), EDCI (162.6 mg, 0.85 mmol), DIPEA (168.7 mg, 1.3 mmol), and **26** (96.2 mg, 0.33 mmol) were sequentially added to a solution of compound **21 g** (100 mg, 0.33 mmol) in DMF (3 ml). The reaction mixture was stirred at room temperature for 4 h. After completion, the solvent was removed under reduced pressure. The mixture was quenched with water (30 ml) and extracted with EtOAc (2 × 30 ml). Combined organic layers were washed with brine, dried (Na_2_SO_4_), and concentrated under reduced pressure. The crude was purified *via* column chromatography (CH_2_Cl_2_/MeOH = 10: 1, v/v) to give compound **27 g** (68 mg, yield 36%) as a white solid. M.p. 256.7 − 259.5 °C. ^1^H NMR (500 MHz, DMSO-*d*_6_) δ 11.21 (d, *J* = 7.8 Hz, 1H), 8.78 (d, *J* = 2.3 Hz, 1H), 8.56 (d, *J* = 3.5 Hz, 1H), 8.13 − 7.92 (m, 3H), 7.85 − 7.55 (m, 3H), 7.55 − 7.48 (m, 3H), 7.45 − 7.29 (m, 2H), 7.26 − 7.12 (m, 2H), 5.83 (d, *J* = 14.2 Hz, 2H), 5.15 − 4.85 (m, 2H), 4.70 (d, *J* = 9.5 Hz, 2H), 3.75 − 3.36 (m, 4H), 1.26 (t, *J* = 6.9 Hz, 4H), 1.03 (t, *J* = 7.0 Hz, 2H).

#### Tert-butyl 4-(azetidin-1-yl)-2-chloro-5,7-dihydro-6H-pyrrolo[3,4-d]pyrimidine-6-carboxylate (18h)

To a solution of compound **16** (250.0 mg, 0.86 mmol) and **17h** (88.67 mg, 0.95 mmol) in CH_2_Cl_2_ (5 ml) was added TEA (435.62 mg, 4.31 mmol). After addition, the mixture was stirred at 50 °C for 2 h. The reaction was monitored by TLC. The mixture was quenched with water (50 ml) at room temperature and extracted with EtOAc (2 × 50 ml). Combined organic layers were washed with brine, dried (Na_2_SO_4_), and concentrated under reduced pressure. The crude was purified *via* column chromatography (petroleum ether/EtOAc = 4: 1, v/v) to give intermediate **18h** (236 mg, yield 88.7%) as a white solid. ^1^H NMR (500 MHz, CDCl_3_) δ 4.60 (dt, *J* = 23.5, 2.6 Hz, 2H), 4.44 (dt, *J* = 31.3, 2.6 Hz, 2H), 4.28 (q, *J* = 7.4 Hz, 4H), 2.44 (td, *J* = 7.7, 3.1 Hz, 2H), 1.49 (s, 9H).

#### Tert-butyl 4-(azetidin-1-yl)-2-(1H-indol-4-yl)-5,7-dihydro-6H-pyrrolo[3,4-d]pyri midine-6-carboxylate (20h)

To a solution of compound **18h** (236.00 mg, 0.76 mmol) and compound **19** (277.52 mg, 1.14 mmol) in 1,4-dioxane (6 ml) and H_2_O (2 ml) was added K_3_PO_4_ (322.66 mg, 1.52 mmol). The system was purged with nitrogen gas for 10 min, Pd(PPh_3_)_4_ (87.82 mg, 0.08 mmol) was added and the reaction system was purged one more time with nitrogen gas. The reaction mixture was stirred at 100 °C for 6 h. After completion, the reaction mixture was cooled to room temperature the solvent was removed under reduced pressure. The mixture was quenched with water (50 ml) and extracted with EtOAc (2 × 50 ml). Combined organic layers were washed with brine, dried (Na_2_SO_4_), and concentrated under reduced pressure. The crude was purified *via* column chromatography to give compound **20h** (200 mg, yield 95%). ^1^H NMR (500 MHz, CDCl_3_) δ 8.28 (s, 1H), 8.21 − 8.16 (m, 1H), 7.54 (d, *J* = 2.7 Hz, 1H), 7.49 (dd, *J* = 8.0, 1.0 Hz, 1H), 4.75 − 4.57 (m, 4H), 4.35 (td, *J* = 7.7, 5.2 Hz, 4H), 2.53 − 2.39 (m, 2H), 0.07 (s, 9H).

#### 4-(Azetidin-1-yl)-2-(1H-indol-4-yl)-6,7-dihydro-5H-pyrrolo[3,4-d]pyrimidine (21h)

To a solution of intermediate **20h** (205.0 mg, 0.69 mmol) in methanol (6 ml) at room temperature was added HCl dioxane solution (2 M, 3 ml). After stirring at room temperature environment for 6 h, the mixture was concentrated under reduced pressure to yield crude product **21h** (201 mg, yield 98%) as a yellow solid. The crude was used directly in the next step of reaction without further purification.

#### 2–(3-(4-(Azetidin-1-yl)-2-(1H-indol-4-yl)-6,7-dihydro-5H-pyrrolo[3,4-d]pyramid -ine-6-carbonyl)benzyl)-2H-indazole-7-carboxamide (27h)

To a solution of compound **21h** (100 mg, 0.31 mmol) in DMF (3 ml) was added HOBT (53.75 mg, 0.40 mmol), EDCI (152.52 mg, 0.80 mmol), DIPEA (158.39 mg, 1.22 mmol), and **26** (90.21 mg, 0.31 mmol). The reaction mixture was stirred at room temperature for 4 h. After completion, the solvent was removed under reduced pressure. The mixture was quenched with water (30 ml) and extracted with EtOAc (2 × 30 ml). Combined organic layers were washed with brine, dried (Na_2_SO_4_), and concentrated under reduced pressure. The crude was purified *via* column chromatography (CH_2_Cl_2_/MeOH = 10: 1, v/v) to give compound **27h** (35 mg, yield 21%) as a white solid. M.p. 262.4 − 264.6 °C. ^1^H NMR (500 MHz, DMSO-*d*_6_) δ 11.25 − 11.16 (m, 1H), 8.76 (d, *J* = 14.5 Hz, 1H), 8.59 − 8.49 (m, 1H), 8.02 − 7.94 (m, 4H), 7.84 – 7.67 (m, 3H), 7.61 (q, *J* = 6.6 Hz, 1H), 7.53 (dt, *J* = 15.5, 7.3 Hz, 4H), 7.45 − 7.38 (m, 3H), 7.24 − 7.12 (m, 2H), 5.86 − 5.75 (m, 2H), 4.78 − 4.59 (m, 3H), 4.31 (dt, *J* = 16.9, 7.6 Hz, 2H), 4.14 − 3.94 (m, 1H).

#### Tert-butyl 2-chloro-4-(cyclopropylamino)-5,7-dihydro-6H-pyrrolo[3,4-d]pyrimidi ne-6-carboxylate (18i)

To a solution of compound **16** (250.0 mg, 0.86 mmol) and **17i** (54.06 mg, 0.95 mmol) in CH_2_Cl_2_ (5 ml) was added TEA (432.62 mg, 4.31 mmol). After addition, the mixture was stirred at 50 °C for 2 h. The reaction was monitored by TLC. The mixture was quenched with water (50 ml) at room temperature and extracted with EtOAc (2 × 50 ml). Combined organic layers was washed with brine, dried (Na_2_SO_4_), and concentrated under reduced pressure. The crude was purified *via* column chromatography (petroleum ether/EtOAc = 4: 1, v/v) to give intermediate **18i** (200 mg, yield 75.2%) as a white solid. ^1^H NMR (500 MHz, DMSO-*d*_6_) δ 7.85 (s, 1H), 4.46 − 4.24 (m, 4H), 2.85 (dq, *J* = 7.2, 3.6 Hz, 1H), 1.44 (d, *J* = 5.0 Hz, 9H), 0.73 (dt, *J* = 6.8, 3.4 Hz, 2H), 0.54 (s, 2H).

#### Tert-butyl 4-(cyclopropylamino)-2-(1H-indol-4-yl)-5,7-dihydro-6H-pyrrolo[3,4-d]pyrimidine-6-carboxylate (20i)

To a solution of compound **18i** (200 mg, 0.59 mmol) and compound **19** (214.5 mg, 0.88 mmol) in 1,4-dioxane (6 ml) and H_2_O (2 ml) was added K_3_PO_4_ (249.31 mg, 1.18 mmol). The system was purged with nitrogen gas for 10 min, Pd(PPh_3_)_4_ (67.95 mg, 0.06 mmol) was added and the reaction system was purged one more time with nitrogen gas. The reaction mixture was stirred at 100 °C for 6 h. After completion, the reaction mixture was cooled to room temperature the solvent was removed under reduced pressure. The mixture was quenched with water (50 ml) and extracted with EtOAc (2 × 50 ml). Combined organic layers were washed with brine, dried (Na_2_SO_4_), and concentrated under reduced pressure. The crude was purified *via* column chromatography to give compound **20i** (200 mg, yield 86.9%). ^1^H NMR (500 MHz, DMSO-*d*_6_) δ 11.17 (s, 1H), 8.13 (dd, *J* = 7.4, 4.9 Hz, 1H), 7.65 − 7.59 (m, 2H), 7.57 − 7.49 (m, 1H), 7.45 − 7.38 (m, 2H), 7.24 − 7.11 (m, 1H), 4.47 (d, *J* = 16.9 Hz, 3H), 4.35 (d, *J* = 12.5 Hz, 1H), 3.10 − 2.96 (m, 1H), 1.48 (d, *J* = 3.1 Hz, 9H), 0.87 − 0.70 (m, 3H), 0.65 − 0.51 (m, 4H).

#### 2-(1H-indol-4-yl)-N-(oxetan-3-yl)-6,7-dihydro-5H-pyrrolo[3,4-d]pyrimidin-4-am ine (21i)

To a solution of intermediate **20i** (200 mg, 0.51 mmol) in methanol (6 ml) at room temperature was added HCl dioxane solution (2 M, 3 ml). After stirring at room temperature environment for 6 h, the mixture was concentrated under reduced pressure to yield crude product **21i** (200.0 mg, yield 100%) as a yellow solid. The crude was used directly in the next step of reaction.

#### 2–(3-(2-(1H-indol-4-yl)-4-(oxetan-3-ylamino)-6,7-dihydro-5H-pyrrolo[3,4-d]pyr-imidine-6-carbonyl)benzyl)-2H-indazole-7-carboxamide (27i)

HOBT (53.75 mg, 0.40 mmol), EDCI (152.52 mg, 0.80 mmol), DIPEA (158.39 mg, 1.22 mmol), and **26** (90.21 mg, 0.31 mmol) were sequentially added to a solution of compound **21i** (100 mg, 0.31 mmol) in DMF (3 ml). The reaction mixture was stirred at room temperature for 4 h. After completion, the solvent was removed under reduced pressure. The mixture was quenched with water (30 ml) and extracted with EtOAc (2 × 30 ml). Combined organic layers were washed with brine, dried (Na_2_SO_4_), and concentrated under reduced pressure. The crude was purified *via* column chromatography (CH_2_Cl_2_/MeOH = 10: 1, v/v) to give compound **27i** (33 mg, yield 19.1%) as a white solid. M.p. 229.7 − 234.4 °C. ^1^H NMR (500 MHz, DMSO-*d*_6_) δ 11.18 (d, *J* = 3.9 Hz, 1H), 8.81 − 8.73 (m, 1H), 8.60 − 8.50 (m, 1H), 8.21 − 8.06 (m, 1H), 7.98 (ddtd, *J* = 8.4, 5.6, 2.9, 2.5, 1.1 Hz, 2H), 7.87 − 7.70 (m, 1H), 7.68 − 7.57 (m, 2H), 7.51 (dh, *J* = 5.6, 2.8 Hz, 3H), 7.45 − 7.35 (m, 2H), 7.25 − 7.13 (m, 2H), 5.83 (d, *J* = 5.9 Hz, 2H), 5.77 (d, *J* = 12.6 Hz, 2H), 4.72 (d, *J* = 4.2 Hz, 2H), 1.24 (d, *J* = 9.0 Hz, 1H), 0.90 − 0.69 (m, 2H), 0.68 − 0.44 (m, 2H).

#### Tert-butyl 2-chloro-4-(oxetan-3-ylamino)-5,7-dihydro-6H-pyrrolo[3,4-d]pyramid -ine-6-carboxylate (18j)

To a solution of compound **16** (200.0 mg, 0.70 mmol) and **17j** (83.0 mg, 0.76 mmol) in CH_2_Cl_2_ (5 ml) was added TEA (435.6 mg, 4.32 mmol). After addition, the mixture was stirred at 50 °C for 2 h. The reaction was monitored by TLC. The mixture was quenched with water (50 ml) at room temperature and extracted with EtOAc (2 × 50 ml). Combined organic layers were washed with brine, dried (Na_2_SO_4_), and concentrated under reduced pressure. The crude was purified *via* column chromatography (petroleum ether/EtOAc = 4: 1, v/v) to give intermediate **18j** (251 mg, yield 89.6%) as a white solid. ^1^H NMR (500 MHz, CDCl_3_) δ 5.26 (td, *J* = 12.9, 6.5 Hz, 1H), 5.03 (dt, *J* = 9.8, 7.0 Hz, 2H), 4.63 − 4.54 (m, 3H), 4.47 (d, *J* = 9.1 Hz, 3H), 1.50 (d, *J* = 9.0 Hz, 9H).

#### Tert-butyl 2-(1H-indol-4-yl)-4-(oxetan-3-ylamino)-5,7-dihydro-6H-pyrrolo[3,4-d]pyrimidine-6-carboxylate (20j)

To a solution of compound **18j** (251 mg, 0.77 mmol) and compound **19** (280.77 mg, 1.15 mmol) in 1,4-dioxane (6 ml) and H_2_O (2 ml) was added K_3_PO_4_ (326.06 mg, 1.54 mmol). The system was purged with nitrogen gas for 10 min, Pd(PPh_3_)_4_ (88.98 mg, 0.08 mmol) was added and the reaction system was purged one more time with nitrogen gas. The reaction mixture was stirred at 100 °C for 6 h. After completion, the reaction mixture was cooled to room temperature the solvent was removed under reduced pressure. The mixture was quenched with water (50 ml) and extracted with EtOAc (2 × 50 ml). Combined organic layers were washed with brine, dried (Na_2_SO_4_), and concentrated under reduced pressure. The crude was purified *via* column chromatography to give compound **20j** (262 mg, yield 83%). ^1^H NMR (500 MHz, CDCl_3_) δ 7.67 (ddt, *J* = 12.0, 6.9, 1.4 Hz, 1H), 7.58 – 7.42 (m, 3H), 7.33 (q, *J* = 2.7 Hz, 1H), 5.11 (q, *J* = 6.9 Hz, 1H), 5.01 (dt, *J* = 9.2, 7.0 Hz, 2H), 4.66 (qt, *J* = 11.6, 5.5 Hz, 3H), 4.72 − 4.44 (m, 3H), 4.48 (s, 3H), 1.24 (s, 9H).

#### 2-(1H-indol-4-yl)-N-(oxetan-3-yl)-6,7-dihydro-5H-pyrrolo[3,4-d]pyrimidin-4-amine (21j)

To a solution of intermediate **20j** (262 mg, 0.64 mmol) in methanol (6 ml) at room temperature was added HCl dioxane solution (2 M, 3 ml). After stirring at room temperature environment for 6 h, the mixture was concentrated under reduced pressure to yield crude product **21j** (260.0 mg, yield 99.2%) as a yellow solid. The crude was used directly in the next step of reaction.

#### 2–(3-(2-(1H-indol-4-yl)-4-(oxetan-3-ylamino)-6,7-dihydro-5H-pyrrolo[3,4-d]pyr-imidine-6-carbonyl)benzyl)-2H-indazole-7-carboxamide (27j)

HOBT (45.1 mg, 0.33 mmol), EDCI (128.1 mg, 0.67 mmol), DIPEA (133.0 mg, 1.02 mmol), and **26** (75.72 mg, 0.26 mmol) were sequentially added to a solution of compound **21j** (150 mg, 0.26 mmol) in DMF (3 ml). The reaction mixture was stirred at room temperature for 4 h. After completion, the solvent was removed under reduced pressure. The mixture was quenched with water (30 ml) and extracted with EtOAc (2 × 30 ml). Combined organic layers were washed with brine, dried (Na_2_SO_4_), and concentrated under reduced pressure. The crude was purified *via* column chromatography (CH_2_Cl_2_/MeOH = 10: 1, v/v) to give compound **27j** (37 mg, yield 25%) as a white solid. M.p. 257.5 − 259.8 °C. ^1^H NMR (500 MHz, DMSO-*d*_6_) δ 11.66 (s, 1H), 8.93 − 8.72 (m, 1H), 8.54 (dd, *J* = 10.1, 3.1 Hz, 1H), 8.09 − 7.91 (m, 2H), 7.79 (d, *J* = 23.5 Hz, 1H), 7.73 – 7.63 (m, 3H), 7.60 – 7.48 (m, 3H), 7.39 (dd, *J* = 10.3, 7.4 Hz, 1H), 7.29 (q, *J* = 7.3 Hz, 1H), 7.21 (q, *J* = 8.2 Hz, 1H), 6.59 (dd, *J* = 6.1, 3.2 Hz, 1H), 5.85 (d, *J* = 8.9 Hz, 2H), 5.35 (d, *J* = 34.9 Hz, 1H), 4.98 − 4.68 (m, 3H), 4.75 (d, *J* = 10.3 Hz, 1H), 4.61 (dt, *J* = 18.3, 11.3 Hz, 1H), 4.50 − 4.17 (m, 2H), 3.70 − 3.40 (m, 2H).

#### Tert-butyl 2-chloro-4-(pyrrolidin-1-yl)-5,7-dihydro-6H-pyrrolo[3,4-d]pyrimidine-6-carboxylate (18k)

To a solution of compound **16** (250.0 mg, 0.86 mmol) and **17k** (101.96 mg, 0.95 mmol) in CH_2_Cl_2_ (5 ml) was added TEA (435.62 mg, 4.31 mmol). After addition, the mixture was stirred at 50 °C for 2 h. The reaction was monitored by TLC. The mixture was quenched with water (50 ml) at room temperature and extracted with EtOAc (2 × 50 ml). Combined organic layers were washed with brine, dried (Na_2_SO_4_), and concentrated under reduced pressure. The crude was purified *via* column chromatography (petroleum ether/EtOAc = 4: 1, v/v) to give intermediate **18k** (250 mg, yield 89.0%) as a white solid. ^1^H NMR (500 MHz, DMSO-*d*_6_) δ 4.81 (dt, *J* = 7.7, 2.6 Hz, 2H), 4.33 (dt, *J* = 12.1, 2.5 Hz, 2H), 3.78 − 3.42 (m, 4H), 1.89 (s, 4H), 1.45 (d, *J* = 2.1 Hz, 9H).

#### Tert-butyl 2-(1H-indol-4-yl)-4-(pyrrolidin-1-yl)-5,7-dihydro-6H-pyrrolo[3,4-d]pyrimidine-6-carboxylate (20k)

To a solution of compound **18k** (250.0 mg, 0.77 mmol) and compound **19** (282.25 mg, 1.16 mmol) in 1,4-dioxane (6 ml) and H_2_O (2 ml) was added K_3_PO_4_ (328.18 mg, 1.55 mmol). The system was purged with nitrogen gas for 10 min, Pd(PPh_3_)_4_ (39.17 mg, 0.03 mmol) was added and the reaction system was purged one more time with nitrogen gas. The reaction mixture was stirred at 100 °C for 6 h. After completion, the reaction mixture was cooled to room temperature the solvent was removed under reduced pressure. The mixture was quenched with water (50 ml) and extracted with EtOAc (2 × 50 ml). Combined organic layers were washed with brine, dried (Na_2_SO_4_), and concentrated under reduced pressure. The crude was purified *via* column chromatography to give compound **20k** (255.0 mg, yield 95%). ^1^H NMR (500 MHz, DMSO-*d*_6_) δ 11.19 (s, 1H), 8.15 − 8.07 (m, 1H), 7.50 (dt, *J* = 8.0, 0.9 Hz, 1H), 7.42 (dt, *J* = 7.2, 2.6 Hz, 2H), 7.16 (t, *J* = 7.7 Hz, 1H), 4.89 (dd, *J* = 6.2, 3.7 Hz, 2H), 4.52 − 4.43 (m, 2H), 3.74 (q, *J* = 6.6, 6.1 Hz, 4H), 1.99 − 1.92 (m, 4H), 1.48 (s, 9H).

#### 2-(1H-indol-4-yl)-4-(pyrrolidin-1-yl)-6,7-dihydro-5H-pyrrolo[3,4-d]pyrimidine (21k)

To a solution of intermediate **20k** (255.0 mg, 0.63 mmol) in methanol (6 ml) at room temperature was added HCl dioxane solution (2 M, 3 ml). After stirring at room temperature environment for 6 h, the mixture was concentrated under reduced pressure to yield crude product **21k** (253.0 mg, yield 99%)) as a yellow solid. The crude was used directly in the next step of reaction.

#### 2–(3-(2-(1H-indol-4-yl)-4-(pyrrolidin-1-yl)-6,7-dihydro-5H-pyrrolo[3,4-d]pyrim-idine-6-carbonyl)benzyl)-2H-indazole-7-carboxamide (27k)

HOBT (56.88 mg, 0.42 mmol), EDCI (161.4 mg, 0.84 mmol), DIPEA (167.7 mg, 1.29 mmol), and **26** (95.0 mg, 0.32 mmol) were sequentially added to a solution of compound **21k** (120 mg, 0.32 mmol) in DMF (3 ml). The reaction mixture was stirred at room temperature for 4 h. After completion, the solvent was removed under reduced pressure. The mixture was quenched with water (30 ml) and extracted with EtOAc (2 × 30 ml). Combined organic layers were washed with brine, dried (Na_2_SO_4_), and concentrated under reduced pressure. The crude was purified *via* column chromatography (CH_2_Cl_2_/MeOH = 10: 1, v/v) to give compound **27k** (42.0 mg, yield 22%) as a white solid. M.p. 263.7 − 266.6 °C. ^1^H NMR (500 MHz, DMSO-*d*_6_) δ 11.23 (d, *J* = 24.3 Hz, 1H), 8.68 (dd, *J* = 108.8, 3.9 Hz, 2H), 8.18 − 7.57 (m, 7H), 7.56 − 7.34 (m, 5H), 7.26 − 7.05 (m, 2H), 5.82 (d, *J* = 13.2 Hz, 2H), 4.87 − 4.64 (m, 2H), 3.66 (d, *J* = 136.0 Hz, 4H), 3.09 − 2.91 (m, 1H), 1.89 (d, *J* = 97.5 Hz, 4H).

### *General procedure* B *for the synthesis of compounds 33a-33b*

#### Methyl 3-((7-carbamoyl-1H-benzo[d]imidazol-2-yl)methyl)benzoate (30a)

To a solution of compound **28** (194.59 mg, 1.29 mmol) and **29a** (250.0 mg, 1.29 mmol) in CH_3_CN (10 ml) was added CDI (250.58 mg, 1.55 mmol). After addition, the mixture was stirred at 45 °C for 2 h. The reaction was monitored by TLC. The mixture was quenched with water (30 ml) at room temperature and extracted with EtOAc (2 × 30 ml). Combined organic layers were washed with brine, dried (Na_2_SO_4_), and concentrated under reduced pressure. The crude was purified *via* column chromatography (CH_2_Cl_2_/MeOH = 10: 1, v/v) to give intermediate **30a** (185.0 mg, yield 46.5%) as a white solid. ^1^H NMR (500 MHz, DMSO-*d*_6_) δ 6.89 (dd, *J* = 8.0, 1.4 Hz, 2H), 6.61 (dd, *J* = 7.5, 1.4 Hz, 2H), 6.35 (t, *J* = 7.7 Hz, 2H), 6.07 (s, 2H), 4.59 (s, 4H), 3.82 (d, *J* = 44.2 Hz, 2H).

#### Methyl 3-((7-carbamoyl-1H-benzo[d]imidazol-2-yl)methyl)benzoate (31a)

To a solution of compound **30a** (185 mg, 0.57 mmol) added CH_3_CO_2_H (3 ml). After addition, the mixture was stirred at 120 °C for 3 h. The reaction was monitored by TLC. The mixture was quenched with water (30 ml) at room temperature and extracted with EtOAc (2 × 30 ml). Combined organic layers were washed with NaHCO_3_ (100 ml), then washed with brine, dried (Na_2_SO_4_) and concentrated under reduced pressure. The crude was purified *via* column chromatography to give intermediate **31a** (154.0 mg, yield 87.5%) as a white solid. ^1^H NMR (500 MHz, DMSO-*d*_6_) δ 12.68 (s, 1H), 7.78 (dd, *J* = 7.6, 1.2 Hz, 2H), 7.64 (t, J = 7.2 Hz, 3H), 7.61 (dd, *J* = 7.9, 1.2 Hz, 2H), 7.24 (t, *J* = 7.7 Hz, 2H), 2.57 (s, 5H).

#### 3-((7-Carbamoyl-1H-benzo[d]imidazol-2-yl)methyl)benzoic acid (32a)

To a solution of compound **31a** (154.0 mg, 0.50 mmol) in MeOH (5 ml) was added to a solution of NaOH (99.67 mg, 2.49 mmol) in H_2_O (1 ml). The mixture was stirred at room temperature overnight until TLC (CH_2_Cl_2_/MeOH = 20: 1) indicating that the reaction was complete. After completion, the pH was adjusted to acidity using aqueous HCl solution (1 N). the mixture was quenched with water (30 ml) and extracted with EtOAc (2 × 30 ml). Combined organic layers were washed with brine, dried (Na_2_SO_4_), and concentrated under reduced pressure. The crude was purified *via* column chromatography to give compound **32a** (135 mg, yield 91.8%) as a pale-yellow solid. The crude product was used directly in the next stage without further purification.

#### (R)-2–(3-(2-(1H-indol-4-yl)-4–(3-methylmorpholino)-6,7-dihydro-5H-pyrrolo[3,4 -d]pyrimidine-6-carbonyl)benzyl)-1H-benzo[d]imidazole-7-carboxamide (33a)

HOBT (80.45 mg, 0.59 mmol), EDCI (228.2 mg, 1.19 mmol), DIPEA (237.06 mg, 1.83 mmol), and **21a** (153.3 mg, 0.46 mmol) were sequentially added to a solution of compound **32a** (135.0 mg, 0.46 mmol) in DMF (3 ml). The reaction mixture was stirred at room temperature for 4 h. After completion, the solvent was removed under reduced pressure. The mixture was quenched with water (30 ml) and extracted with EtOAc (2 × 30 ml). Combined organic layers were washed with brine, dried (Na_2_SO_4_), and concentrated under reduced pressure. The crude was purified *via* column chromatography (CH_2_Cl_2_/MeOH = 20: 1, v/v) to give compound **33a** (64.0 mg, yield 22.9%) as a white solid. M.p. 252.6 − 254.4 °C. ^1^H NMR (500 MHz, DMSO-*d*_6_) δ 12.83 (d, *J* = 5.4 Hz, 1H), 11.23 (d, *J* = 8.9 Hz, 1H), 9.29 (d, *J* = 3.8 Hz, 1H), 8.08 − 8.00 (m, 1H), 7.81 (dd, *J* = 7.6, 1.1 Hz, 1H), 7.75 − 7.63 (m, 4H), 7.61 − 7.38 (m, 7H), 7.32 − 7.24 (m, 2H), 7.17 (q, *J* = 7.9 Hz, 2H), 5.03 (dq, *J* = 25.5, 13.8, 12.6 Hz, 3H), 4.74 (s, 3H), 4.08 − 3.98 (m, 1H), 3.85 − 3.70 (m, 2H), 1.33 (d, *J* = 6.8 Hz, 3H).

#### Methyl 4–(2-((2-amino-3-carbamoylphenyl)amino)-2-oxoethyl)benzoate (30b)

To a solution of compound **28** (389.2 mg, 2.58 mmol) and **29b** (500.0 mg, 2.58 mmol) in ACN (20 ml) was added CDI (501.5 mg, 3.10 mmol). The mixture was then stirred at 45 °C for 2 h. The reaction was monitored by TLC. The mixture was quenched with water (100 ml) at room temperature and extracted with EtOAc (2 × 100 ml). Combined organic layers were washed with brine, dried over Na_2_SO_4_, and concentrated in vacuum. The crude was purified *via* column chromatography (CH_2_Cl_2_/MeOH = 10: 1, v/v) to give intermediate **30b** (580.0 mg, yield 68.8%) as a white solid. LCMS *m/z* [M + H]^+^ = 328.3.

#### Methyl 4-((7-carbamoyl-1H-benzo[d]imidazol-2-yl)methyl)benzoate (31b)

To a solution of compound **30b** (400 mg, 1.22 mmol) added CH_3_CO_2_H (3 ml). After addition, the mixture was stirred at 120 °C for 3 h. The reaction was monitored by TLC. The mixture was quenched with water (100 ml) at room temperature and extracted with EtOAc (2 × 100 ml). Combined organic layers were washed with brine, dried (Na_2_SO_4_), and concentrated under reduced pressure. The crude was purified *via* column chromatography to give intermediate **31b** (345 mg, yield 91.8%) as a white solid. LCMS *m/z* [M + H]^+^ = 310.3.

#### 4-((7-Carbamoyl-1H-benzo[d]imidazol-2-yl)methyl)benzoic acid (32b)

To a solution of compound **31b** (345.0 mg, 1.12 mmol) in MeOH (5 ml) was added to a solution of NaOH (223.0 mg, 5.58 mmol) in H_2_O (1 ml). The mixture was stirred at room temperature overnight until TLC (CH_2_Cl_2_/MeOH = 20: 1) indicating that the reaction was complete. The pH was adjusted to acidity using aqueous HCl solution (1 N). The mixture was quenched with water (30 ml) and extracted with EtOAc (2 × 30 ml). Combined organic layers were washed with brine, dried (Na_2_SO_4_), and concentrated under reduced pressure. The crude was purified *via* column chromatography to give compound **32b** (280 mg, yield 84.8%) as a pale-yellow solid. The crude product was used directly in the next stage without further purification.

#### (R)-2–(4-(2-(1H-indol-4-yl)-4–(3-methylmorpholino)-6,7-dihydro-5H-pyrrolo[3,4 -d]pyrimidine-6-carbonyl)benzyl)-1H-benzo[d]imidazole-7-carboxamide (33b)

HOBT (148 mg, 1.1 mmol), EDCI (420 mg, 2.2 mmol), DIPEA (430 mg, 3.4 mmol), and **21a** (283.0 mg, 0.85 mmol) were sequentially added to a solution of compound **32b** (250.0 mg, 0.85 mmol) in DMF (10 ml) was added. The reaction mixture was stirred at room temperature for 4 h. The solvent was removed under reduced pressure. The mixture was quenched with water (50 ml) and extracted with EtOAc (2 × 50 ml). Combined organic layers were washed with brine, dried (Na_2_SO_4_), and concentrated. under reduced pressure. The crude was purified *via* column chromatography (CH_2_Cl_2_/MeOH = 20: 1, v/v) to give compound **33b** (243 mg, yield 46.9%) as a white solid. M.p. 270.5 − 272.7 °C. ^1^H NMR (500 MHz, DMSO-*d*_6_) δ 12.76 (s, 1H), 11.24 (d, *J* = 14.0 Hz, 1H), 9.31 (d, *J* = 3.5 Hz, 1H), 8.13 − 8.00 (m, 1H), 7.81 (dd, *J* = 7.6, 1.1 Hz, 1H), 7.77 − 7.73 (m, 2H), 7.71 − 7.64 (m, 2H), 7.60 − 7.49 (m, 3H), 7.42 (dt, *J* = 23.4, 2.9 Hz, 1H), 7.31 − 7.26 (m, 2H), 7.17 (dt, *J* = 15.6, 7.9 Hz, 1H), 5.17 − 5.01 (m, 2H), 4.77 (s, 2H), 4.54 (d, *J* = 37.1 Hz, 2H), 4.26 − 3.89 (m, 2H), 3.84 − 3.67 (m, 3H), 3.67 − 3.53 (m, 1H), 1.29 (dd, *J* = 50.7, 6.8 Hz, 3H).

### *General procedure* C *for the synthesis of compounds 38a-38b*

#### Tert-butyl (R)-4–(2-(1H-indol-4-yl)-4–(3-methylmorpholino)-6,7-dihydro-5H-pyrrolo[3,4-d]pyrimidi ne-6-car-bonyl)benzoate (35a)

HOBT (1053.9 mg, 7.8 mmol), EDCI (2990.5 mg, 15.6 mmol), DIPEA (3105.6 mg, 24 mmol), and **34a** (1332.0 mg, 6 mmol) were sequentially added to a solution of compound **21a** (2226.0 mg, 6 mmol) in DMF (10 ml). The reaction mixture was stirred at room temperature for 4 h. After completion, the solvent was removed under reduced pressure. The mixture was quenched with water (100 ml) and extracted with EtOAc (2 × 100 ml). Combined organic layers were washed with brine, dried (Na_2_SO_4_), and concentrated. under reduced pressure. The crude was purified *via* column chromatography (CH_2_Cl_2_/MeOH = 20: 1, v/v) to give compound **35a** (2671.3 mg, yield 82.6%). LCMS *m/z* [M + H]^+^ = 540.6.

#### (R)-4–(2-(1H-indol-4-yl)-4–(3-methylmorpholino)-6,7-dihydro-5H-pyrrolo[3,4-d]pyrimidine-6-carbonyl)benzoic acid (36a)

To a solution of compound **35a** (250 mg, 0.5 mmol) in MeOH (5 ml) was added to a solution of NaOH (200 mg, 5 mmol) in H_2_O (5 ml). The mixture was stirred at room temperature overnight until TLC (CH_2_Cl_2_/MeOH =20: 1) indicating that the reaction was complete. The pH was adjusted to acidity using aqueous HCl solution (1 N). the mixture was quenched with water (50 ml) and extracted with EtOAc (2 × 50 ml). Combined organic layers were washed with brine, dried over Na_2_SO_4_. After filtering, the solvent was removed under reduced pressure and the residue was purified *via* recrystallization to give compound **36a** (230.0 mg, yield 98%) as a pale-yellow solid. The crude product was used directly in the next stage without further purification.

#### (R)-4–(2-(1H-indol-4-yl)-4–(3-methylmorpholino)-6,7-dihydro-5H-pyrrolo[3,4-d]pyrimidine-6-carbonyl)-N-(2-amino-3-carbamoylphenyl)benzamide (37a)

To a solution of **36a** (230.0 mg, 0.48 mmol) in DMF (5 ml) was added HATU (217.19 mg, 0.57 mmol), DIPEA (184.55 mg, 1.43 mmol) and compound **28** (71.9 mg, 0.48 mmol). The reaction mixture was stirred at room temperature for 4 h. After completion, the solvent was removed under reduced pressure. The mixture was quenched with water (30 ml) and extracted with EtOAc (2 × 30 ml). Combined organic layers were washed with brine, dried (Na_2_SO_4_), and concentrated under reduced pressure. The crude was purified *via* column chromatography (CH_2_Cl_2_/MeOH = 10: 1, v/v) to give compound **37a** (200.0 mg, yield 68%). LCMS *m/z* [M + H]^+^ = 617.7.

#### (R)-2–(4-(2-(1H-indol-4-yl)-4–(3-methylmorpholino)-6,7-dihydro-5H-pyrrolo[3,4 -d]pyrimidine-6-carbonyl)phenyl)-1H-benzo[d]imidazole-7-carboxamide (38a)

To a solution of compound **37a** (50.0 mg, 0.08 mmol) added CH_3_CO_2_H (1 ml). After addition, the mixture was stirred at 120 °C for 3 h. We monitored the reaction process with the assistance of TLC. The mixture was quenched with water (10 ml) at room temperature and extracted with EtOAc (2 × 10 ml). Combined organic layers were washed with NaHCO_3_ (10 ml), then washed with brine, dried over Na_2_SO_4_. The crude was purified *via* column chromatography to give intermediate **38a** (30 mg, yield 31.3%) as a white solid. M.p. 250.1 − 252.6 °C. ^1^H NMR (500 MHz, DMSO-*d*_6_) δ 13.57 (s, 1H), 11.24 (d, *J* = 16.6 Hz, 1H), 9.36 (d, *J* = 3.5 Hz, 1H), 8.37 (dd, *J* = 8.3, 1.6 Hz, 2H), 8.11 − 8.01 (m, 1H), 7.90 (td, *J* = 6.9, 1.5 Hz, 3H), 7.84 − 7.76 (m, 2H), 7.52 (dd, *J* = 14.6, 8.0 Hz, 1H), 7.42 − 7.37 (m, 2H), 7.35 − 7.26 (m, 1H), 7.18 (dt, *J* = 17.0, 7.8 Hz, 1H), 5.18 − 5.04 (m, 2H), 4.81 (s, 2H), 4.46 (d, *J* = 56.3 Hz, 1H), 4.03 (d, *J* = 10.9 Hz, 1H), 3.91 (d, *J* = 12.0 Hz, 1H), 3.82 (d, *J* = 11.4 Hz, 1H), 3.78 − 3.67 (m, 1H), 3.60 (ddd, *J* = 23.5, 11.4, 2.9 Hz, 1H), 3.47 (t, *J* = 12.0 Hz, 1H), 1.30 (dd, *J* = 53.2, 6.8 Hz, 3H).

#### Methyl (R)-4-((2-(1H-indol-4-yl)-4–(3-methylmorpholino)-5,7-dihydro-6H-pyrro-lo[3,4-d]pyrimidin-6-yl)methyl)benzoate (35b)

To a solution of compound **21b** (300 mg, 0.8 mmol), **34b** (203.7 mg, 0.89 mmol) in DMF (5 ml) was added DIPEA (209.4 mg, 1.6 mmol). The reaction mixture was stirred at 50 °C for 4 h. After completion, the solvent was removed under reduced pressure. The mixture was quenched with water (50 ml) and extracted with EtOAc (2 × 50 ml). Combined organic layers were washed with brine, dried (Na_2_SO_4_), and concentrated under reduced pressure. The crude was purified *via* column chromatography (CH_2_Cl_2_/MeOH = 10: 1, v/v) to give compound **35b** (265.0 mg, yield 67.4%). ^1^H NMR (500 MHz, DMSO-*d*_6_) δ 11.21 (s, 1H), 8.03 (dd, *J* = 7.5, 1.0 Hz, 1H), 7.99 − 7.95 (m, 2H), 7.56 (d, *J* = 8.2 Hz, 2H), 7.50 (dt, *J* = 7.9, 1.0 Hz, 1H), 7.41 (t, *J* = 2.7 Hz, 1H), 7.28 (ddd, *J* = 3.0, 2.1, 0.9 Hz, 1H), 7.16 (t, *J* = 7.8 Hz, 1H), 4.22 − 4.10 (m, 2H), 4.06 − 3.91 (m, 4H), 3.85 (d, *J* = 5.2 Hz, 5H), 3.76 − 3.63 (m, 2H), 3.50 (td, *J* = 11.7, 2.8 Hz, 1H), 1.26 (d, *J* = 6.8 Hz, 3H).

#### (R)-4-((2-(1H-indol-4-yl)-4–(3-methylmorpholino)-5,7-dihydro-6H-pyrrolo[3,4-d]pyrimidin-6-yl)methyl)benzoic acid (36b)

To a solution of compound **35b** (265.0 mg, 0.54 mmol) in MeOH (10 ml) was added to a solution of NaOH (109.7 mg, 2.74 mmol) in H_2_O (5 ml). The mixture was stirred at room temperature overnight until TLC (CH_2_Cl_2_/MeOH = 20: 1) indicating that the reaction was complete. After completion, the pH was adjusted to acidity using aqueous HCl solution (1 N). the mixture was quenched with water (30 ml) and extracted with EtOAc (2 × 30 ml). Combined organic layers were washed with brine, dried over Na_2_SO_4_. After filtering, the solvent was removed under reduced pressure and the residue was purified *via* recrystallization to give compound **36b** (209 mg, yield 82.6%) as a pale-yellow solid. The crude product was used directly in the next stage without further purification.

#### (R)-4-((2-(1H-indol-4-yl)-4–(3-methylmorpholino)-5,7-dihydro-6H-pyrrolo[3,4-d]pyrimidin-6-yl)methyl)-N-(2-amino-3-carbamoylphenyl)benzamide (37b)

To a solution of **36b** (100.0 mg, 0.21 mmol) in DMF (2 ml) was added HATU (97.18 mg, 0.26 mmol), DIPEA (35.79 mg, 0.28 mmol) and compound **28** (32.19 mg, 0.21 mmol). The reaction mixture was stirred at room temperature for 4 h. The solvent was removed under reduced pressure. The mixture was quenched with water (30 ml) and extracted with EtOAc (2 × 30 ml). Combined organic layers were washed with brine, dried (Na_2_SO_4_), and concentrated under reduced pressure. The crude was purified *via* column chromatography (CH_2_Cl_2_/MeOH = 10: 1, v/v) to give compound **37b** (80.0 mg, yield 62.5%).

*(R)-2–(4-(2-(1H-indol-4-yl)-4–(3-methylmorpholino)-6,7-dihydro-5H-pyrrolo[3,4 -d]pyrimidine-6-carbonyl)phenyl)-1H-benzo[d]imidazole-7-carboxamide (****38b****).* To a solution of compound **37b** (80 mg, 0.13 mmol) added CH_3_CO_2_H (2 ml). After addition, the mixture was stirred at 120 °C for 3 h. We monitored the reaction process with the assistance of TLC. The mixture was quenched with water (30 ml) at room temperature and extracted with EtOAc (2 × 30 ml). Combined organic layers were washed with NaHCO_3_ (100 ml), then washed with brine, dried over Na_2_SO_4_. The crude was purified *via* column chromatography to give intermediate **38b** (20 mg, yield 26.3%) as a white solid. M.p. 253.4 − 255.5 °C. ^1^H NMR (500 MHz, DMSO-*d*_6_) δ 13.40 (s, 1H), 11.21 (s, 1H), 9.38 (d, *J* = 3.5 Hz, 1H), 8.29 − 8.20 (m, 2H), 8.03 (dd, *J* = 7.5, 1.1 Hz, 1H), 7.88 (dd, *J* = 7.6, 1.2 Hz, 1H), 7.81 − 7.71 (m, 2H), 7.64 (d, *J* = 8.2 Hz, 2H), 7.50 (dd, *J* = 7.9, 1.1 Hz, 1H), 7.41 (t, *J* = 2.8 Hz, 1H), 7.35 (t, *J* = 7.8 Hz, 1H), 7.31 − 7.26 (m, 1H), 7.16 (t, *J* = 7.8 Hz, 1H), 4.40 (s, 1H), 4.31 − 4.11 (m, 2H), 4.08 − 3.99 (m, 3H), 3.97 − 3.92 (m, 1H), 3.89 (t, *J* = 2.1 Hz, 2H), 3.74 (d, *J* = 11.4 Hz, 1H), 3.67 (dd, *J* = 11.7, 3.0 Hz, 1H), 3.51 (td, *J* = 11.7, 2.8 Hz, 1H), 1.28 (d, *J* = 6.8 Hz, 3H).

### *General procedure* D *for the synthesis of compounds 42a-42d,43*

*(R)-2-((4–(2-(1H-indol-4-yl)-4–(3-methylmorpholino)-6,7-dihydro-5H-pyrrolo[3,4-d]pyrimidine −6-carbonyl)benzamido)methyl)-1H-benzo[d]imidazole-7-carboxamide (****42a****).* To a solution of **36a** (100.0 mg, 0.21 mmol) in DMF (2 ml) was added HATU (94.4 mg, 0.25 mmol), DIPEA (80.26 mg, 0.62 mmol) and compound **41a** (39.4 mg, 0.21 mmol). The reaction mixture was stirred at room temperature for 4 h. The solvent was then removed under reduced pressure. The mixture was quenched with water (30 ml) and extracted with EtOAc (2 × 30 ml). Combined organic layers were washed with brine, dried over Na_2_SO_4_. After filtering, the solvent was removed under reduced pressure and the crude was purified *via* column chromatography (CH_2_Cl_2_/MeOH = 10: 1, v/v) to give compound **42a** (40.0 mg, yield 29.5%) as a white solid. M.p. 260.4 − 262.8 °C. ^1^H NMR (500 MHz, DMSO-*d*_6_) δ 12.85 (d, *J* = 7.8 Hz, 1H), 11.24 (d, *J* = 14.0 Hz, 1H), 9.46 − 9.25 (m, 2H), 8.03 (dd, *J* = 22.8, 7.4 Hz, 4H), 7.85 − 7.63 (m, 6H), 7.55 − 7.39 (m, 3H), 7.29 (dd, *J* = 17.6, 12.8 Hz, 3H), 7.17 (dt, *J* = 15.5, 7.7 Hz, 1H), 5.14 − 5.01 (m, 2H), 4.83 − 4.70 (m, 5H), 3.96 (ddd, *J* = 58.9, 11.5, 3.4 Hz, 1H), 1.35 − 1.21 (m, 4H).

#### Tert-butyl 4–(7-carbamoyl-1H-benzo[d]imidazol-2-yl)piperidine-1-carboxylate (40b)

To a solution of compound **28** (200 mg, 1.32 mmol) and **39b** (282.16 mg, 1.32 mmol) in methanol (5 ml) was added Pd/C (98.33 mg, 0.92 mmol). After addition, the mixture was stirred at 80 °C for a reflux reaction for 8 h. The reaction was monitored by TLC. Water (30 ml) was added to quench the reaction, and the mixture was extracted with EtOAc (2 × 30 ml). Combined organic layers were washed with brine, dried (Na_2_SO_4_), and concentrated in vacuum. The residue was purified *via* column chromatography (CH_2_Cl_2_/MeOH = 20: 1, v/v) to give intermediate **40b** (208 mg, yield 45.8%) as a white solid.

#### 2-(Piperidin-4-yl)-1H-benzo[d]imidazole-7-carboxamide(41b)

To a solution of intermediate **40b** (208 mg,0.60 mmol) in methanol (5 ml) at room temperature was added HCl dioxane solution (2 M, 3 ml). After stirring at room temperature environment for 6 h, the mixture was concentrated under reduced pressure to yield crude product **41b** (200 mg, yield 100%) as a yellow solid. The crude was used directly in the next step of reaction.

#### R)-2–(1-(4–(2-(1H-indol-4-yl)-4–(3-methylmorpholino)-6,7-dihydro-5H-pyrrolo[3, 4-d]pyrimidine-6-carbonyl)benzoyl)piperidin-4-yl)-1H-benzo[d]imidazole-7-carboxa mide(42b)

HOBT (36.88 mg, 0.27 mmol), EDCI (104.67 mg, 0.55 mmol), DIPEA (108.79 mg, 0.84 mmol), and **41b** (50.51 mg, 0.21 mmol) were sequentially added to a solution of compound **36a** (100 mg, 0.21 mmol) in DMF (3 ml). The reaction mixture was stirred at room temperature for 4 h. After completion, the solvent was removed under reduced pressure. The mixture was then quenched with water (30 ml) and extracted with EtOAc (2 × 30 ml). Combined organic layers were washed with brine, dried (Na_2_SO_4_), and concentrated. under reduced pressure. The crude was purified *via* column chromatography (CH_2_Cl_2_/MeOH = 10: 1, v/v) to give compound **42b** (53 mg, 35.6%) as a white solid. M.p. 275.7 − 277.9 °C. ^1^H NMR (500 MHz, DMSO-*d*_6_) δ 12.76 (s, 1H), 11.24 (d, *J* = 13.9 Hz, 1H), 9.33 − 9.28 (m, 1H), 8.10 − 8.00 (m, 1H), 7.83 − 7.63 (m, 6H), 7.59 − 7.39 (m, 5H), 7.32 − 7.26 (m, 2H), 7.17 (dt, *J* = 15.5, 7.8 Hz, 1H), 5.16 − 5.01 (m, 3H), 4.77 (s, 3H), 4.65 − 4.12 (m, 3H), 4.07 − 3.41 (m, 8H), 1.29 (dd, *J* = 50.7, 6.7 Hz, 4H).

#### Tert-butyl 3–(4-carbamoyl-1H-benzo[d]imidazol-2-yl)pyrrolidine-1-carboxylate (40c)

To a solution of compound **28** (100 mg, 0.66 mmol) and **39c** (131.8 mg, 0.66 mmol) in methanol (5 ml) was added Pd/C (49.3 mg, 0.46 mmol). The mixture was stirred at 80 °C for a reflux reaction for 8 h. The reaction was monitored by TLC. Water (30 ml) was added to quench the reaction, and the mixture was extracted with EtOAc (2 × 30 ml). Combined organic layers were washed with brine, dried (Na_2_SO_4_), and concentrated in vacuum. The residue was purified *via* column chromatography (CH_2_Cl_2_/MeOH = 20: 1, v/v) to give intermediate **40c** (86 mg, yield 38.4%) as a white solid. LCMS *m/z* [M + H]^+^ = 331.4.

#### 2-(Pyrrolidin-3-yl)-1H-benzo[d]imidazole-4-carboxamide (41c)

To a solution of intermediate **40c** (86 mg, 0.16 mmol) in methanol (5 ml) at room temperature was added HCl dioxane solution (2 M, 3 ml). The reaction mixture was stirred at room temperature environment for 6 h. The mixture was then removed under reduced pressure to give **41c** (80.0 mg, yield 82.6%) as a yellow solid, which was used directly without further purification.

#### 2–(1-(4–(2-(1H-indol-4-yl)-4-((R)-3-methylmorpholino)-6,7-dihydro-5H-pyrrolo [3,4-d]pyrimidine-6-carbonyl)benzoyl)pyrrolidin-3-yl)-1H-benzo[d]imidazole-7-carb oxamide (42c)

To a solution of compound **36a** (80.0 mg, 0.16 mmol) in DMF (3 ml) was added HOBT (28.98 mg, 0.22 mmol), EDCI (82.23 mg, 0.43 mmol), DIPEA (85.4 mg, 0.66 mmol), and **41c** (38 mg, 0.16 mmol). After stirring at room temperature for 4 h, TLC showed the reaction was completed. The mixture was then concentrated under reduced pressure. Water (30 ml) was added, and the mixture was extracted with EtOAc (2 × 30 ml). Combined organic layers were washed with brine, dried (Na_2_SO_4_), and concentrated under reduced pressure. The crude was purified *via* column chromatography (CH_2_Cl_2_/MeOH = 10: 1, v/v) to give compound **42c** (60 mg, yield 52.2%) as a white solid. M.p. 257.2 − 259.3 °C. ^1^H NMR (500 MHz, DMSO-*d*_6_) δ 12.97 (d, *J* = 48.7 Hz, 1H), 11.26 (d, *J* = 14.5 Hz, 1H), 9.31 − 9.08 (m, 1H), 8.08 − 8.00 (m, 1H), 7.85 − 7.62 (m, 8H), 7.57 − 7.37 (m, 2H), 7.33 − 7.13 (m, 3H), 5.16 − 5.00 (m, 2H), 4.76 (d, *J* = 14.7 Hz, 2H), 4.35 (d, *J* = 151.2 Hz, 2H), 4.08 − 3.77 (m, 5H), 3.76 − 3.54 (m, 3H), 1.35 − 1.13 (m, 6H).

#### Tert-butyl 2–(4-carbamoyl-1H-benzo[d]imidazol-2-yl)azetidine-1-carboxylate (40d)

To a solution of compound **28** (500 mg, 3.3 mmol) and **39d** (611.0 mg, 3.3 mmol) in methanol (6 ml) was added Pd/C (246.4 mg, 2.3 mmol). After addition, the mixture was stirred at 80 °C for a reflux reaction for 8 h. The reaction was monitored by TLC. Water (100 ml) was added to quench the reaction, and the mixture was extracted with EtOAc (2 × 100 ml). Combined organic layers were washed with brine, dried (Na_2_SO_4_), and concentrated in vacuum. The residue was purified *via* column chromatography (CH_2_Cl_2_/MeOH = 20: 1, v/v) to give intermediate **40d** (548.0 mg, yield 52.6%) as a white solid.

#### 2-(Azetidin-2-yl)-1H-benzo[d]imidazole-4-carboxamide (41d)

Compound **40d** (548 mg, 0.52 mmol) in methanol (5 ml) at room temperature was added HCl dioxane solution (2 M, 3 ml). After stirring at room temperature environment for 6 h, the mixture was concentrated under reduced pressure to yield crude product **41d** (2671.3 mg, yield 82.6%) as a yellow solid. The crude was used directly in the next step of reaction.

#### 2–(1-(4–(2-(1H-indol-4-yl)-4-((R)-3-methylmorpholino)-6,7-dihydro-5H-pyrrolo [3,4-d]pyrimidine-6-carbonyl)benzoyl)azetidin-2-yl)-1H-benzo[d]imidazole-7-carbox amide (42d)

HOBT (54.5 mg, 0.4 mmol), EDCI (154.5 mg, 0.81 mmol), DIPEA (160.5 mg, 1.24 mmol), and **41d** (67.0 mg, 0.31 mmol) were sequentially added to a solution of compound **36a** (150.0 mg, 0.31 mmol) in DMF (3 ml). The reaction mixture was stirred at room temperature for 4 h. The solvent was removed under reduced pressure. The mixture was quenched with water (30 ml) and extracted with EtOAc (2 × 30 ml). Combined organic layers were washed with brine, dried (Na_2_SO_4_), and concentrated under reduced pressure. The crude was purified *via* column chromatography (CH_2_Cl_2_/MeOH = 10: 1, v/v) to give compound **42d** (120.0 mg, yield 56.9%) as a white solid. M.p. 265.3 − 266.7 °C. ^1^H NMR (500 MHz, DMSO-*d*_6_) δ 13.05 (s, 1H), 11.24 (d, *J* = 14.2 Hz, 2H), 8.04 (dd, *J* = 22.3, 7.4 Hz, 1H), 7.86 − 7.65 (m, 7H), 7.55 − 7.43 (m, 2H), 7.40 (t, *J* = 2.7 Hz, 1H), 7.34 − 7.26 (m, 2H), 7.16 (q, *J* = 7.9 Hz, 1H), 5.16 − 5.00 (m, 2H), 4.86 − 4.65 (m, 4H), 4.60 − 4.15 (m, 4H), 4.06 − 3.88 (m, 1H), 3.83 − 3.68 (m, 2H), 3.64 − 3.42 (m, 3H), 1.34 (d, *J* = 6.7 Hz, 3H).

#### 2–(1-(3–(2-(1H-indol-4-yl)-4-((R)-3-methylmorpholino)-6,7-dihydro-5H-pyrrolo [3,4-d]pyrimidine-6-carbonyl)benzoyl)azetidin-2-yl)-1H-benzo[d]imidazole-7-carbox amide (43)

HOBT (54.4 mg, 0.40 mmol), EDCI (154.5 mg, 0.81 mmol), DIPEA (160.5 mg, 1.24 mmol), and **41d** (67.0 mg, 0.31 mmol) were sequentially added to a solution of compound **36** (150.0 mg, 0.31 mmol) in DMF (3 ml). The reaction mixture was stirred at room temperature for 4 h. The solvent was then removed under reduced pressure. The mixture was quenched with water (30 ml) and extracted with EtOAc (2 × 30 ml). Combined organic layers were washed with brine, dried (Na_2_SO_4_). After filtering, the solvent was removed under reduced pressure. The crude was purified *via* column chromatography (CH_2_Cl_2_/MeOH = 10: 1, v/v) to give compound **43** (140.0 mg, yield 66.4%) as a white solid. M.p. 238.4 − 241.3 °C. ^1^H NMR (500 MHz, DMSO-*d*_6_) δ 13.03 (s, 1H), 11.22 (s, 1H), 8.30 − 8.00 (m, 2H), 7.94 − 7.60 (m, 6H), 7.56 − 7.37 (m, 3H), 7.29 (ddd, J = 17.5, 8.8, 4.0 Hz, 3H), 7.21 − 7.13 (m, 1H), 5.09 (d, J = 21.5 Hz, 2H), 4.76 (d, J = 20.9 Hz, 3H), 4.60 − 4.16 (m, 4H), 4.03 (d, J = 10.9 Hz, 2H), 3.81 (d, J = 11.2 Hz, 2H), 3.51 (d, J = 55.7 Hz, 1H), 1.34 (dd, J = 7.1, 3.5 Hz, 4H).

### Cell lines and culture methods

MDA-MB-231 and MDA-MB-468 cells were purchased from the American Type Culture Collection (ATCC, USA) or Cell bank of the Chinese Academy of Sciences (CCAS, China). MDA-MB-231, and MDA-MB-468 were cultured in DMEM (GENOM, GNM12800-5) supplemented with 10% (v/v) FBS and 1% (v/v) penicillin − streptomycin. All cells were grown in a humidified incubator at 37 °C and 5% CO_2_.

### Cell growth inhibition assays

Cells were grown at 37 °C, under 95% air and 5% CO_2_ until about reaching 70% confluence and were subculture at least twice before the experiment. Cells were seeded at the densities of 500 cells/well in two 384-well white-walled tissue culture-treated plates and placed the plate in 5% CO_2_ incubator overnight. Growth viability was determined by CellTiter-Glo luminescent viability assay (Promega) 6 days after drug treatment. Cell viability was measured immediately after dosing (Day 0) and after 6 days of incubation using Cell Titer-Glo (CTG, Promega, G7573). Relative viability was calculated by normalising raw luminescence counts to DMSO control (Ctrl.) treated cells. Half maximal inhibitory concentration (IC_50_) values were calculated using GraphPad Prism 8 software and sigmoidal dose-response curve fitting.

### Cell cycle assay

MDA-MB-231 cells were seeded into 6-well plates and incubated at 37 °C for 24 h, and then treated with the tested compounds (DMSO 0.01%, Niraparib 1.0 μM, AZD6738 1.0 μM, their combination, or **38a** at 1.0/2.0/4.0 μM) for another 48 h. After treatment, cells were collected and fixed with 70% pre-cold ethanol in PBS and stored at 20 °C overnight. Then washed the cells with PBS twice, and incubated with 100 μg/mL RNase A at 37 °C for 1 h, stained with propidium iodide (PI) for 30 min avoid light at room temperature. The stained cells were analysed by a flow cytometer according to the manufacturer’s instructions. The data were analysed by FlowJo software (Tree Star, Inc., Ashland, OR, USA).

### Cell apoptosis assay

MDA-MB-231 cells were seeded into 6-well plates and incubated at 37 °C for 24 h, and then treated with the tested compounds (DMSO 0.01%, Niraparib 1.0 μM, AZD6738 1.0 μM, their combination, or **38a** at 1.0/2.0/4.0 μM) for another 48 h. After treatment, the cells were then harvested by trypsinization and washed twice with cold PBS. After the centrifugation and removal of the supernatant, cells were re-suspended in 500 μL of a 1 × binding buffer, which was then added to 5 μL of annexin VFITC and 10 μL of PI, and incubated at room temperature for 15 min in the dark. The stained cells were analysed by a flow cytometer. The data were analysed by FlowJo software (Tree Star, Inc., Ashland, OR, USA).

### Immunofluorescence staining

Cells were seeded at 5 × 10^4^ cells per coverslip placed in 24-well plates and allowed to attach overnight before exposure to test compounds for 24 h. After treatment, cells were gently rinsed twice with 1 × PBS (pH 7.4, Gibco, USA) and fixed with 4% paraformaldehyde (Sigma-Aldrich, P6148) for 15 min at room temperature (RT). Following three PBS washes, non-specific binding was blocked with 5% bovine serum albumin (BSA; Sigma-Aldrich, A1933) with 0.5% Triton X-100 (Solarbio, China) in PBS for 1 h at 37 °C. Coverslips were then incubated overnight at 4 °C with rabbit anti-γH2AX monoclonal antibody (1:1000; Cell Signalling Technology, #9718). After three PBS washes, Alexa Fluor 488-conjugated goat anti-rabbit IgG (H + L) (1:3000; Invitrogen, A-11008) was applied for 1 h at RT in the dark. Following three additional PBS washes, nuclei were counterstained and mounted with DAPI-containing antifade medium (Beyotime, China). Fluorescence images were acquired on a Nikon Eclipse 90i upright microscope equipped with a QImaging Retiga 200 R CCD camera, keeping acquisition parameters constant.

### Western blot

Cells (9 × 10^5^) were seeded in 6-well plates and incubated overnight. Then treated with DMSO or **38a** for 48 h. Cells were washed with cold PBS and lysed in RIPA buffer containing protease inhibitor (Fude, FD1002) for 30 min on ice. The lysate was centrifuged (13,000 rpm, 4 °C, 20 min); the protein concentrations were determined by the BCA Assay Kit (Beyotime, P0012S). A total of 20 μg of protein was loaded onto 10% SDS-PAGE gel and then transferred onto polyvinylidene fluoride (PVDF, Millipore) membranes. The membranes were blocked with 5% BSA (in TBST) for 1 h, incubated with primary antibodies overnight at 4 °C, and then washed three times with Tris-buffered saline containing 0.1% with Tween 20 (TBST) for 10 min each. After incubating with the secondary HRP antibody for 1 h at RT, the membranes were washed three times with TBST for 10 min each and then exposed on autoradiograph films using enhanced chemiluminescence (ECL). The primary antibodies used were PARP1 (1/1000 dilution, CST#9532), BCL-2(1/1000 dilution, CST#3498), Bax (1/1000 dilution, CST#5023), Caspase-3 (1/1000 dilution, CST#9662), ATR (1/1000 dilution, CST#13934), p-ATR (1/1000 dilution, CST#2853), CHK1 (1/1000 dilution, CST#2360), p-CHK1 (1/1000 dilution, CST#2348), p53 (1/1000 dilution, CST#9282), p-p53 (1/1000 dilution, CST#37909), p-21 (1/1000 dilution, CST#2947), γH2AX (1/1000 dilution, CST#9718) and Actin (1/1000 dilution, Proteintech# 66009–1-Ig).

## Supplementary Material

Supplemental Materials_anonymous.docx

## Data Availability

The authors confirm that the data supporting the findings of this study are available within the article or its supplementary materials.
