# Supplementary material for: Design, synthesis and anti-breast cancer activity evaluation of 6,7-dihydro-5H-pyrrolo[3,4-d]pyrimidine-based PARP1/ATR dual inhibitors
Source: J Enzyme Inhib Med Chem. 2026 Feb 16;41(1):2627053. doi: 10.1080/14756366.2026.2627053 (PMC12912221; doi:10.1080/14756366.2026.2627053)
Supplement: Supplemental Materials_anonymous.docx [file IENZ_A_2627053_SM8251.docx]

**Design, synthesis and anti-breast cancer activity evaluation of 6,7-dihydro-5*H*-pyrrolo[3,4-*d*]pyrimidine-based PARP1/ATR dual inhibitors**

## Table of Content

## Part 1: ^1^H NMR and ^13^C NMR spectra for the target compounds 27a-27k, 33a-33b, 38a-38b, 42a-42d, 43.

**Part 2: HRMS spectra of the target compounds 27a-27k, 33a-33b, 38a-38b, 42a-42d, 43**

**Part 3: HPLC purity data for the lead compound (38a).**

**Part 4: Western Blot.**

**Part 1: ^1^H NMR and ^13^C NMR spectra for the target compounds.**

*(R)-2-(3-(2-(1H-indol-4-yl)-4-(3-methylmorpholino)-6,7-dihydro-5H-pyrrolo[3,4-d]pyrimidine-6-carbonyl)benzyl)-2H-indazole-7-carboxamide (****27a****)*

**
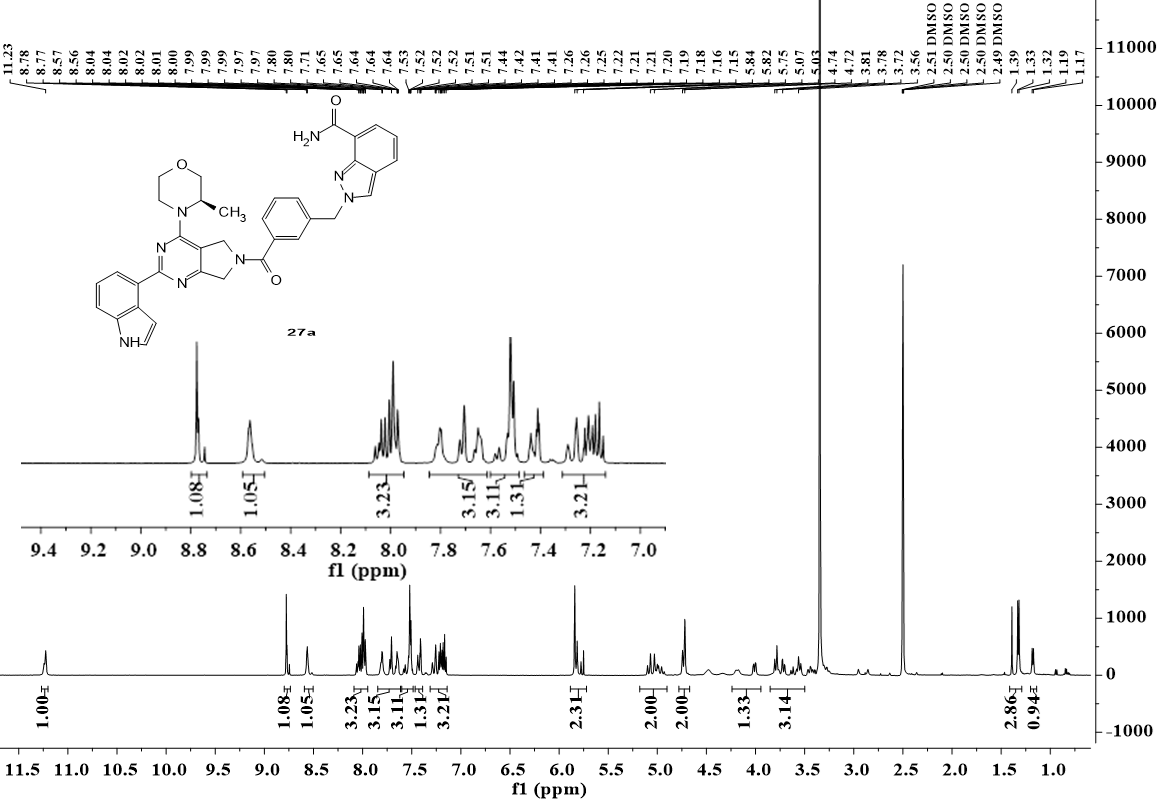
**

*2-(3-(2-(1H-indol-4-yl)-4-morpholino-6,7-dihydro-5H-pyrrolo[3,4-d]pyrimidine-6-carbonyl)benzyl)-2H-indazole-7-carboxamide (****27b****)*

**
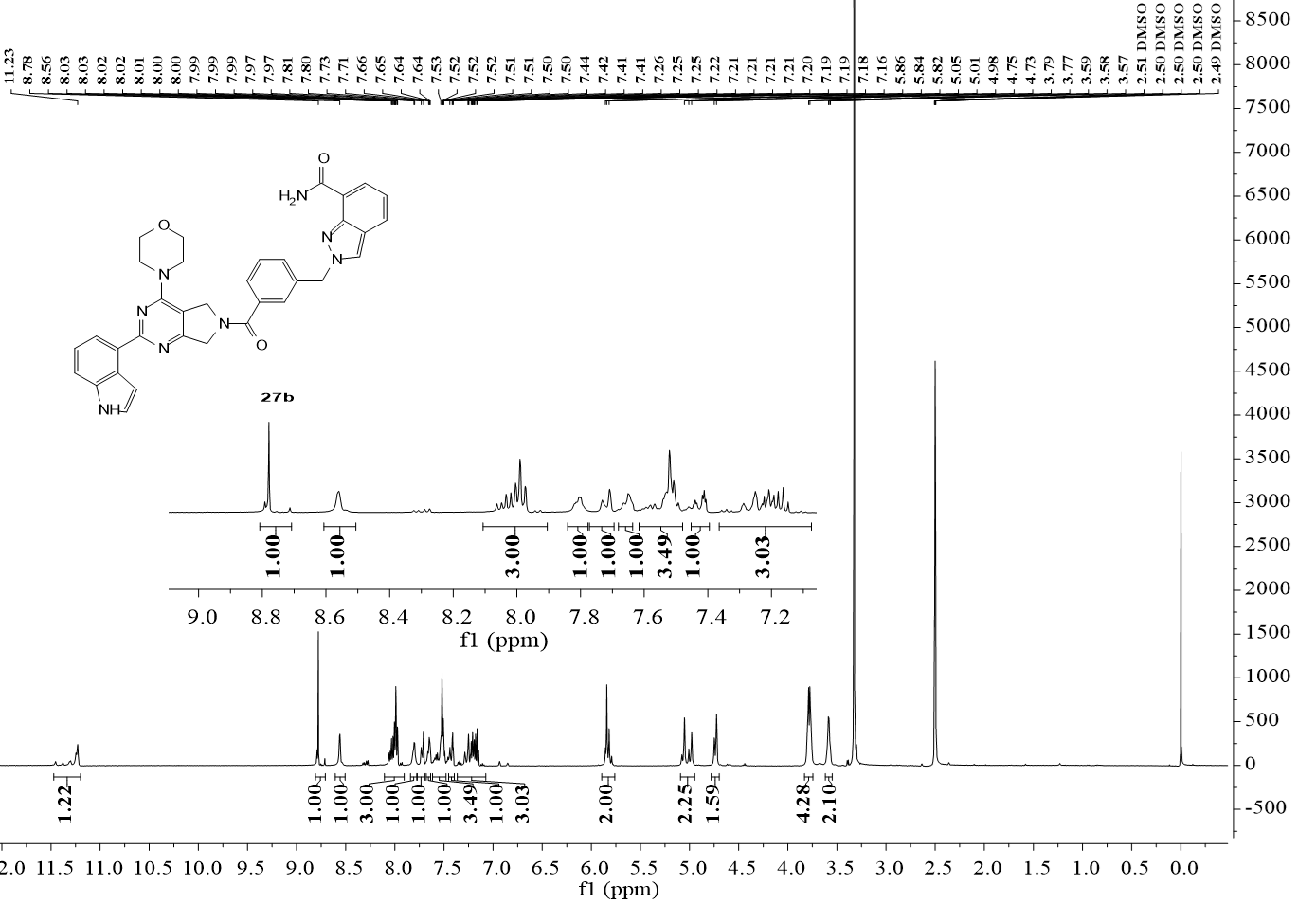
**

*2-(3-(2-(1H-indol-4-yl)-4-((tetrahydro-2H-pyran-4-yl)amino)-6,7-dihydro-5H-pyrrol o[3,4-d]pyrimidine-6-carbonyl)benzyl)-2H-indazole-7-carboxamide (****27c****)*


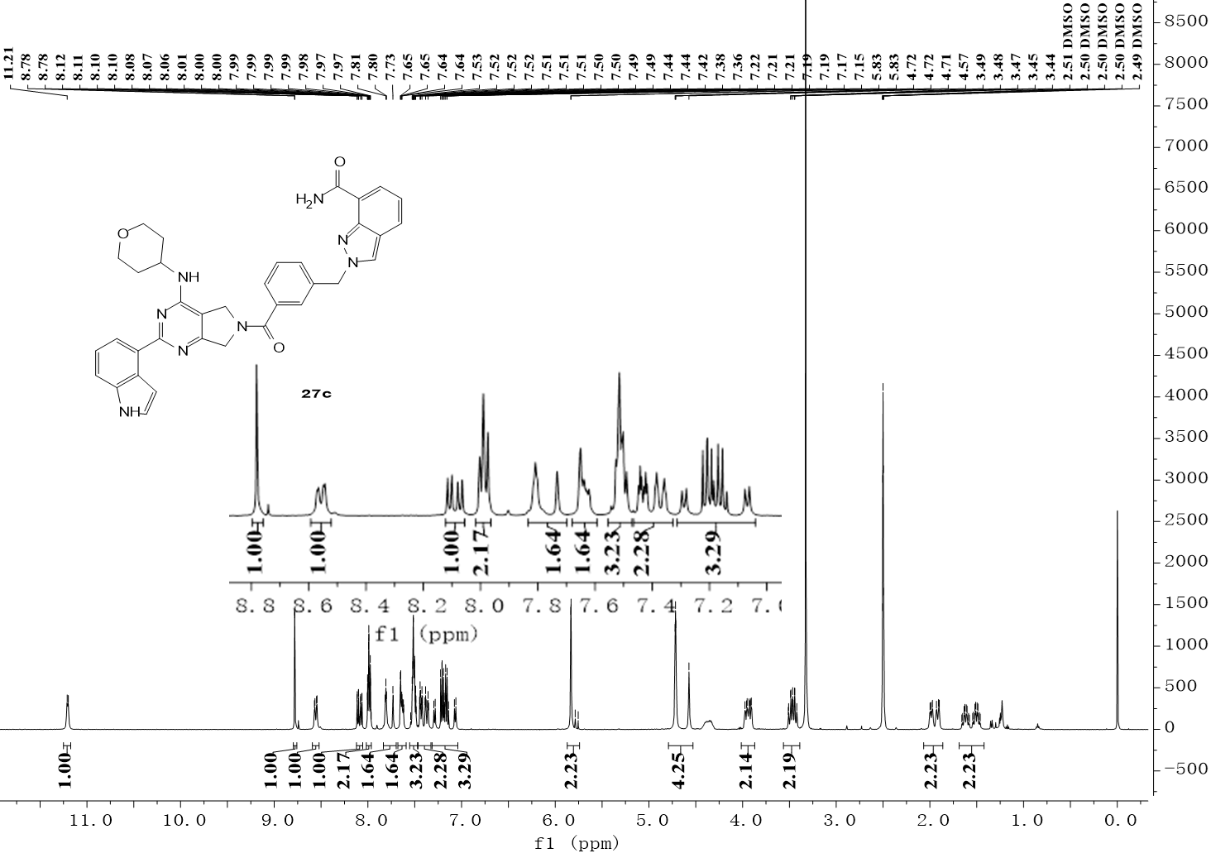

*(S)-2-(3-(2-(1H-indol-4-yl)-4-(3-methylmorpholino)-6,7-dihydro-5H-pyrrolo[3,4-d]py rimidine-6-carbonyl)benzyl)-2H-indazole-7-carboxamide (****27d****)*

*
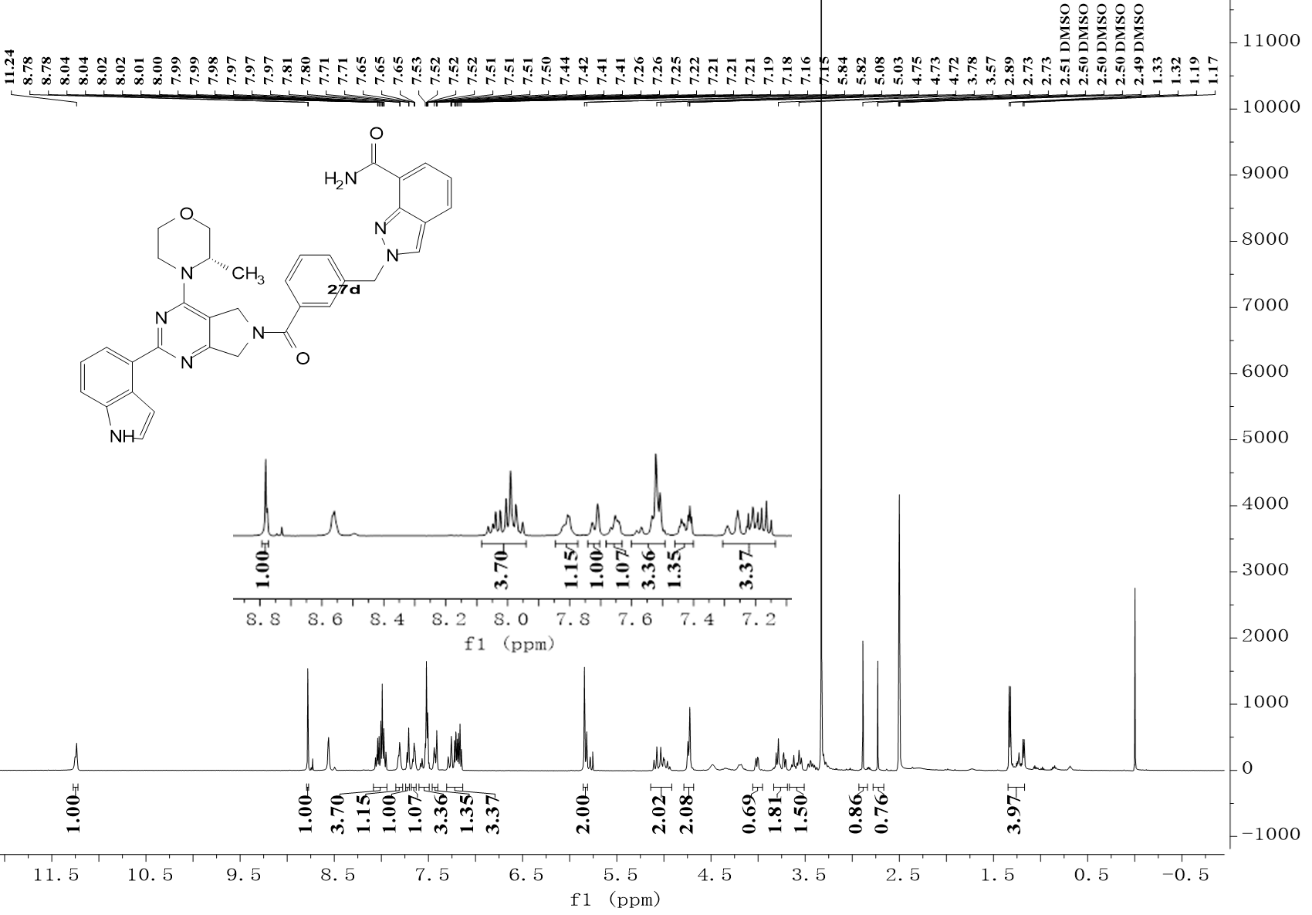
*

*2-(3-(2-(1H-indol-4-yl)-4-(tetrahydro-1H-furo[3,4-c]pyrrol-5(3H)-yl)-6,7-dihydro-5H-pyrrolo[3,4-d]pyrimidine-6-carbonyl)benzyl)-2H-indazole-7-carboxamide (****27e****)*


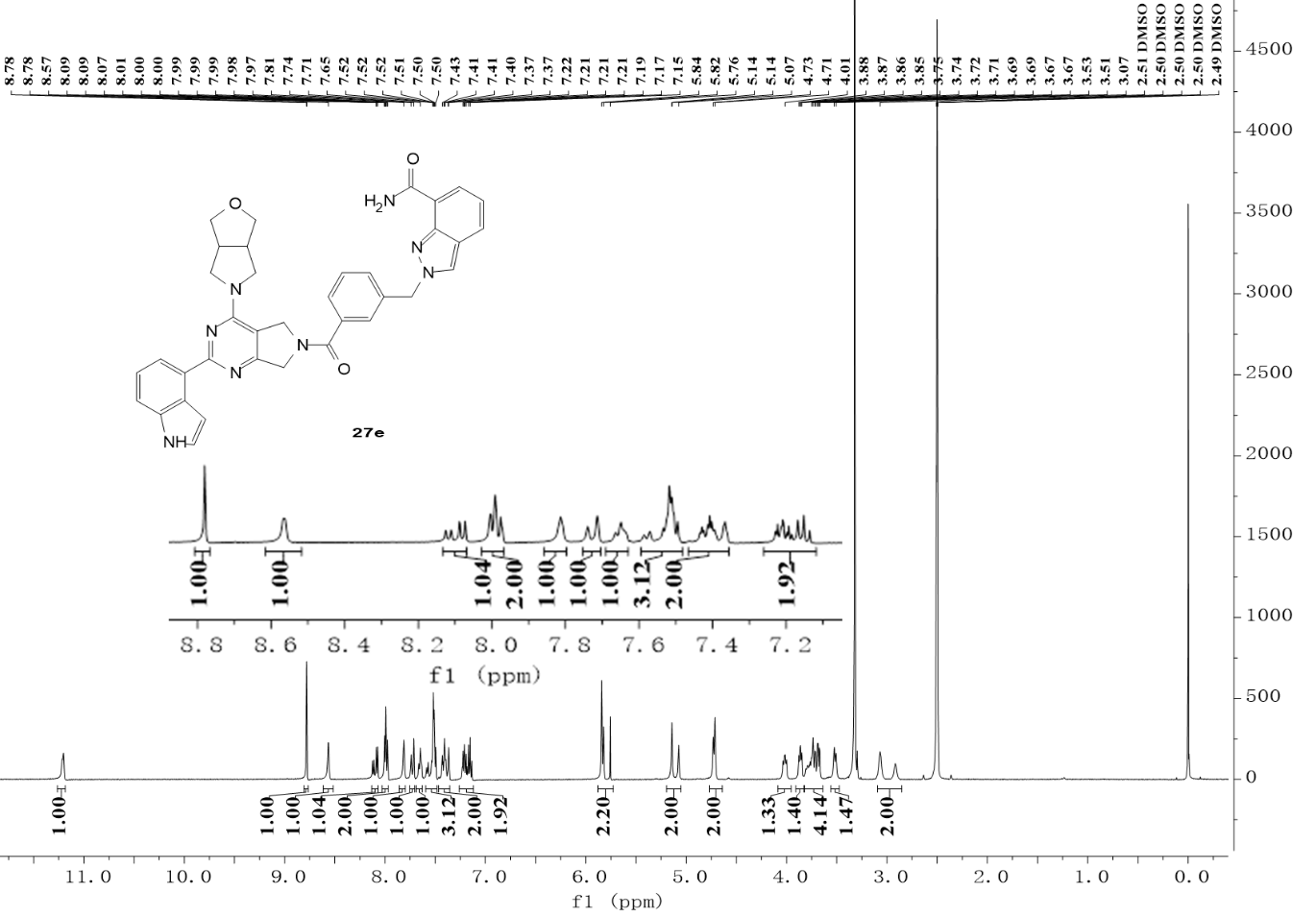

*2-(3-(2-(1H-indol-4-yl)-6,7-dihydro-5H-pyrrolo[3,4-d]pyrimidine-6-carbonyl)benzyl) -2H-indazole-7-carboxamide (****27f****)*


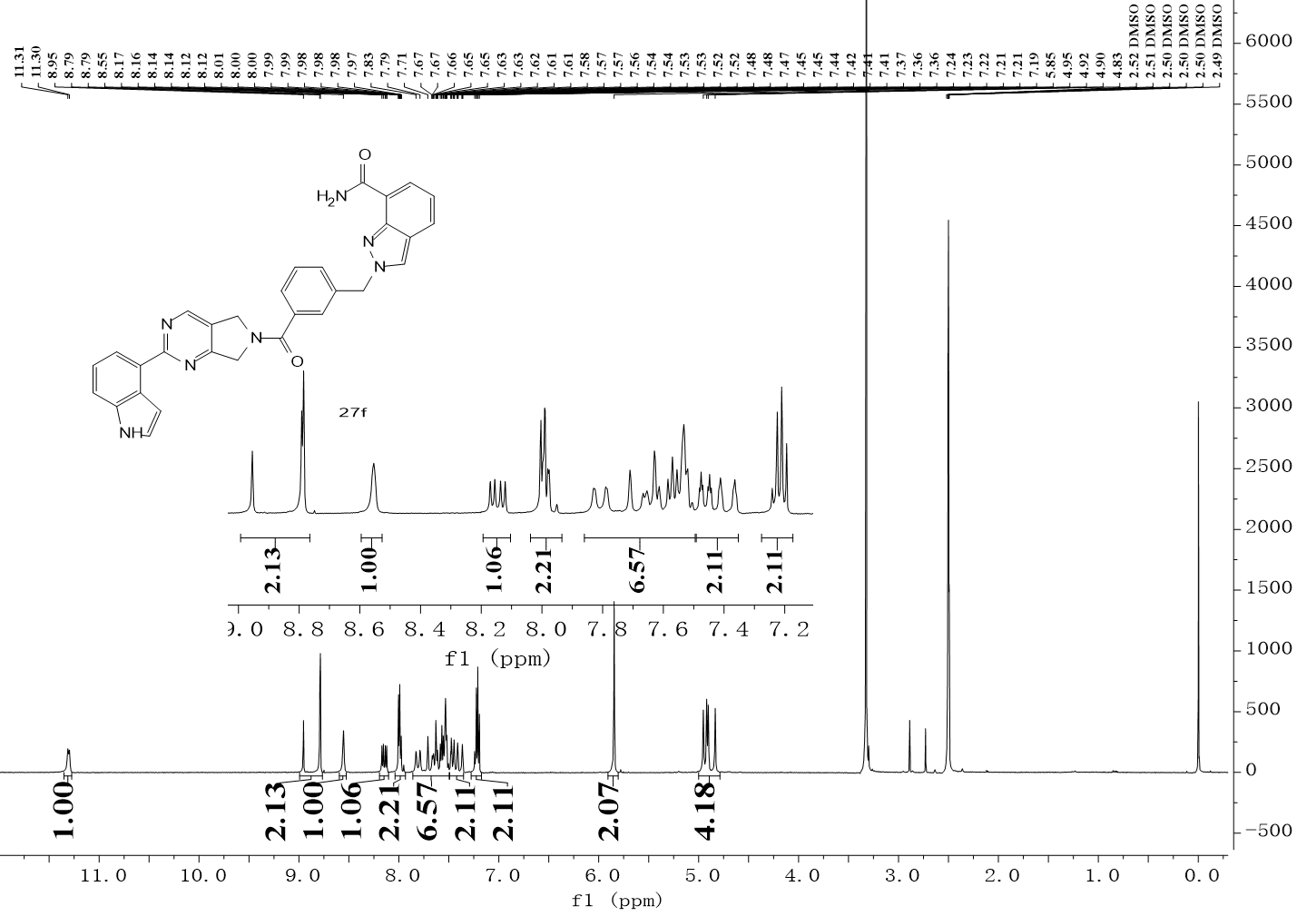

*2-(3-(4-(diethylamino)-2-(1H-indol-4-yl)-6,7-dihydro-5H-pyrrolo[3,4-d]pyrimidine-6-carbonyl)benzyl)-2H-indazole-7-carboxamide (****27g****)*

**
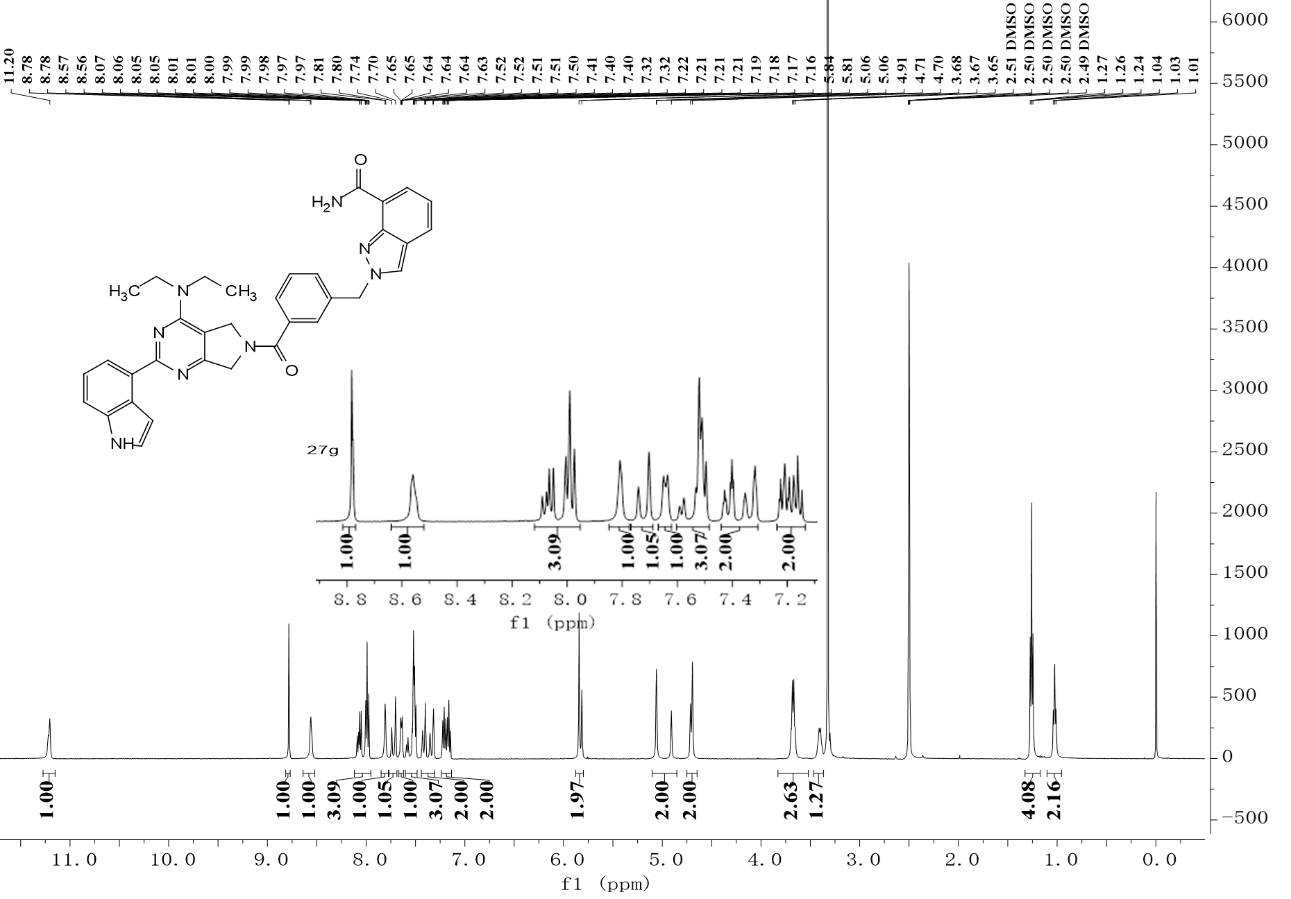
**

*2-(3-(4-(azetidin-1-yl)-2-(1H-indol-4-yl)-6,7-dihydro-5H-pyrrolo[3,4-d]pyrimidine-6-carbonyl)benzyl)-2H-indazole-7-carboxamide (****27h****)*

*2-(3-(4-(cyclopropylamino)-2-(1H-indol-4-yl)-6,7-dihydro-5H-pyrrolo[3,4-d]pyramid ine-6-carbonyl)benzyl)-2H-indazole-7-carboxamide (****27i****)*

*2-(3-(2-(1H-indol-4-yl)-4-(oxetan-3-ylamino)-6,7-dihydro-5H-pyrrolo[3,4-d]pyramid ine-6-carbonyl)benzyl)-2H-indazole-7-carboxamide (****27j****)*

**
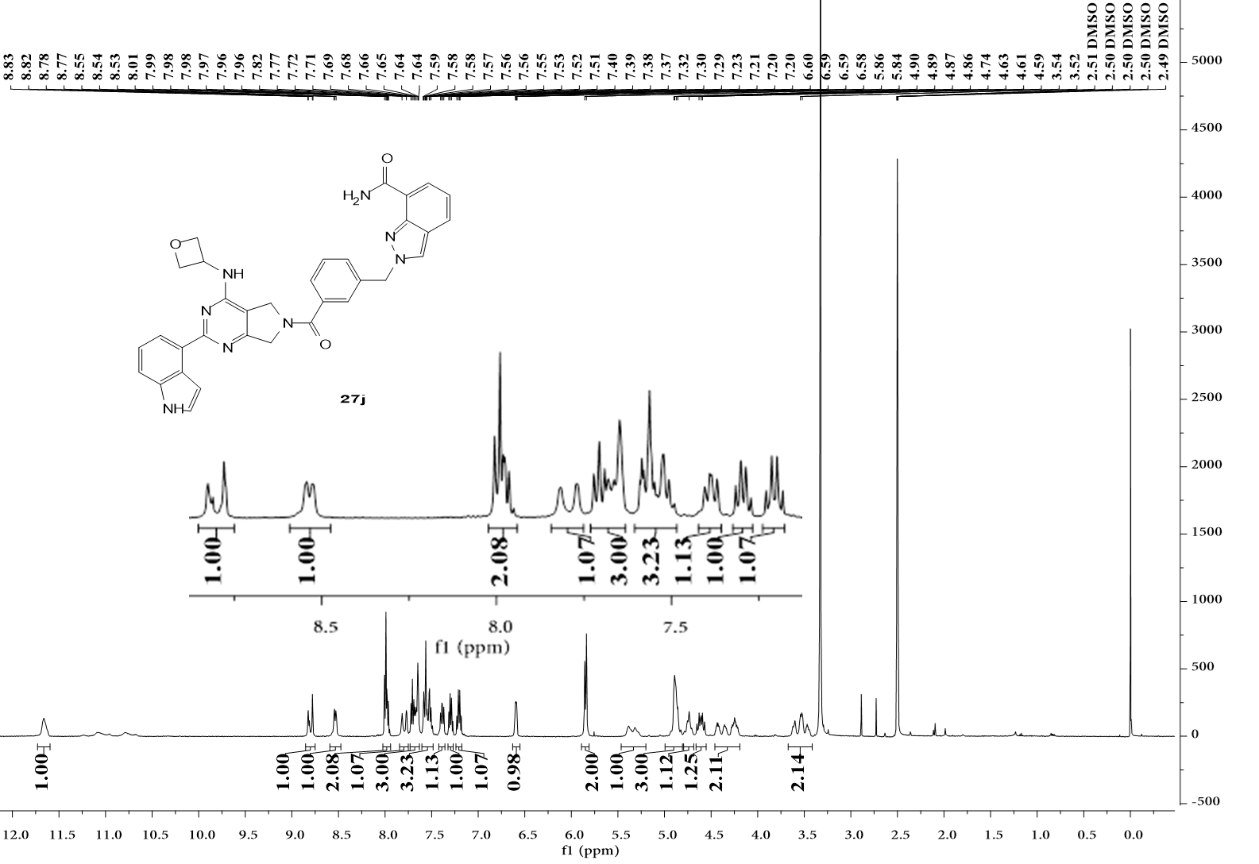
**

*2-(3-(2-(1H-indol-4-yl)-4-(pyrrolidin-1-yl)-6,7-dihydro-5H-pyrrolo[3,4-d]pyrimidine-6-carbonyl)benzyl)-2H-indazole-7-carboxamide (****27k****)*

**
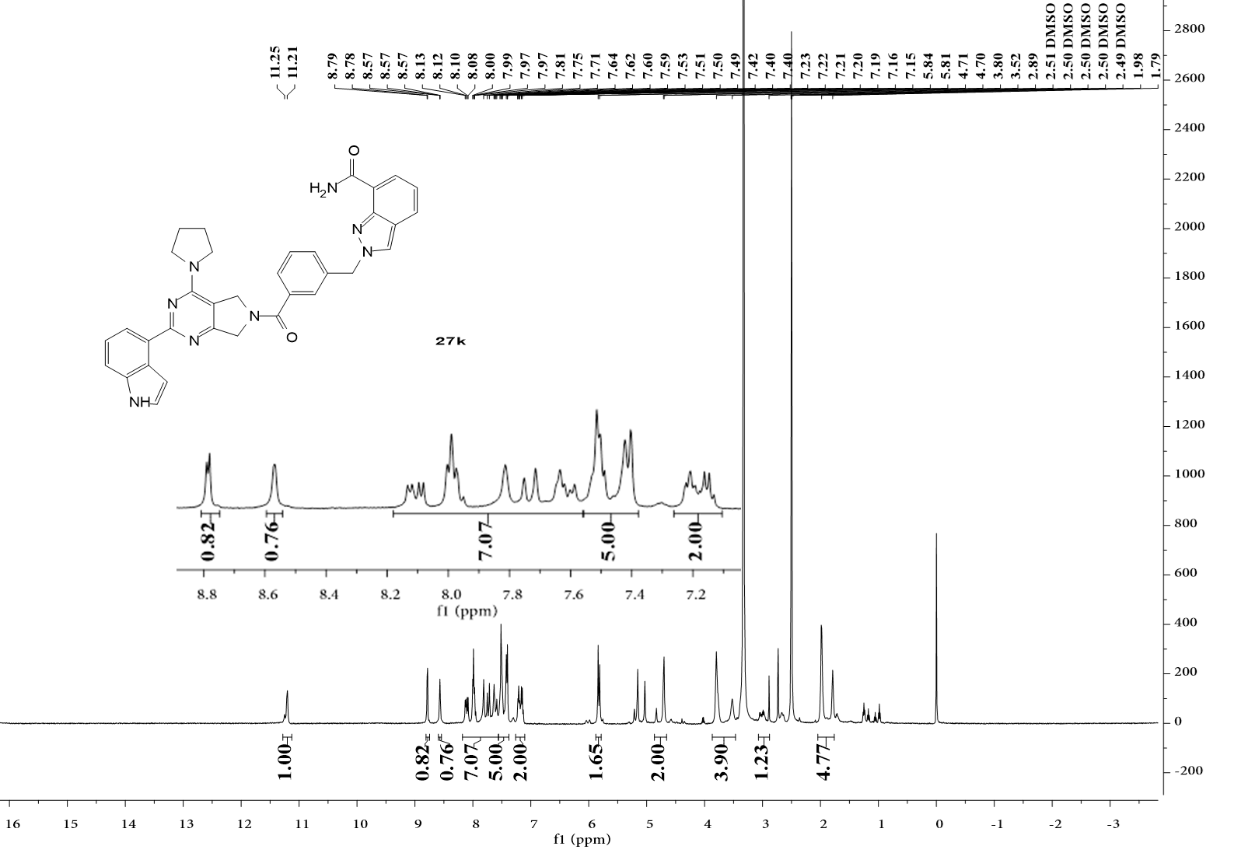
**

*(R)-2-(3-(2-(1H-indol-4-yl)-4-(3-methylmorpholino)-6,7-dihydro-5H-pyrrolo[3,4-d]py rimidine-6-carbonyl)benzyl)-1H-benzo[d]imidazole-7-carboxamide (****33a****)*

*(R)-2-(4-(2-(1H-indol-4-yl)-4-(3-methylmorpholino)-6,7-dihydro-5H-pyrrolo[3,4-d]py rimidine-6-carbonyl)benzyl)-1H-benzo[d]imidazole-7-carboxamide (****33b****)*

*(R)-2-(4-(2-(1H-indol-4-yl)-4-(3-methylmorpholino)-6,7-dihydro-5H-pyrrolo[3,4-d]py rimidine-6-carbonyl)phenyl)-1H-benzo[d]imidazole-7-carboxamide (****38a****)*


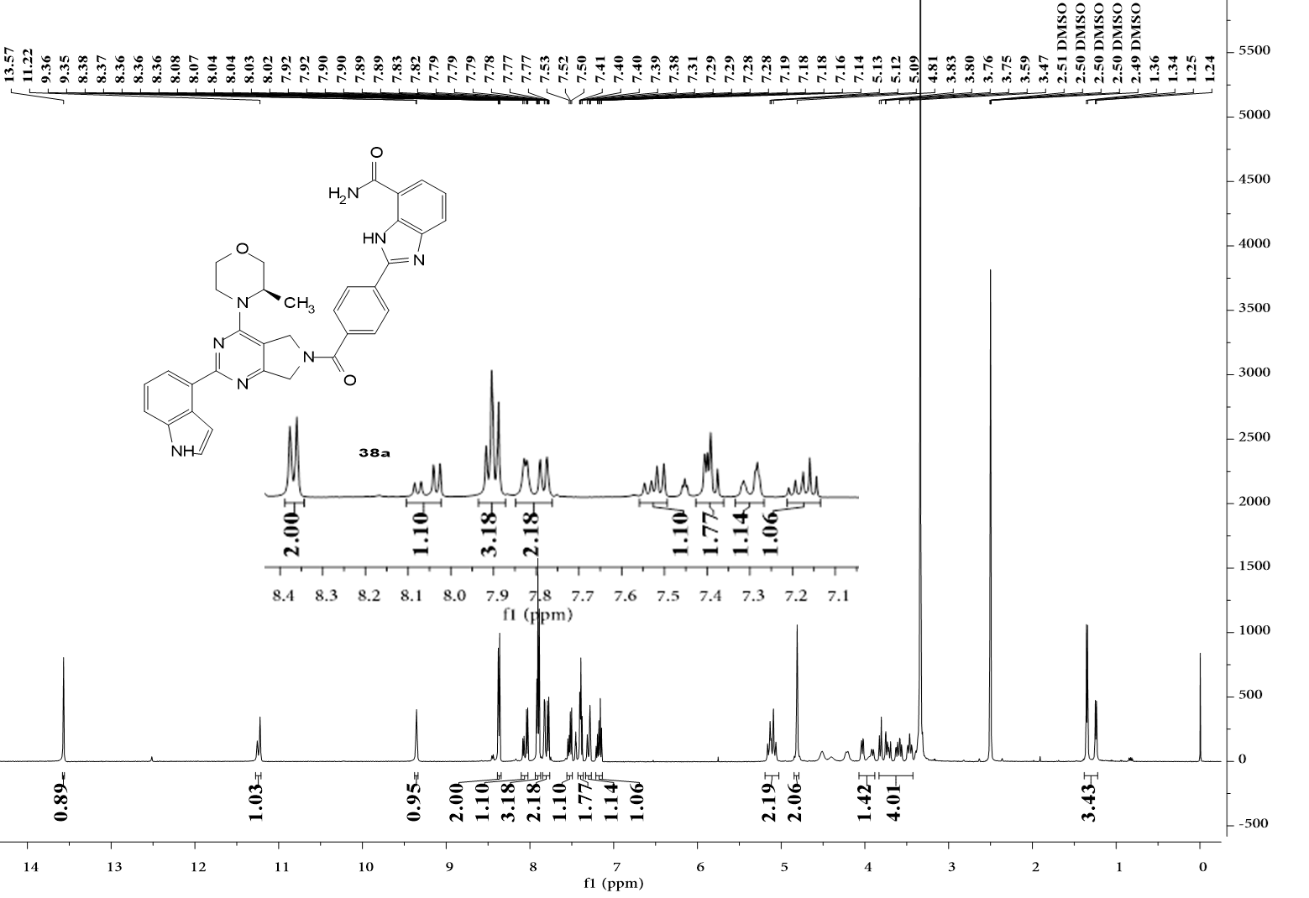

*(R)-2-(4-((2-(1H-indol-4-yl)-4-(3-methylmorpholino)-5,7-dihydro-6H-pyrrolo[3,4-d]p yrimidin-6-yl)methyl)phenyl)-1H-benzo[d]imidazole-7-carboxamide (****38b****)*


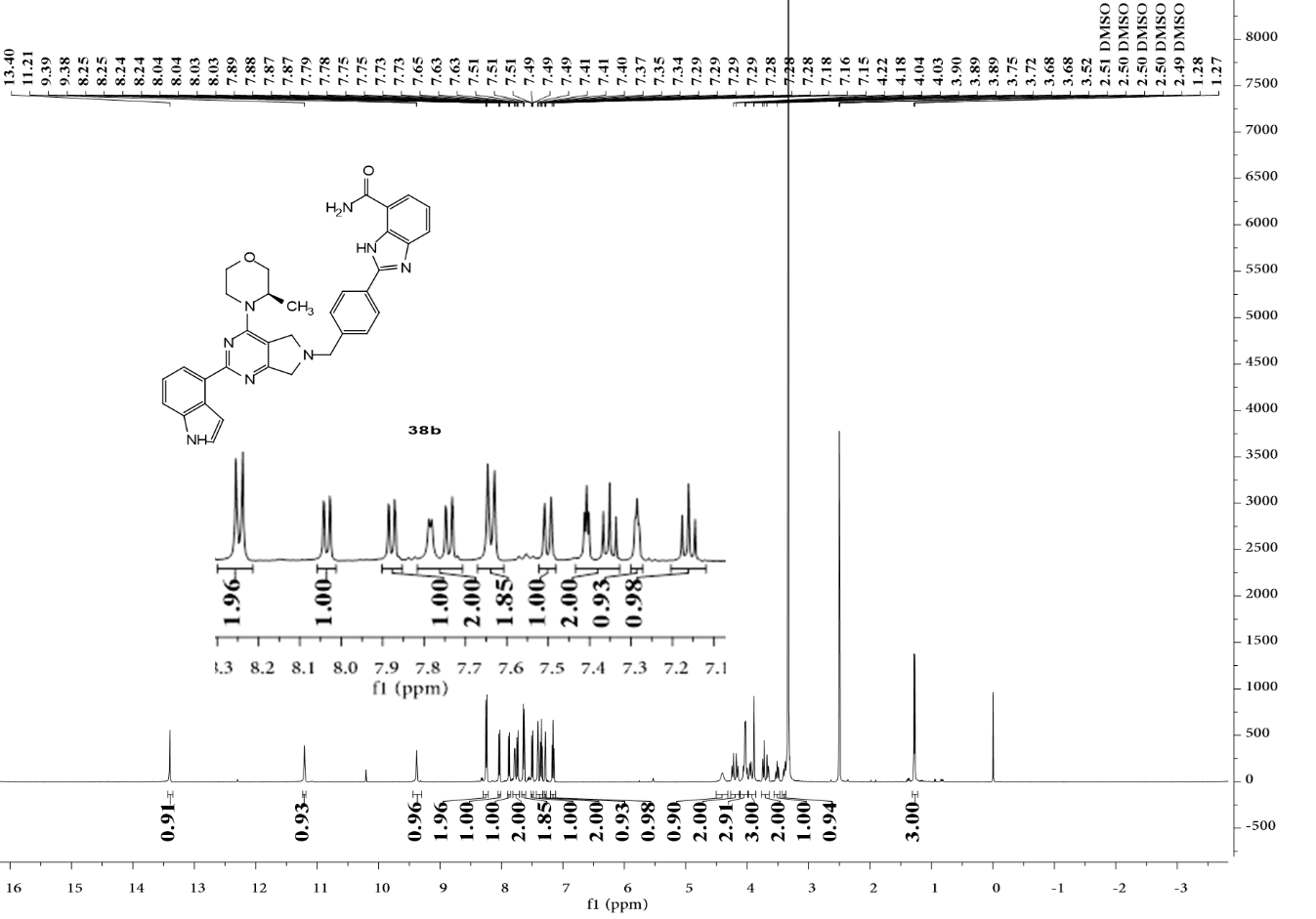

*(R)-2-((4-(2-(1H-indol-4-yl)-4-(3-methylmorpholino)-6,7-dihydro-5H-pyrrolo[3,4-d]p yrimidine-6-carbonyl)benzamido)methyl)-1H-benzo[d]imidazole-7-carboxamide (****42a****)*

**

*(R)-2-(1-(4-(2-(1H-indol-4-yl)-4-(3-methylmorpholino)-6,7-dihydro-5H-pyrrolo[3,4-d]pyrimidine-6-carbonyl)benzoyl)piperidin-4-yl)-1H-benzo[d]imidazole-7-carboxam ide (****42b****)*

**

*2-(1-(4-(2-(1H-indol-4-yl)-4-((R)-3-methylmorpholino)-6,7-dihydro-5H-pyrrolo[3,4-d]pyrimidine-6-carbonyl)benzoyl)pyrrolidin-3-yl)-1H-benzo[d]imidazole-7-carboxam ide (****42c****)*

**

*(R)-2-(1-(4-(2-(1H-indol-4-yl)-4-(3-methylmorpholino)-6,7-dihydro-5H-pyrrolo[3,4-d]pyrimidine-6-carbonyl)benzoyl)azetidin-3-yl)-1H-benzo[d]imidazole-7-carboxami de (****42d****)*

*(R)-2-(1-(3-(2-(1H-indol-4-yl)-4-(3-methylmorpholino)-6,7-dihydro-5H-pyrrolo[3,4-d]pyrimidine-6-carbonyl)benzoyl)azetidin-3-yl)-1H-benzo[d]imidazole-7-carboxami de (****43****)*

**Part 2: HRMS spectra of compounds 27a-27k, 33a-33b, 38a-38b, 42a-42d, 43.**

Sample Name: **27a**

HRMS (ESI): Calculated for C_35_H_32_N_8_O_3_ *m/z* (M + Na)^+^: 635.2490; found: 635.2496.

Sample Name: **27b**

HRMS (ESI): Calculated for C_34_H_30_N_8_O_3_ *m/z* (M + Na)^+^: 621.2333; found: 621.2330.

Sample Name: **27c**

HRMS (ESI): Calculated for C_35_H_32_N_8_O_3_ *m/z* (M + Na)^+^: 635.2490; found: 635.2484.

Sample Name: **27d**

HRMS (ESI): Calculated for C_35_H_32_N_8_O_3_ *m/z* (M + Na)^+^: 635.2490; found: 635.2489.

Sample Name: **27e**

HRMS (ESI): Calculated for C_36_H_32_N_8_O_3_ *m/z* (M + Na)^+^: 647.2490; found: 647.2501.

Sample Name: **27g**

HRMS (ESI): Calculated for C_34_H_32_N_8_O_2_ *m/z* (M + Na)^+^: 607.2540; found: 607.2526.

Sample Name: **27h**

HRMS (ESI): Calculated for C_33_H_28_N_8_O_2_ *m/z* (M + Na)^+^: 591.2227; found: 591.2234.

Sample Name: **27i**

HRMS (ESI): Calculated for C_33_H_28_N_8_O_2_ *m/z* (M + Na)^+^: 591.2227; found: 591.2222.

Sample Name: **27j**

HRMS (ESI): Calculated for C_33_H_28_N_8_O_3_ *m/z* (M + Na)^+^: 607.2177; found: 607.2175.

Sample Name: **27k**

HRMS (ESI): Calculated for C_34_H_30_N_8_O_2_ *m/z* (M + Na)^+^: 605.2384; found: 605.2384.

Sample Name: **33a**

HRMS (ESI): Calculated for C_35_H_32_N_8_O_3_ *m/z* (M + Na)^+^: 635.2490; found: 635.2487.

Sample Name: **38a**

HRMS (ESI): Calculated for C_34_H_30_N_8_O_3_ *m/z* (M + Na)^+^: 621.2333; found: 621.2337.

Sample Name: **38b**

HRMS (ESI): Calculated for C_34_H_32_N_8_O_2_ *m/z* (M + Na)^+^: 607.2540; found: 607.2537.

Sample Name: **42a**

HRMS (ESI): Calculated for C_36_H_33_N_9_O_4_ *m/z* (M + Na)^+^: 678.2548; found: 678.2542.

Sample Name: **42b**

HRMS (ESI): Calculated for C_40_H_39_N_9_O_4_ *m/z* (M + Na)^+^: 732.3017; found: 732.3008.

Sample Name: **42c**

HRMS (ESI): Calculated for C_39_H_37_N_9_O_4_ *m/z* (M + Na)^+^: 718.2861; found: 718.2853.

Sample Name: **42d**

HRMS (ESI): Calculated for C_38_H_35_N_9_O_4_ *m/z* (M + Na)^+^: 704.2704; found: 704.2705.

Sample Name: **43**

HRMS (ESI): Calculated for C_38_H_35_N_9_O_4_ *m/z* (M + Na)^+^: 704.2704; found: 704.2704.

**Part 3: HPLC purity data for the lead compound (38a).**

Analysis Report


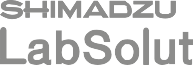

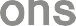

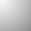


Sample Name: 38a

Column: Phenomenex Luna^®^ 5 mm C18(2) 100 Å 250 X 4.6 mm

Mobile phase: A: MeOH; B: H_2_O

Date: 2025-07-16

Injection volume: 6 uL

Flow rate: 0.5 mL/min

Method: 90% B to 10% B for 0.01 min stop at 9 min, 10% B to 90% B for 12 min stop at 20 min


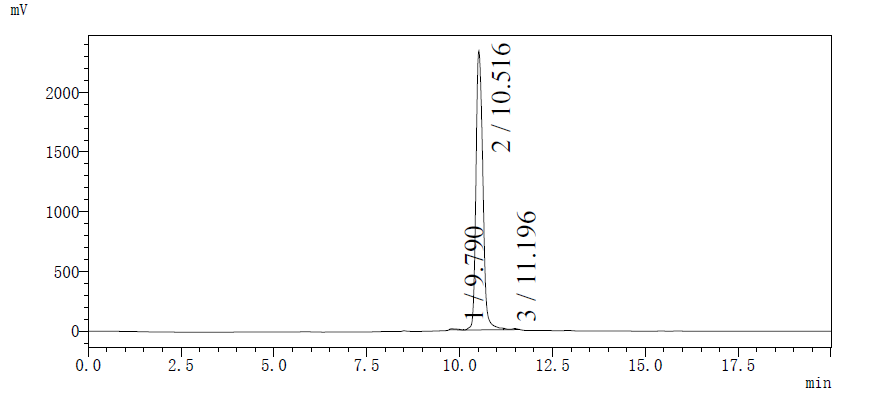


<Peak table>

Detector A Ch1 254nm

| Number | Retention time | Peak area | Peak area % |
| --- | --- | --- | --- |
| 1 | 9.790 | 122508 | 0.402 |
| 2 | 10.516 | 30225077 | 99.115 |
| 3 | 11.196 | 147325 | 0.483 |
| Total |  | 30494910 | 100.000 |

**Part 4: Western Blot.**


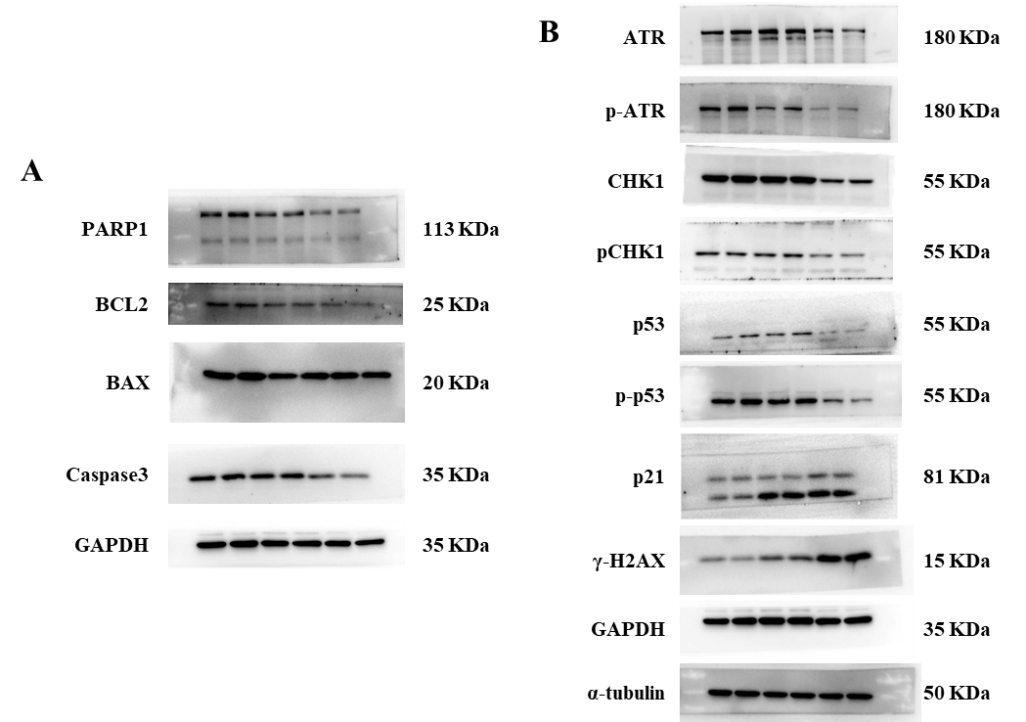


**Figure S1**. (A) The original western blot images of PARP1, BCL2, BAX and caspase-3 in MDA-MB-231 cells exposed to diﬀerent compounds for 48 h; (B) The original western blot images of ATR/p-ATR, CHK1/pCHK1, p53/p-p53, p21 and γH2AX in MDA-MB-231 cells exposed to diﬀerent compounds as indicated for 48 h.
